# Highly luminescent, biocompatible ytterbium(iii) complexes as near-infrared fluorophores for living cell imaging†
†Electronic supplementary information (ESI) available: Detailed photophysical and cell experiment data; ^1^H and ^19^F NMR spectra, UV-visible absorption spectra; IR spectra and MS spectra. See DOI: 10.1039/c8sc00259b


**DOI:** 10.1039/c8sc00259b

**Published:** 2018-03-19

**Authors:** Yingying Ning, Juan Tang, Yi-Wei Liu, Jing Jing, Yuansheng Sun, Jun-Long Zhang

**Affiliations:** a Beijing National Laboratory for Molecular Sciences , State Key Laboratory of Rare Earth Materials Chemistry and Applications , College of Chemistry and Molecular Engineering , Peking University , Beijing 100871 , P. R. China . Email: zhangjunlong@pku.edu.cn; b School of Chemistry , Beijing Institute of Technology , Beijing 100081 , P. R. China; c ISS Inc , Champaign , IL 61822 , USA

## Abstract

Herein, we report the design and synthesis of biocompatible Yb^3+^ complexes for near-infrared (NIR) living cell imaging. Upon excitation at either the visible (Soret band) or red region (Q band), these β-fluorinated Yb^3+^ complexes display high NIR luminescence (quantum yields up to 23% and 13% in dimethyl sulfoxide and water, respectively) and have higher stabilities and prolonged decay lifetimes (up to 249 μs) compared to the β-non-fluorinated counterparts. This renders the β-fluorinated Yb^3+^ complexes as a new class of biological optical probes in both steady-state imaging and time-resolved fluorescence lifetime imaging (FLIM). NIR confocal fluorescence images showed strong and specific intracellular Yb^3+^ luminescence signals when the biocompatible Yb^3+^ complexes were uptaken into the living cells. Importantly, FLIM measurements showed an intracellular lifetime distribution between 100 and 200 μs, allowing an effective discrimination from cell autofluorescence, and afforded high signal-to-noise ratios as firstly demonstrated in the NIR region. These results demonstrated the prospects of NIR lanthanide complexes as biological probes for NIR steady-state fluorescence and time-resolved fluorescence lifetime imaging.

## Abstract 2

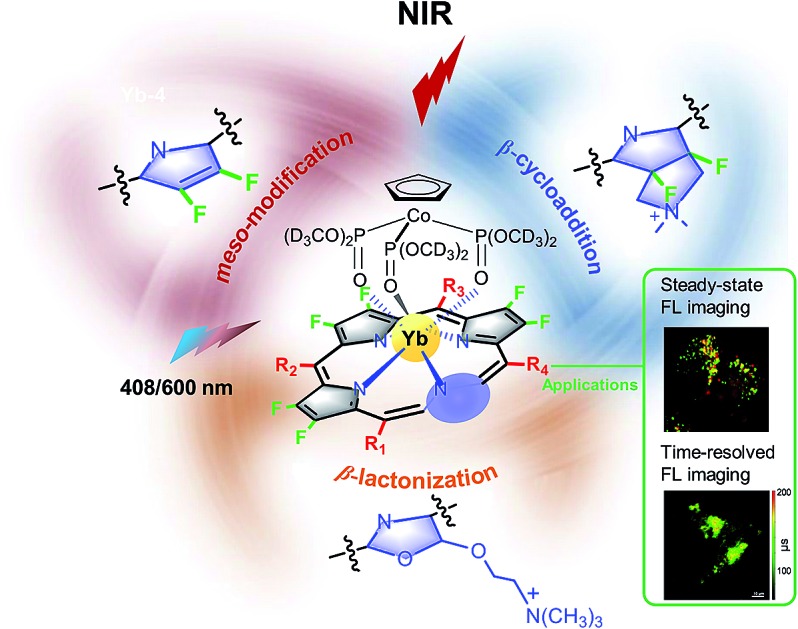
We report three synthetic methods to prepare biocompatible Yb^3+^ complexes, which displayed high NIR luminescence with quantum yields up to 13% in aqueous media. This renders β-fluorinated Yb^3+^ porphyrinoids a new class of NIR probes for living cell imaging including time-resolved fluorescence lifetime imaging.

## Introduction

Near-infrared (700–1700 nm) optical imaging has received increasing attention due to its ability to provide deeper tissue penetration, better signal-to-noise ratio and higher imaging resolution, which facilitates real-time visualization in clinical diagnoses and surgical operation.^1^–^5^ In this field, the critical chemical challenge is to develop NIR absorbent and emitting probes with good biocompatibility, high brightness and photostability. To meet this challenge, nanomaterials including conjugated polymers,^6^ surface-modified carbon nanotubes,^7^ quantum dots,^8^,^9^ metal–organic frameworks^10^ and upconversion nanoparticles^11^ have been employed for *in vivo* applications and the advantages of NIR imaging over traditional optical imaging in the visible region have been demonstrated. As nanomaterials may suffer from uncertain cytotoxicity and non-defined metabolism,^12^ the development of non-toxic and highly luminescent molecules with NIR optical imaging capabilities is of great importance. For small organic fluorophores, the design of “donor–acceptor–donor” type chromophores or π-conjugation extensions can effectively red-shift the absorption and emission to the NIR region,^13^,^14^ as exemplified by organic molecules such as indocyanine green (Cy),^15^ benzo-2,1,3-thiadiazole based molecules (FEB,^16^ IR-FGP,^17^ CH1055 18) and flavylium polymethine fluorophore (Flav)^19^ series, but can often be accompanied by low quantum yields. Alternatively, encouraged by the success of lanthanide (Ln) coordination chemistry in visible optical imaging (Eu^3+^, Tb^3+^),^20^–^24^ inorganic chemists have envisaged to expand the optical window to the NIR region by using NIR emissive Lns such as Nd^3+^, Sm^3+^ and Yb^3+^. Toward this goal, Maury and co-workers reported triazacyclononane Sm^3+^ and Yb^3+^ complexes for two-photon NIR-to-NIR imaging.^25^–^27^ Eliseeva and coworkers developed a polynuclear Sm^3+^ dendrimer for combined visible and NIR cell imaging.^28^ Pecoraro, Petoud and coworkers demonstrated Ln^3+^ (Ln = Nd, Yb) encapsulated sandwich metallacrowns for combined cell fixation and counter staining^29^ and necrotic cell imaging.^30^ Wong and co-workers also prepared a series of NIR emissive Yb^3+^ complexes^31^–^33^ and Yb^3+^ porphyrinate with conjugated rhodamine B for two-photon living cell imaging but in the visible region.^34^ The features of NIR Ln complexes including large Stokes shifts, characteristic elemental emissions^35^–^38^ and long decay lifetimes^39^–^41^ render them complementary to organic fluorophores, but the low quantum yield (<2.5%)^25^–^34^ arising from the forbidden f–f transitions and quenching effect of the high vibration X–H bond (X = C, N and O) is still a general issue remaining to be addressed.

To enhance the NIR emission of Yb^3+^, pioneering studies have been done by Seitz,^42^,^43^ Charbonnière^44^ and others,^45^–^47^ including replacing the high vibration energy X–H bond (X = C, O, N) with the X–D/F bond, extending the radiative lifetime of lanthanides and tuning the ligand to lanthanide sensitization process. Recently, we used perfluorinated porphyrins as antenna ligands, and deuterated Kläui ligand as an ancillary ligand to construct sandwiched Yb^3+^ complexes by removing the C–H bond close to the Yb^3+^ center.^48^ These Yb^3+^ complexes presented high NIR luminescence (900–1150 nm) with unprecedented quantum yields up to *ca.* 25% in CH_2_Cl_2_ (65% in CD_2_Cl_2_), long decay lifetimes of *ca.* 200 μs in CH_2_Cl_2_ (700 μs in CD_2_Cl_2_) and large extinction coefficients in both visible and red regions (10^5^–10^6^ M^–1^ cm^–1^ for the Soret band and 10^4^–10^5^ M^–1^ cm^–1^ for the Q band). These features render these Yb^3+^ complexes prospective NIR probes for both steady-state and time-resolved fluorescence lifetime imaging (FLIM). However, endowing these Yb^3+^ complexes with biocompatibility is still challenging due to their low solubility and cellular uptake after fluorination. Moreover, avoiding new X–H (X = C, N and O) bonds close to Yb^3+^ to dramatically quench NIR luminescence is also difficult in subsequent modifications. To address these issues, we herein report the synthesis of biocompatible Yb^3+^ complexes of β-fluorinated porphyrinates (**Yb-1–5**) through modification of the *meso*-phenyl and β-peripheral positions. Photophysical studies revealed that these Yb^3+^ complexes are the brightest NIR emissive biological fluorophores to date, with quantum yields of 9–23% in DMSO and 5–13% in H_2_O and decay lifetimes of 84–249 μs in DMSO and 56–173 μs in H_2_O under air-saturated conditions. They also possess much better photostability, as compared to their β-hydrogenated counterparts. As the excitation wavelength of the Yb^3+^ complexes is below 700 nm, which might be a disadvantage for thick tissue bioimaging, we herein demonstrate the first biological application in NIR living cell imaging. NIR confocal imaging microscopy showed strong and specific luminescence assigned to the ^2^F_5/2_ → ^2^F_7/2_ transition of Yb^3+^ in living cells upon excitation at *ca.* 408 and 600 nm, with preferential localization in the lysosome. Intracellular FLIM measurements on the microsecond scale demonstrated the use of **Yb-1–5** as NIR FLIM probes with high signal-to-noise ratios. Therefore, this work opens access to the design of biological NIR probes with capability of both steady-state fluorescence and time-resolved fluorescence lifetime imaging.

## Results and discussion

### Synthesis and characterization of Yb^3+^ complexes

**Scheme 1:**
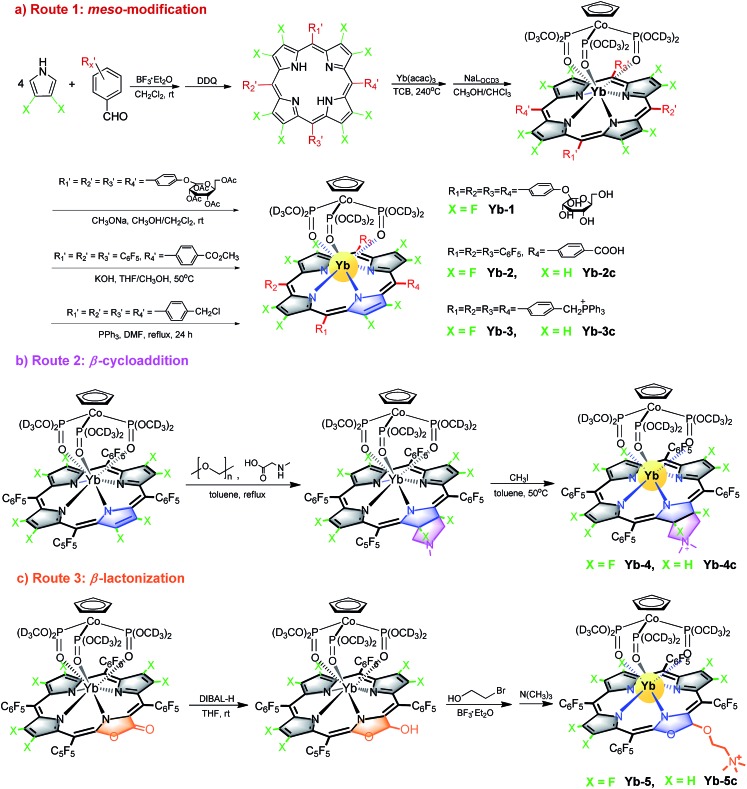
Synthetic routes for biocompatible β-fluorinated Yb^3+^ complexes **Yb-1–5** and β-hydrogenated analogues **Yb-2c–5c** studied in this work.

We designed biocompatible Yb^3+^ complexes through three synthetic strategies (Scheme 1). First, complexes **Yb-1–3** were obtained through modification of the *meso*-phenyl with water-soluble groups such as glycosyl, carboxyl and triphenylphosphonium, respectively. As previously reported, the substitution of *meso*-phenyl groups has subtle effects on the Yb^3+^ NIR emission.^48^ As shown in Scheme 1a, these complexes were synthesized by the condensation of corresponding benzaldehyde (or pentafluorobenzaldehyde) and 3,4-difluoropyrrole to porphyrin free bases. Consequent reaction of ytterbium acetylacetonate hydrate Yb(acac)_3_ with β-fluorinated porphyrin free bases afforded the desired Yb^3+^ complexes **Yb-1–3**, after hydrolysis or ionization (see the details in the Experimental section).

The second approach is 1,3-dipolar cycloaddition of the β-periphery of Yb^3+^ perfluorinated porphyrin with azomethine ylides (Scheme 1b). The advantage of this approach is direct functionalization of the β-periphery without the elimination of β-C–F bonds, which is important to maintain the high NIR luminescence. Further methylation of the amine to a quaternary ammonium afforded **Yb-4**.^49^^19^F-NMR spectrum and high-resolution electrospray ionization mass spectrometry demonstrated that β-C–F bond is intact after 1,3-dipolar cycloaddition.

Third, following our continued interest in porpholactone,^50^ we reduced the β-azlactone moiety to azlactol, which can attach a trimethyl-*N*-ethylammonium moiety, and afforded **Yb-5** (Scheme 1c). As controls, we also synthesized β-non-fluorinated Yb^3+^ complexes (**Yb-2c–5c**) based on tetraphenylporphyrin analogues following similar procedures. To better compare β-fluorination effect, all complexes used deuterated Kläui's ligand as the ancillary ligand.^51^,^52^ Their structures were characterized by ^1^H, ^19^F-NMR, UV-vis and IR spectroscopies and mass spectrometry. Detailed synthetic procedures and characterizations are presented in the Experimental section and ESI (Fig. S1–60†).

### Photophysical properties of Yb^3+^ complexes in the NIR region

**Fig. 1:**
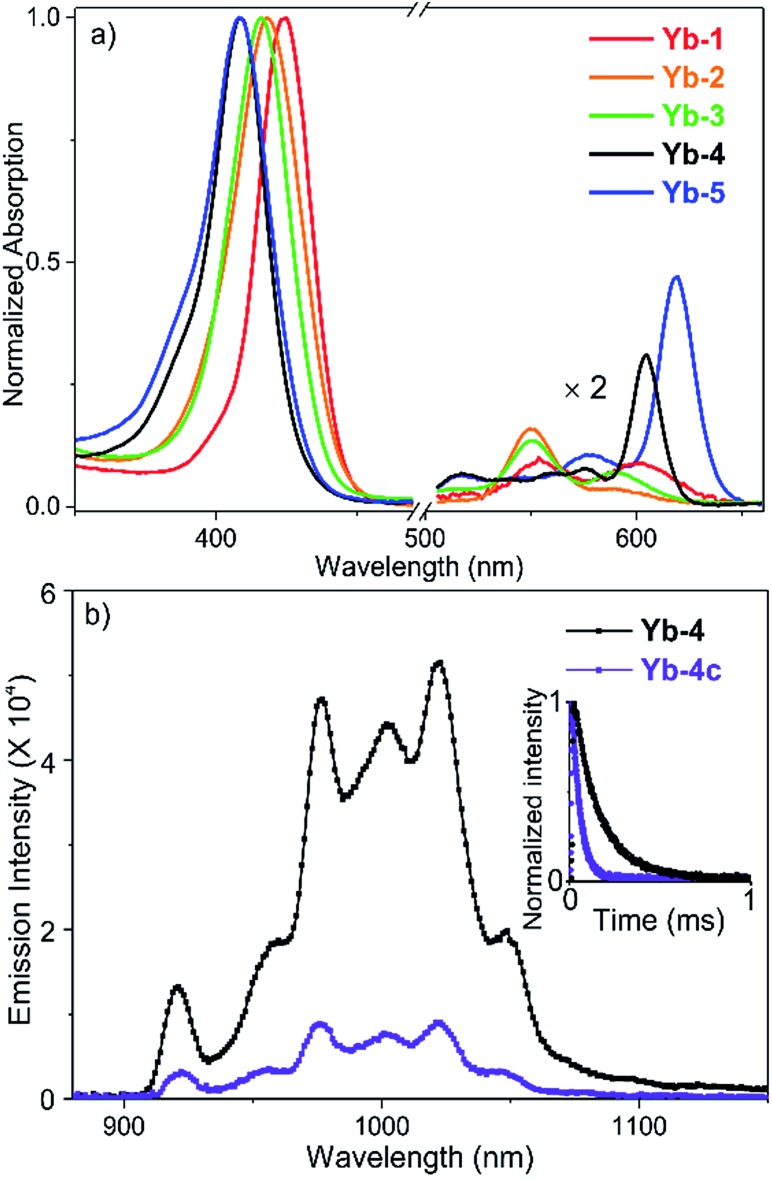
(a) Normalized absorption spectra of **Yb-1–5** in water with 0.1% DMSO; (b) emission spectra of **Yb-4** and **Yb-4c** in water (0.1% DMSO, *λ*_ex_ = 410 nm, *A*_410nm_ = 0.1). Inset: decay curves of **Yb-4** and **Yb-4c** in water monitored at 980 nm.

The absorption spectra of **Yb-1–3** present similar profiles in H_2_O with 0.1% DMSO (Fig. 1a) and display intense Soret bands centered at *ca.* 410 nm and Q bands between 500 and 620 nm, indicating the subtle effect of substitution of the *meso*-phenyl group on the electronic structures. Through β-modification, the Q(0,0) bands of **Yb-4** and **Yb-5** extend to 603 and 620 nm, respectively, accompanied by increasing extinction coefficients. This closely resembles the absorption spectra of chlorin-type porphyrinoids with one saturated pyrrole ring. Compared to **Yb-1–5**, the β-hydrogenated counterparts display similar absorption with slightly bathochromic shifts (Fig. S61†). NIR luminescence of **Yb-1–5** and **Yb-2c–5c** was measured in air-saturated and degassed dimethyl sulfoxide (DMSO) and H_2_O, and the data are summarized in Table 1 and Fig. S62–71.† The quantum yields were determined using a comparative method with the reference 5,10,15,20-tetraphenylporphyrin-Yb(iii)-[(cyclopentadienyl)tris(di(ethyl)phosphito)cobaltate] (YbTPP(L_OEt_), *Φ*_Δ_ = 0.024 in CH_2_Cl_2_).^53^ Experimental relative errors of the lifetimes and quantum yields are ±5% and ±10% respectively. Upon excitation at the Soret or Q band, **Yb-1–5** exhibited strong and characteristic Yb^3+^ emission at *ca.* 900–1200 nm, assigned to the ^2^F_5/2_ → ^2^F_7/2_ transition. The two peaks centered at *ca.* 918 and 957 nm are assigned as hot bands as they dramatically decreased at low temperature (Fig. S72†), while the other four are the f–f transition peaks. The quantum yields (*Φ*_Yb_) of **Yb-1–5** are 9–23% in DMSO and 5–13% in H_2_O under air-saturated conditions, with long lifetimes (*τ*_obs_s) of *ca.* 84–249 μs in DMSO and 56–173 μs in H_2_O, much higher than their β-hydrogenated counterparts (*Φ*_Yb_ and *τ*_obs_ typically below 2.5% and 38 μs in H_2_O, respectively). Taking **Yb-4** as an example (Fig. 1b), the *Φ*_Yb_ in H_2_O reaches 10%, with a *τ*_obs_ up to 140 μs, which is 4-fold higher than that of **Yb-4c** (*Φ*_Yb_ = 2.3%, *τ*_obs_ = 35 μs in H_2_O). With increasing molecular hydrophilicity (Table S1†), decreasing quantum yield and lifetime were observed due to solvation quenching. Among **Yb-1–5**, **Yb-2** showed the highest *Φ*_Yb_ (13%) and longest *τ*_obs_ (173 μs) in H_2_O. In degassed H_2_O, **Yb-1-4** and **Yb-2c–4c** exhibited decay lifetimes of 26–175 μs and quantum yields of 1.4–13% (Table 1), similar to those in the air-saturated solutions. In degassed DMSO, the decay lifetimes and quantum yields are slightly higher, within the average difference of 10%. However, for **Yb-5** and **Yb-5c**, the excited state lifetimes and NIR luminescence quantum yields increased dramatically in degassed solution. For example, the decay lifetimes of **Yb-5** were 84(4) μs in DMSO and 64(2) μs in water and increased to 130(6) and 78(3) μs, respectively, in the absence of oxygen.

**Table 1:** Decay lifetime (*τ*_obs_) monitored at 980 nm and quantum yields (*Φ*_Yb_) of **Yb-1–5** and **Yb-2c–5c** in air-saturated and degassed DMSO and H_2_O^*a*^ ^,^^*b*^

Comp.	DMSO				H_2_O^*c*^			
	Air-equil.		Degassed		Air-equil.		Degassed	
	*τ*_obs_ (μs)	*Φ*_Yb_ (%)	*τ*_obs_ (μs)	*Φ*_Yb_ (%)	*τ*_obs_ (μs)	*Φ*_Yb_ (%)	*τ*_obs_ (μs)	*Φ*_Yb_ (%)
**Yb-1**	161(5)	16(2)	165(4)	16(2)	95(2)	7.6(0.4)	96(3)	7.7(0.5)
**Yb-2**	249(10)	23(2)	251(8)	23(3)	173(5)	13(1)	175(6)	13(1)
**Yb-3**	205(2)	20(1)	213(5)	21(2)	56(2)	5.1(0.2)	58(3)	5.2(0.3)
**Yb-4**	173(4)	17(2)	187(8)	18(2)	140(5)	10(1)	142(5)	10(1)
**Yb-5**	84(4)	9.0(1)	130(6)	13(1)	64(2)	6.0(0.3)	78(3)	7.1(0.4)
**Yb-2c**	52(2)	6.2(0.2)	52(2)	6.0(0.2)	38(1)	2.5(0.2)	41(2)	2.6(0.2)
**Yb-3c**	49(1)	5.6(0.2)	51(2)	5.6(0.2)	25(1)	1.4(0.1)	26(2)	1.4(0.1)
**Yb-4c**	49(2)	5.4(0.2)	50(2)	5.5(0.2)	35(1)	2.3(0.2)	38(2)	2.4(0.1)
**Yb-5c**	41(2)	4.0(0.3)	52(2)	5.6(0.2)	32(2)	1.6(0.2)	42(3)	2.5(0.2)

^*a*^Standard error values are given in parentheses; they refer to the reproducibility of the measurements. The estimated uncertainties in the quantum yield are 15%. Experimental relative errors: *τ*_obs_, ±5%; *Φ*_Yb_ ±10%.

^*b*^Quantum yields were determined using a comparative method and referenced to Yb(TPP) (L_OEt_) (*Φ*_Δ_ = 2.4% in CH_2_Cl_2_).

^*c*^Measured with 0.1% DMSO.

### Stability in aqueous media

**Fig. 2:**
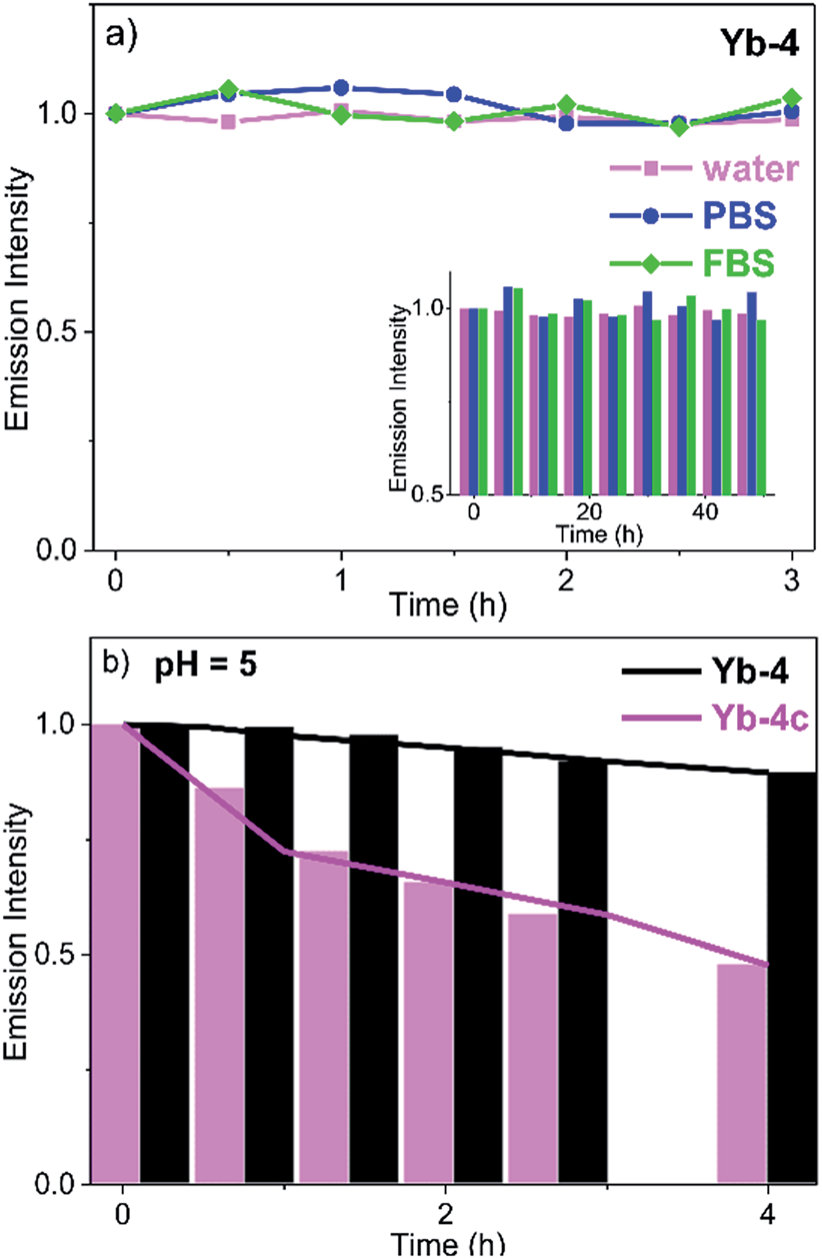
(a) Spectroscopic evaluation of **Yb-4** photostability in water, PBS and FBS with 0.1% DMSO under irradiation of a 405 nm laser (0.2 W cm^–2^) (inset: dark stability); (b) spectroscopic evaluation of **Yb-4** and **Yb-4c** stabilities in pH = 5 PBS with 0.1% DMSO under irradiation of a 405 nm laser (0.2 W cm^–2^). Emission intensity values were referenced to the emission intensity of Yb^3+^ (980 nm) at 0 h.

In DMSO (Fig. S73†) and H_2_O (Fig. S74†), the absorption of Yb^3+^ complexes showed negligible change after 48 h, indicating that they are stable and no decomposition was observed under such conditions. We also measured the ^2^D NMR of **Yb-4** in CH_3_OH as an example (Fig. S75†) and only one single peak was observed. This demonstrated that there is no H/D exchange of the deuterated ligand in protonated solvents. To further demonstrate the potential ability of Yb^3+^ porphyrinates as imaging agents, we investigated the stability of these Yb^3+^ complexes by monitoring the luminescence in the presence/absence of light in water, phosphate buffer (PBS), and fetal bovine serum (FBS). Interestingly, we found that β-fluorinated compounds are more stable than their β-non-fluorinated analogues. **Yb-4** showed high dark stability over 48 h (Fig. 2), and the NIR luminescence did not significantly change over a long period of time under continuous irradiation (405 nm laser, 0.2 W cm^–2^). At pH = 5 (lysosome pH), **Yb-4** was photostable over 4 h, while **Yb-4c** showed obvious photobleaching (55%). These results suggest that β-fluorination enhances the stability of Yb^3+^ porphyrinates in aqueous media.^54^

### Lipophilicity

To investigate the β-fluorination effect on the lipophilicity, the logarithm of *n*-octanol/water partition coefficient (log *P*) for **Yb-1–5** and **Yb-2c–5c** was measured. As shown in Table S1,† β-fluorinated complexes exhibit higher log *P* values than the corresponding β-hydrogenated complexes. The results indicated that β-fluorination increases the lipophilicity of the Yb^3+^ complexes. Additionally, the log *P* values of **Yb-3** and **Yb-3c** are significantly lower than those of other single-charged complexes. This provides useful information to understand the cellular uptake and subcellular localization in the context.

### Cytotoxicity

We measured the cytotoxicity of the complexes in HeLa cells. The cell viability was over 80% in the dark even at a concentration of 20 μM (Fig. S76†). Upon light irradiation for 30 min (400–700 nm, 6.5 mW cm^–2^), **Yb-1–4** showed negligible photocytotoxicity toward HeLa cells at a concentration of 10 μM (cell viability >80%), while **Yb-5** showed an IC_50_ value (the dye concentration required to kill 50% of the cells) as low as 1.0 μM (Fig. S77†). Singlet oxygen detection experiment revealed that the photocytotoxicity of **Yb-5** may be ascribed to the singlet oxygen generation upon light irradiation (Fig. S78†). This results from the inefficient energy transfer from the triplet state of perfluorinated porpholactol (*ca.* 11 494 cm^–1^)^55^ to the excited state of Yb^3+^ (*ca.* 10 250 cm^–1^).^56^–^58^ The singlet oxygen quantum yield of **Yb-5** was further estimated to be 0.36, referenced to the tetraphenylporphyrin (TPP, *Φ*_Δ_ = 0.55 in CHCl_3_),^59^ by the emission centered at approximately 1270 nm derived from the ^1^Δ_g_ → ^3^Σ_g_ transition of ^1^O_2_ (Fig. S78†). The virtues of **Yb-5** including strong absorption in the NIR region and high singlet oxygen efficiency indicated its potential application as a dual-modal imaging and photodynamic therapy probe in the future study.

### NIR steady-state fluorescence imaging

**Fig. 3:**
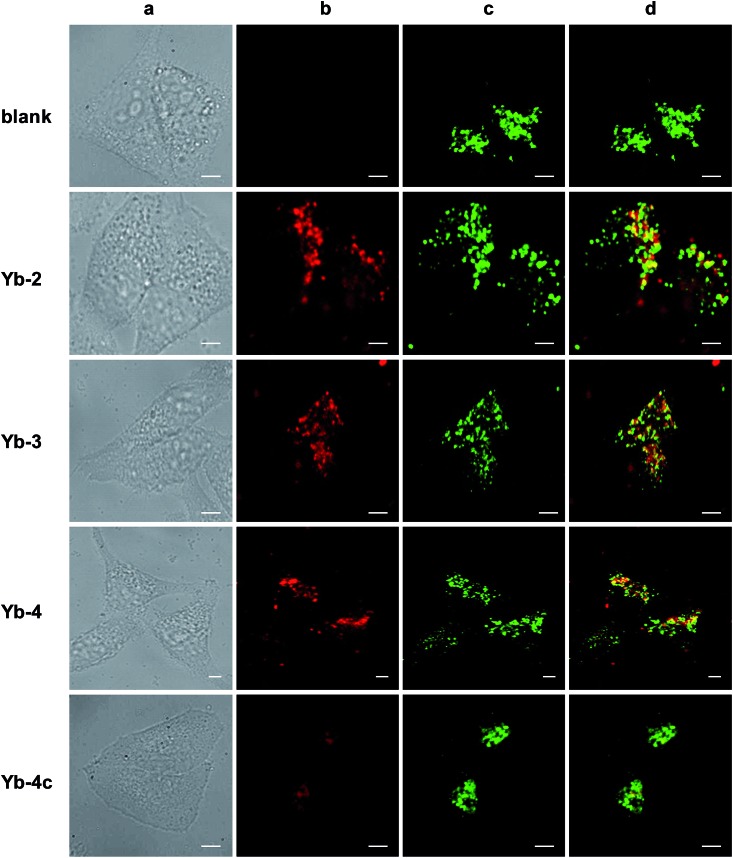
NIR confocal images performed on HeLa cells incubated with 10 μM of the corresponding Yb^3+^ complexes for 12 h followed by 30 min incubation with 75 nM LysoTracker Green. (a) Bright field; (b) NIR signal arising from Yb^3+^ in channel 1 (*λ*_ex_, 408 nm; *λ*_em_, 935/170 nm bandpass); (c) visible signal arising from LysoTracker Green in channel 2 (*λ*_ex_, 470 nm; *λ*_em_, 530/43 nm bandpass); (d) merged b and c showing colocalisation (*P* = 0.75 for **Yb-2**, 0.78 for **Yb-3**, 0.67 for **Yb-4**, 0.29 for **Yb-4c**). Scale bar: 10 μm.

Cell imaging experiments (Fig. 3) were performed at a 10 μM concentration of **Yb-1–4** using an Alba5 FLIM/FFS confocal microscopy imaging system (ISS Inc., Champaign, Illinois, USA) (Fig. S79†). A stock solution of Yb^3+^ complex in chromatographic grade, anhydrous DMSO was used and the concentration was 2 mM. The solution was diluted to a final concentration of 10 μM using the complete growth medium. The Yb^3+^ NIR signal was collected in channel 1 using a single photon avalanche photodiode detector (SPAD) manufactured by Excelitas (model SPCM-AQRH-15, quantum efficiency >25% at 950 nm), while that from LysoTracker® Green DND-26 was collected in channel 2 using a GaAsP photomultiplier tube detector manufactured by Hamamatsu (Model H7422p-40). The detection wavelength of SPAD is up to 1050 nm, with typically low dark noise (50 cps).

As shown in Fig. 3, upon excitation at the Soret band (408 nm laser, 4 μW cm^–2^), we observed intense intracellular NIR luminescence in channel 1 using 935/170 nm bandpass for **Yb-2–4**, assigned to ^2^F_5/2_ → ^2^F_7/2_ transition from Yb^3+^. However, we could not observe such luminescence for **Yb-1** (Fig. S80†). The results of inductively coupled plasma spectrometry (ICP) showed that 0.1–0.2 μg mL^–1^ Yb^3+^ could be detected for **Yb-2–4**, while <0.02 μg mL^–1^ for **Yb-1** (Table S2†), suggesting that **Yb-1** could not be uptaken by the cells under this condition. The exact mechanism why **Yb-1** could not enter living cells is not clear, but the results indicated the importance of choice of biocompatible conjugates in the design of Yb^3+^ NIR probes.

The images of **Yb-2–4** show high signal-to-noise ratios, as the background fluorescence is dramatically reduced in the NIR region. These complexes preferentially colocalized in the lysosome, as revealed by the good colocalization of NIR fluorescence with the fluorescence of the lysosome tracker. Further modifications are required to achieve different intracellular localization profiles.^60^–^63^ As controls, incubation HeLa cells with **Yb-2c–4c** showed much less intense NIR luminescence than **Yb-2–4**, as shown in Fig. 3 and S81,† even ICP showed much higher cellular uptake of **Yb-2c–4c** than **Yb-2–4** (Table S2†). These results suggested that β-fluorination improves NIR luminescence in living cells, consistent with the *in vitro* photophysical properties.

**Fig. 4:**
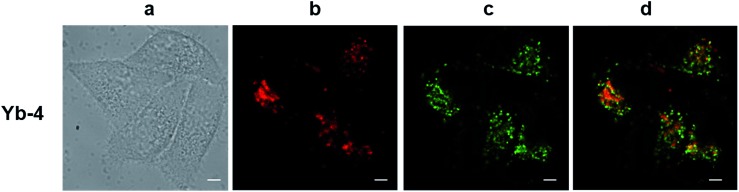
NIR confocal images performed on living HeLa cells incubated with 10 μM **Yb-4** for 12 h followed by 30 min incubation with 75 nM LysoTracker Green. (a) Bright field; (b) NIR signal arising from Yb^3+^ in channel 1 (*λ*_ex_, 620 nm; *λ*_em_, 935/170 nm bandpass); (c) visible signal arising from LysoTracker Green in channel 2 (*λ*_ex_, 470 nm; *λ*_em_, 530/43 nm bandpass); (d) merged b and c showing colocalization (*P* = 0.52). Scale bar: 10 μm.

The excitation wavelength for the Yb^3+^ complexes could be further extended to the red region upon excitation at the Q band (Fig. S82†). Taking **Yb-4** as an example (Fig. 4), we also observed high NIR intracellular luminescence signals with good co-localization with LysoTracker® Green under 620 nm excitation (∼4 μW cm^–2^). This reveals that the excitation wavelength can be extended from the visible to the red region by biomimetic β-modification.

### NIR time-resolved fluorescence lifetime imaging

Time-resolved fluorescence lifetime imaging (FLIM) has been widely used in the biomedical science field, as it is insensitive to the concentration of the fluorophore, excitation intensity, and photobleaching. This allows visualizing cellular events that are difficult to observe by steady-state fluorescence imaging.^64^,^65^ Although the application of luminescent lanthanides such as Eu^3+^ and Tb^3+^ as visible emitters in FLIM has been extensively demonstrated,^23^,^24^,^66^–^68^ FLIM in the NIR region has still remained unexplored so far. Given the long lifetimes and high luminescence of β-fluorinated Yb^3+^ complexes, they are potential probe candidates for NIR FLIM on the μs scale, which can facilitate discrimination from cell autofluorescence (ns) and enhance signal-to-noise ratios.

**Fig. 5:**
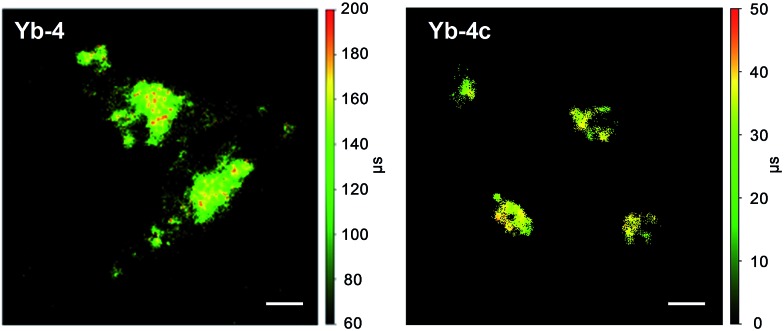
NIR time-resolved images of living HeLa cells incubated with 10 μM **Yb-4** and **Yb-4c** (*λ*_ex_, 408 nm; *λ*_em_, 935/170 nm bandpass; dwell time, 4 ms). Scale bar: 10 μm.

In this work, confocal FLIM experiments were performed using 408 nm pulsed laser excitation (Fig. S83 and 84†) and the details are described in the Experimental section. As shown in Fig. 5, **Yb-4** shows a lifetime distribution between 100 and 200 μs in cells, much longer than that of **Yb-4c** (20–40 μs). More importantly, the lifetime of **Yb-4** varies in different subcellular locations, probably due to the different intracellular microenvironments such as the polarity, viscosity and lipophilicity.

To verify the hypothesis that the NIR decay lifetime of **Yb-4** is sensitive to intracellular microenvironments, we investigated the decay lifetimes of **Yb-4** as an example (Table 2) in various solvents with different viscosities and polarities under air-saturated and degassed conditions.^69^

**Table 2:** Decay lifetime (*τ*_obs_) of **Yb-4** monitored at 980 nm in different solvents (*λ*_ex_ = 410 nm)^*a*^

Solvent	Viscosity (cP)	*E* N T ^*b*^	*τ*_obs_ (μs)	
			Air-equil.	Degassed
Methanol	0.54	0.765	68(2)	74(3)
Ethanol	1.02	0.655	86(4)	99(2)
*t*-Butanol	4.31	0.602	117(4)	127(3)
*i*-Propanol	2.04	0.552	110(5)	120(3)
Glycerol	934	—	192(4)	195(5)

^*a*^Standard error values are given in parentheses; they refer to the reproducibility of the measurements. Experimental relative errors: *τ*_obs_, ±5%.

^*b*^Refers to Reichardt's solvent polarity parameter.^70^

As shown in Table 2, experimental results showed that the degassed solvents slightly prolonged the decay lifetime of Yb^3+^ (*ca.* 10%). The excited state lifetimes in such solvents also exhibited close correlation with the changes of solvent viscosity (Fig. S85†) and polarity (Fig. S86†), even in degassed solvents. Additionally, we further measured the Yb^3+^ lifetimes in water in presence of bovine albumin (BSA) (Table S3 and Fig. S87†), and the Yb^3+^ luminescence intensity and decay lifetime both increased. This may be due to the increased hydrophobic inclusion and decreased solvent quenching effect after protein association,^71^ while quenching of the excited state of the chromophore through the charge transfer process has not been observed.^62^,^63^,^72^ These results revealed that the solvation effect is complicated herein and it seems that not just one factor influences the Yb^3+^ luminescence lifetime. The viscosity, polarity and oxygen concentration may all influence the ground and excited states of the porphyrin and Yb^3+^, and thus vary the Yb^3+^ luminescence lifetime.^69^,^72^–^74^

## Conclusions

Taken together, we reported three synthetic approaches to biocompatible Yb^3+^ complexes, starting from the extremely hydrophobic Yb^3+^ perfluorinated porphyrinates, for NIR living cell imaging. These β-fluorinated Yb^3+^ probes possess the following features: (1) high luminescence (5–13% quantum yields in H_2_O), (2) enhanced stabilities, and (3) long decay lifetimes (>100 μs), which are advantageous for FLIM on the microsecond scale. Combined with the additional advantages such as tunable excitation wavelength (visible to far red region) and large extinction coefficients of porphyrinoid ligands, they are prospective candidates for NIR molecular probes for real-time visualization in clinical diagnoses and surgical operations, which is currently under investigation in our laboratory. This work paves the way for molecular probes for NIR imaging in the range of 900–1100 nm biological window by constructing NIR Ln molecular complexes.

## Experimental section

### General information

Unless otherwise stated, all reactions were performed under an inert atmosphere of nitrogen. UV-vis spectra were recorded on an Agilent 8453 UV-vis spectrometer equipped with an Agilent 89090A thermostat (±0.1 °C) at 25 °C. IR spectra were recorded on a Spectrum Spotlight 200 FT-IR microscope. Mass spectra were recorded on a Bruker APEX IV FT-ICR mass spectrometer (ESI) or AB Sciex MALDI-TOF mass spectrometer. Simulated mass spectra were obtained from the website http://www.chemcalc.org. NMR spectra were recorded on a Bruker ARX400 400 MHz or AVANCE III 500 MHz NMR spectrophotometer. The HeLa cells were obtained from Peking University Health Science Center. For the optical measurements in liquid solution, spectroscopic-grade dimethyl sulfoxide was used as purchased from Alfa-Aesar. Anhydrous 1,2,4-trichlorobenzene (TCB), diisobutyl aluminium hydride (DIBAL-H), sarcosine, and polyformaldehyde were purchased from J&K Scientific and used as received. Anhydrous CH_2_Cl_2_ was distilled from calcium hydride and H_2_O was obtained from Milli-Q Integral.

### Benzaldehyde, Kläui's ligand and porphyrinates

4-(2,3,4,6-Tetraacetyl-glucopyranobseynlz)aldehyde^75^ and *p*-chloromethylbenzaldehyde^76^ were synthesized according to the literature. Other modified-benzaldehydes were all purchased from J&K Scientific and used as received. β-Octafluorinated ytterbium porphyrin (YbF_28_TPP),^48^ β-hexafluorinated ytterbium porpholactone (YbF_26_TPPL),^50^ sodium[(cyclopentadienyl)tris(di(methyl-d_3_)phosphito)cobaltate] (NaL_OCD3_) (D atom >99%)^52^ were synthesized according to literature methods.

### Synthesis of **Yb-1**

**Yb-1** was synthesized from 2,3,7,8,12,13,17,18-octafluoro-5,10,15,20-tetrakis[4-(2,3,4,6-tetraacetyl-glucosyl)-phenyl]porphyrin (**L1**) (Scheme S1†). The synthesis of **L1** was carried out according to procedures in the literature.^75^ 2,6,-Difluoropyrrole (103 mg, 1.0 mmol) and 4-(2,3,4,6-tetraacetyl-glucopyranobseynlz)aldehyde (453 mg, 1.0 mmol) were dissolved in 250 mL methylene chloride. The mixture was stirred for 10 min after which a few drops of BF_3_·Et_2_O were added. The mixture was then stirred overnight at room temperature. 2,3-Dicyano-5,6-dichlorobenzoquinone (DDQ, 0.23 g, 1.0 mmol) was added. After reaction for 1 h, silica gel (2 g) was added to the dark solution and all solvent was evaporated. The absorbed products were placed on the top of a silica gel column. The product was obtained by using ethyl acetate/petroleum ether (v/v = 2 : 1) as the eluent. Then **L1** (200 mg, 0.2 mmol), Yb(acac)_3_·3H_2_O (500 mg, 1 mmol) and 1,2,4-trichlorobenzene (TCB) were added to a Schlenk tube and refluxed overnight. After cooling to room temperature, the reaction mixtures were transferred to a silica column, TCB was first eluted using petroleum ether, and then the unreacted free base ligand was eluted with CH_2_Cl_2_; the corresponding Yb^3+^ complex was obtained by using CH_2_Cl_2_/MeOH (v/v = 5 : 1) as the eluent and used directly in the next step. The obtained Ln complexes (acac as the ancillary ligand) and 3 equiv. NaL_OCD3_ were dissolved in a mixed solvent of CHCl_3_/CH_3_OH (v/v = 1 : 1, 5 mL). The mixture was refluxed for 8 h. After cooling to room temperature, the reaction mixtures were transferred to a silica column, **L1-Yb(iii)-L_OCD3_** (**Yb-L1**) was obtained by using ethyl acetate/petroleum ether (v/v = 4 : 1) as the eluent. Then **Yb-L1** and CH_3_ONa (10 mg) were dissolved in a mixed solvent of CH_2_Cl_2_/CH_3_OH (v/v = 1 : 1, 10 mL) and stirred at room temperature for 3 h. Then the reaction mixtures were transferred to a silica column, the product **Yb-1** was obtained by using CH_3_OH as the eluent.

### Synthesis of **Yb-2**

**Yb-2** was synthesized from 2,3,7,8,12,13,17,18-octafluoro-5-pentafluorophenyl-10,15,20-tri[(methyl-*p*-formyl)-phenyl] porphyrin (**L2**) (Scheme S2†). The synthesis procedures of **L2** and **L2-Yb(iii)-L_OCD3_** (**Yb-L2**) are similar to that described for **L1** and **Yb-L1** but the starting materials are 2,6,-difluoropyrrole (103 mg, 1.0 mmol), pentafluorobenzaldehyde (147 mg, 0.75 mmol) and methyl *p*-formylbenzoate (41 mg, 0.25 mmol). Then **Yb-L2** was dissolved in a mixed solvent of tetrahydrofuran/CH_3_OH (v/v = 1 : 1, 10 mL), with the addition of 1 mL 10% KOH solution. The reaction mixtures were stirred at 50 °C overnight and the solution was neutralized with 0.2 M HCl. After evaporating the organic solvent, the product **Yb-2** was obtained by centrifugation and further recrystallization from CH_3_OH.

### Synthesis of **Yb-3**

**Yb-3** was synthesized from 2,3,7,8,12,13,17,18-octafluoro-5,10,15,20-tetrakis(*p*-chloromethylphenyl) porphyrin (**L3**) (Scheme S3†). The synthesis procedures of **L3** and **L3-Yb(iii)-L_OCD3_** (**Yb-L3**) are similar to that described for **L1** and **Yb-L1** but the starting materials are 2,6,-difluoropyrrole (103 mg, 1.0 mmol) and *p*-chloromethylbenzaldehyde (154 mg, 1.0 mmol). Then **Yb-L3** and excess of triphenylphosphine (50 mg) were dissolved in *N*,*N*-dimethylformamide and refluxed for 24 h. Then the solvent was removed and the product **Yb-3** was obtained by recrystallization from ice cold methanol.

### Synthesis of **Yb-4**

A toluene solution of YbF_28_TPP (50 mg, 0.025 mmol), sarcosine (43 mg, 0.5 mmol), and polyoxymethylene (40 mg) was heated under reflux for 24 h.^49^,^77^ After being cooled to room temperature, all solvent was evaporated. The reaction products were applied on the top of a silica gel column, and **Yb-L4** was obtained by using ethyl acetate/petroleum ether (v/v = 1 : 5) as the eluent. Then **Yb-L4** was dissolved in 10 mL toluene, with the addition of 2 mL CH_3_I. The reaction mixtures were stirred at 50 °C for 48 h, then the product (**Yb-4**) was obtained by filtration and washing with toluene and hexane (Scheme S4†).

### Synthesis of **Yb-5**

YbF_26_TPPL (50 mg, 0.025 mmol) was dissolved in dry CH_2_Cl_2_ (10 mL) and the solution was cooled to 78 °C. DIBAL-H (20% in hexane, 0.2 mL, 7.0 equiv.) was added using a syringe and the reaction mixture was warmed to room temperature and allowed to stir for an additional hour.^78^ The reaction was then quenched by addition of a few drops of H_2_O and evaporated to dryness. The Yb^3+^ porpholactol product **Yb-L5** was obtained by using CH_2_Cl_2_/petroleum ether (v/v = 2 : 1) as the eluent. Then **Yb-L5** and excessive 2-bromoethanol were dissolved in dry CH_2_Cl_2_ (10 mL) and few drops of BF_3_·Et_2_O were added. The reaction solution was allowed to stir for 24 h at room temperature. Then the reaction solution was evaporated and the obtained solid was dissolved in CH_3_CN, with the addition of 1 mL trimethylamine. The reaction solution was refluxed at 90 °C overnight. After cooling to room temperature, the reaction solution was evaporated and the product (**Yb-5**) was obtained by using CH_2_Cl_2_/CH_3_OH (v/v = 5 : 1) as the eluent (Scheme S5†).

### Synthesis of **Yb-2c**

The synthetic procedures for **Yb-2c** were similar to that for **Yb-2**, starting from 5-pentafluorophenyl-10,15,20-tri[(methyl-*p*-formyl)-phenyl] porphyrin (**L2c**). The synthesis procedure of **L2c** and **L2c-Yb(iii)-L_OCD3_** (**Yb-L2c**) are similar to that described for **L2** and **Yb-L2** but the starting materials are pyrrole (67 mg, 1.0 mmol), pentafluorobenzaldehyde (147 mg, 0.75 mmol) and methyl *p*-formylbenzoate (41 mg, 0.25 mmol) (Scheme S6†).

### Synthesis of **Yb-3c**

The synthetic procedures for **Yb-3c** were similar to that for **Yb-3**. The starting 5,10,15,20-tetrakis(*p*-chloromethylpheny1) porphyrin (**L3c**) was synthesized according to literature methods^76^ and the intermediate product **L3c-Yb(iii)-NaL_OCD3_** was synthesized similar to **Yb-L3** (Scheme S7†).

### Synthesis of **Yb-4c**

The synthetic procedures for **Yb-4c** were similar to that for **Yb-4**. The starting porphyrin was synthesized according to literature methods^31^,^41^ and the intermediate product **Yb-L4c** was synthesized similar to **Yb-L4** (Scheme S8†).

### Synthesis of **Yb-5c**

The synthetic procedures for **Yb-5c** were similar to that for **Yb-5**. The starting ytterbium porpholactone was synthesized according to literature methods^50^ and the intermediate product **Yb-L5c** was obtained similar to **Yb-L5** (Scheme S9†).

### Measurement of photophysical properties

Emission, excitation spectra and lifetime were measured on an Edinburgh Analytical Instruments FLS980 lifetime and steady state spectrometer equipped with a 450 W Xe lamp, a 60 W microsecond flash lamp, PMT R928 for the visible emission spectrum, HAMAMATSU R5509-73 PMT with a C9940-02 cooler for the NIR emission spectrum and luminescence lifetime. Excitation and emission spectra were corrected for instrumental functions (including the correction for detector, gratings *etc.*). All luminescence decays were exponentially tail-fitted by monoexponential functions without deconvolution for the negligible instrumental reference function in the NIR region.

### Quantum yield determination

Quantum yields in solution were determined using a comparative method and the equation: *Φ*_s_/*Φ*_r_ = (*G*_s_/*G*_r_) × (*η*_s_^2^/*η*_r_^2^), where the subscripts r and s denote the reference and sample respectively, *Φ* is the quantum yield, *G* is the slope from the plot of integrated emission intensity *vs.* absorbance, and *η* is the refractive index of the solvent.^79^ The reference was YbTPP(L_OEt_) in CH_2_Cl_2_ (*Φ*_Δ_ = 0.024).^53^ Generally, YbTPP(L_OEt_) and the solution of Yb^3+^ complex with 4 different concentrations were firstly prepared in the corresponding solvents. The absorbance of all the solutions at 410 nm was recorded on an Agilent 8453 UV-vis spectrometer equipped with an Agilent 89090A thermostat (±0.1 °C) and NIR emissions were recorded on an Edinburgh Analytical Instruments FLS980 lifetime and steady state spectrometer with the excitation wavelength at 410 nm under identical conditions. According to the ratio of the slope *G*_Yb – *x*_/*G*_r_ (*x* = 1–5, **2c–5c**), the relative quantum yield of Yb^3+^ complexes could be obtained according to the equation described above. The absorbance values of all the samples and references are below 0.1 and the absorbance values undergo background correction by subtracting the average over a range from 800 to 820 nm. The integrated emission intensity integrated from 880 nm to 1150 nm was obtained by subtracting the blank (integrated emission intensity with only the pure solvent under identical conditions). The estimated error for the quantum yield measurements is 15%. For the quantum yield measured in degassed solution, solutions of Yb^3+^ complexes with 4 different concentrations were degassed *via* 5 freeze–pump–thaw cycles. Then the measurement and calculation were the same as above.

### Singlet oxygen quantum yield measurement

Measurements were taken at 410 nm excitation in air-saturated solutions at room temperature with TPP (*Φ*_Δ_ = 0.55) in CHCl_3_ as the reference (ref. 59). The absorption maximum of the sensitizer at the corresponding wavelength was generally kept below 0.2.

### Determination of the octanol–water partition coefficients (log *P*)

Equal volumes (2000 mL) of *n*-octanol and water were thoroughly mixed using an oscillator and separated after 24 h. Yb^3+^ complex (0.1 mg each) was then dissolved in 40 mL of the separated *n*-octanol and the solution and 40 mL of water (previously separated from the mixture) was added. The new water–octanol system was allowed to equilibrate for additional 24 h. After separation, both fractions were analyzed by UV-vis spectra. The log *P* values were calculated with the following equation:^80^

1 log *P* = log(*C*_octanol_/*C*_water_)

where *C*_octanol_ and *C*_water_ refer to the concentrations of Yb^3+^ complexes in *n*-octanol and water, respectively.

### Cell culture

All HeLa cells were incubated in complete medium (Dulbecco's modified Eagle's Medium, supplemented with 10% fetal bovine serum (FBS) and 1% penicillin–streptomycin) at 37 °C in an atmosphere containing 5% CO_2_.

### Dark cytotoxicity assay

HeLa cells were seeded in flat-bottomed 96-well plates, 10^4^ cells per well, with 200 μL complete culture media in the dark for 24 h. Cells were incubated with 20 μM complexes for another 24 h in the dark while wells containing no cells are set as the controls. After washing three times with PBS, 10 μL Cell Counting Kit-8 (CCK-8) solution and 90 μL PBS were added per well. After 2 hours, the absorbance at 450 nm was read using a 96-well plate reader. The viability of HeLa cells was calculated by the following equation:

2 CV = (*A*_s_ – *A*_b_)/(*A*_c_ – *A*_b_) × 100%

CV stands for the viability of cells, *A*_s_, *A*_c_ and *A*_b_ stand for the absorbance of cells containing the studied complexes, cell control (no treated cells) and blank control (wells containing neither cells nor the studied complexes).

### Light-induced cytotoxicity assay

HeLa cells were seeded in flat-bottomed 96-well plates, 10^4^ cells per well, with 200 μL complete culture media in the dark for 24 h. Cells were incubated with varying concentrations from 0 to 16 μM complexes for another 24 h in the dark while wells containing no cells are set as the controls. After washing three times with PBS, the cells were irradiated for 30 min in 100 μL PBS under the light irradiation (400–700 nm) with the same dose of light (6.5 mW cm^–2^) for 30 min. Then PBS was replaced by 200 μL fresh culture media. After being cultured for 24 h, the cells were washed three times with PBS. Then 10 μL Cell Counting Kit-8 (CCK-8) solution and 90 μL PBS were added per well. After 2 hours, the absorbance at 450 nm was read using a 96-well plate reader. The viability of HeLa cells was calculated by using eqn (2) (see above).

### Inductively coupled plasma (ICP) spectrometry experiments for cellular uptake quantification

To quantify the concentration of Yb^3+^ in cells, 1 × 10^6^ cells were seeded in a six-well microplate. After 36 h of attachment, the cells were incubated with 10 μM of corresponding Yb^3+^ complexes for 12 h at 37 °C. Cells were trypsinized and resuspended in nitric acid overnight. Then the solution was heated at 100 °C for 4 h before adding water to achieve a final volume of 10 mL. The measurements were taken on an inductively coupled plasma-atomic emission spectrometer (Prodigy 7, Leeman).

### Colocalization assay

HeLa cells were placed onto 0.1 mM poly-d-lysine coated glasses in complete media and the cells were incubated for 24 h. A stock solution of Yb^3+^ complex in chromatographic grade, anhydrous DMSO was prepared as 2 mM. The solution was diluted to a final concentration of 10 μM using complete growth medium. Stock solutions of Lyso Tracker Green DND-26 were prepared as 1 mM, and the stock solution was diluted to the working concentrations in complete medium (Lyso Tracker: 75 nM). After incubation of 10 μM Yb^3+^ complex for 12 h followed by 30 min incubation with 75 nM solution of LysoTracker Green, cells were washed with PBS buffer twice before confocal experiments.

### NIR confocal images

The ISS Alba*5* FLIM/FFS confocal system (ISS Inc.) was used to acquire the confocal images. The system was attached to a Nikon TE2000 inverted microscope, equipped with the Nikon 60X/1.2 NA water immersion objective lens. Both steady-state and time-resolved (lifetime) confocal images were acquired using the ISS VistaVision software and the ISS FastFLIM data acquisition unit. A 408 nm diode laser or YSL supercontinuum laser source (for excitation at the Q band) was used for the excitation of Yb^3+^ dyes; the laser was operated in the CW mode for the steady-state imaging and modulated On/Off by FastFLIM for lifetime imaging; the average power on the specimen plane was about 4 μW cm^–2^. For lifetime imaging, the On/Off repetition rate and the duty cycle were adjusted using the VistaVision software to optimize the decay window based on the measured lifetime, *e.g.* 1 KHz and 1% duty cycle for **Yb-1–5**; due to the long lifetime, a long scanning dwell time of 4 ms was used. For colocalization studies, a 470 nm diode laser operated in the CW mode was used for the excitation of LysoTracker Green. The Yb^3+^ confocal images were acquired in Channel 1, using the Semrock 935/170 nm bandpass filter (EM1) and the single photon avalanche photodiode detector (SPAD) from Excelitas (model SPCM-AQRH-15, quantum efficiency >25% at 950 nm). The LysoTracker Green confocal images were collected in Channel 2, using the Semrock 530/43 nm bandpass filter (EM2) and the GaAsP photomultiplier tube detector from Hamamatsu (Model H7422p-40). A 405/470/561/685 nm multi-edge dichroic beamsplitter (D2) from Chroma was used to separate the two excitation wavelengths (samples and lysosome tracker) from the corresponding emission wavelengths. A 650 nm longpass dichroic beamsplitter (D3) from Chroma was used to separate the emission light between Channel 1 and Channel 2. A variable pinhole (VP) was used for each imaging channel and was set to be 1 Airy Unit for the confocal imaging. All fluorescence images were processed and analyzed using ImageJ.

## Conflicts of interest

There are no conflicts to declare.

## Supplementary Material

Supplementary information
