# Supplementary material for: Highly luminescent, biocompatible ytterbium(iii) complexes as near-infrared fluorophores for living cell imaging
Source: Chem Sci. 2018 Mar 19;9(15):3742–53. doi: 10.1039/c8sc00259b (PMC5939605; doi:10.1039/c8sc00259b)
Supplement: Supplementary file 1 [file SC-009-C8SC00259B-s001.pdf]

Electronic Supplementary Material (ESI) for Chemical Science.
This journal is © The Royal Society of Chemistry 2018

Electronic Supplementary Information

Highly Luminescent, Biocompatible Ytterbium(III) Complexes as Near-Infrared Fluorophores for Living Cell Imaging

Yingying Ning,[a] Juan Tang,[a] Yi-Wei Liu,[a] Jing Jing,*[,b] Yuan-Sheng Sun,[c] and Jun-Long Zhang*[,a]

[a] Beijing National Laboratory for Molecular Sciences, State Key Laboratory of Rare Earth Materials Chemistry and Applications, College of Chemistry and Molecular Engineering, Peking University, Beijing 100871, P. R. China

[b] School of Chemistry, Beijing Institute of Technology, Beijing 100081, P. R. China

[c] ISS Inc, Champaign, IL 61822, USA

Supporting information for synthesis and characterization.................................. 4
Synthesis of **Yb-1**..4
Synthesis of **Yb-2**..5
Synthesis of **Yb-3**..6
Synthesis of **Yb-4**..7
Synthesis of **Yb-5**..8
Synthesis of **Yb-2c**...9
Synthesis of **Yb-3c**...10
Synthesis of **Yb-4c**...11
Synthesis of **Yb-5c**...12
Table S1. Lipophilicity (log $P_{o/w}$) of **Yb-1-5** and **Yb-2c-5c**. 13
Table S2. Determination of cellular uptake in HeLa cells by ICP. 14
Table S3. Decay lifetime (τ_{obs}) monitored at 980 nm of **Yb-1-5** in water and 0.2 M bovine albumin (BSA) under air-saturated condition.[a,b]..15
Figs. S1-S22. ^{1}H NMR spectra 16
Figs. S23-S42. ^{19}F NMR spectra...38
Figs. S43-S51. MS spectra..58
Figs. S52-S60. IR spectra. 67

Supporting information for photophysical property measurements.................. 76
Fig. S61 Normalized absorption spectra of **Yb-2c-5c** in H_2O at room temperature (with 0.1% DMSO)..76
Fig. S62 Normalized excitation and emission spectra of **Yb-1-5** in DMSO at room temperature. 77
Fig. S63 Normalized excitation and emission spectra of **Yb-2c-5c** in water at room temperature.78
Fig. S64 Integrated emission intensity vs absorbance plots for relative quantum yield determination of **Yb-1-5** vs YbTPP(LOEt) (λ_{ex} = 410 nm, Φ_r = 0.024) in DMSO and H_2O at room temperature. 79
Fig. S65 Integrated emission intensity vs absorbance plots for relative quantum yield determination of **Yb-2c-5c** vs YbTPP(LOEt) (λ_{ex} = 410 nm, Φ_r = 0.024) in DMSO and H_2O at room temperature. 80
Fig. S66 NIR luminescence decay curve monitored at 980 nm **Yb-1-5** in DMSO and H_2O at room temperature (λ_{ex} = 410 nm, $OD_{410\,nm}$ = 0.1). 81
Fig. S67 NIR luminescence decay curve monitored at 980 nm **Yb-2c-5c** in DMSO and H_2O at room temperature (λ_{ex} = 410 nm, $OD_{410\,nm}$ = 0.1). 82
Fig. S68 Integrated emission intensity vs absorbance plots for relative quantum yield determination of **Yb-1-5** vs YbTPP(LOEt) (λ_{ex} = 410 nm, Φ_r = 0.024) in degassed DMSO and H_2O at room temperature. 83
Fig. S69 Integrated emission intensity vs absorbance plots for relative quantum yield determination of **Yb-2c-5c** vs YbTPP(LOEt) (λ_{ex} = 410 nm, Φ_r = 0.024) in degassed DMSO and H_2O at room temperature. 84
Fig. S70 NIR luminescence decay curve monitored at 980 nm **Yb-1-5** in degassed DMSO and H_2O at room temperature (λ_{ex} = 410 nm, $OD_{410\,nm}$ = 0.1). 85
Fig. S71 NIR luminescence decay curve monitored at 980 nm **Yb-2c-5c** in degassed DMSO and H_2O at room temperature (λ_{ex} = 410 nm, $OD_{410\,nm}$ = 0.1). 86

Fig. S72 Normalized emission intensity of **Yb-4** in DMSO at room temperature and MeOH/EtOH (v/v = 1/1) at 77 K.......87
Fig. S73 Stabilities of **Yb-1-5** in DMSO.......88
Fig. S74 Stabilities of **Yb-1-5** in H_2O with 1% DMSO.......89
Fig. S75 ^{2}D NMR (77 MHz) of **Yb-4** in CH_3OH.......90

Supporting information for singlet oxygen quantum yield determination and cell experiments....... 91
Fig. S76 Dark cytotoxicity of 20 µM **Yb-1-5** toward Hela cells.......91
Fig. S77 Photocytotoxicity of **Yb-1-5** toward Hela cells using CCK-8 assay under the light irradiation (400–700 nm, 6.5 mW/cm^2) for 30 min, and then cultured in the dark for another 24 hours.......92
Fig. S78 a) NIR emission of $^1O_2$ at 1270 nm excited at 410 nm in the $CHCl_3$ solution of **Yb-1-5**; b) Relative intensity of singlet oxygen production vs absorption for **Yb-5** and TPP at 410 nm.......93
Fig. S79 Experimental setup for NIR live cell imaging.......94
Fig. S80 NIR images of **Yb-1** in HeLa cells (λ_{ex} = 408 nm).......95
Fig. S81 NIR images of **Yb-2c** and **Yb-3c** in HeLa cells (λ_{ex} = 408 nm).......96
Fig. S82 NIR images of **Yb-2** and **Yb-3** in HeLa cells (λ_{ex} = 600 nm).......97
Fig. S83 NIR time-resolved image of **Yb-2** and **Yb-2c** in HeLa cells (λ_{ex} = 408 nm).......98
Fig. S84 NIR time-resolved image of **Yb-3** and **Yb-3c** in HeLa cells (λ_{ex} = 408 nm).......99
Fig. S85 The decay lifetimes of **Yb-4** monitored at 980 nm relative to the solvent viscosity.......100
Fig. S86 The decay lifetimes of **Yb-4** monitored at 980 nm relative to Reichardt's normalised solvent polarity parameter.......101
Fig. S87 Emission Intensity of **Yb-1-5** in water and 0.2 M bovine albumin (BSA) (λ_{ex} = 410 nm, $A_{410\ nm}$ = 0.1).......102

Supporting information for synthesis and characterization.

Synthesis of Yb-1.

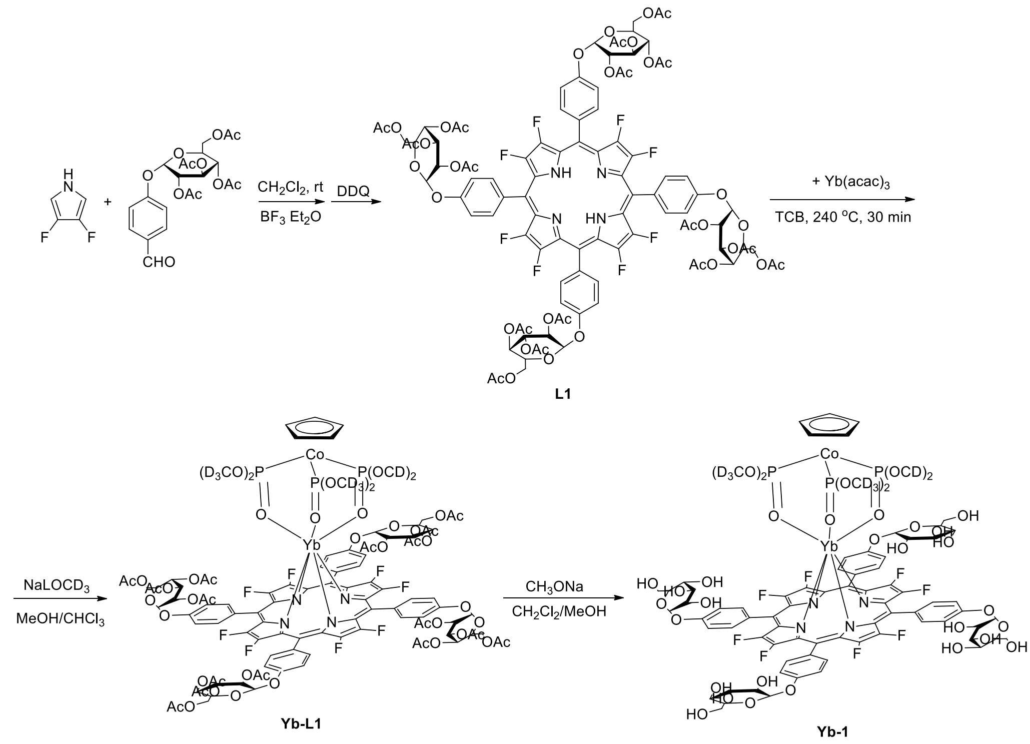

Scheme S1. Synthetic procedure of **Yb-1**.

Complex **L1**: Yield: 32%; ^{1}H NMR (400 MHz, $CDCl_3$) δ -4.15 (s, 2H), 2.21-2.20 (m, 48H), 4.29 (m, 4H), 4.30 (m, 4H), 4.39 (m, 4H), 5.32 (m, 4H), 5.44 (m, 12H), 7.36 (d, 8H), 7.98 (d, 8H). ^{19}F NMR (377 MHz, $CDCl_3$) δ -140.30 (s, 4F), -145.27 (s, 4F). UV/Vis (CH_2Cl_2, 25 °C): λ_{maxz}(nm) (log ε): 408 (5.54), 501 (4.32), 539 (4.22), 582 (4.20), 569 (4.15).

Complex **Yb-L1**: Yield: 5%; ^{1}H NMR (400 MHz, $CDCl_3$) δ 16.48 (s, 4H), 10.20 (s, 4H), 9.31 (s, 4H), 8.74 (s, 4H), 6.75 (d, 4H), 6.31 (t, 4H), 6.08 (t, 4H), 5.90 (t, 4H), 5.01 (m, 4H), 4.94 (m, 4H), 4.82 (m, 4H), 2.87 (s, 12H), 2.59 (s, 12H) 2.43 (s, 12H), 2.36 (s, 12H), -5.18 (s, 5H). ^{19}F NMR (377 MHz, $CDCl_3$) δ -132.32 (d, 8F). UV/Vis (CH_2Cl_2, 25 °C): λ_{max}(nm) (log ε): 420 (5.55), 548 (4.41), 593 (4.43).

Complex **Yb-1**: Yield: 95%; ^{1}H NMR (400 MHz, $CDCl_3$) δ -5.75 (s, 5H), 4.13 (t, 4H), 4.30 (t, 4H), 4.45 (m, 8H), 4.50 (t, 4H), 4.65 (t, 4H), 6.64 (s, 4H), 8.64 (s, 24H), 9.01 (s, 4H), 9.71 (s, 4H), 10.27 (s, 4H), 16.10 (s, 4H). ^{19}F NMR (377 MHz, $CDCl_3$) δ -134.65 (s, 8F). HR-MS (ESI^+) *m/z* $[M+H]^+$: Calcd for $C_{79}H_{66}D_{18}CoF_8N_4O_{33}P_3Yb$ 2112.3953; found: 2112.3885. UV/Vis (DMSO, 25 °C): λ_{max}(nm) (log ε): 423 (5.49), 548 (4.32), 597 (4.35).

Synthesis of Yb-2.

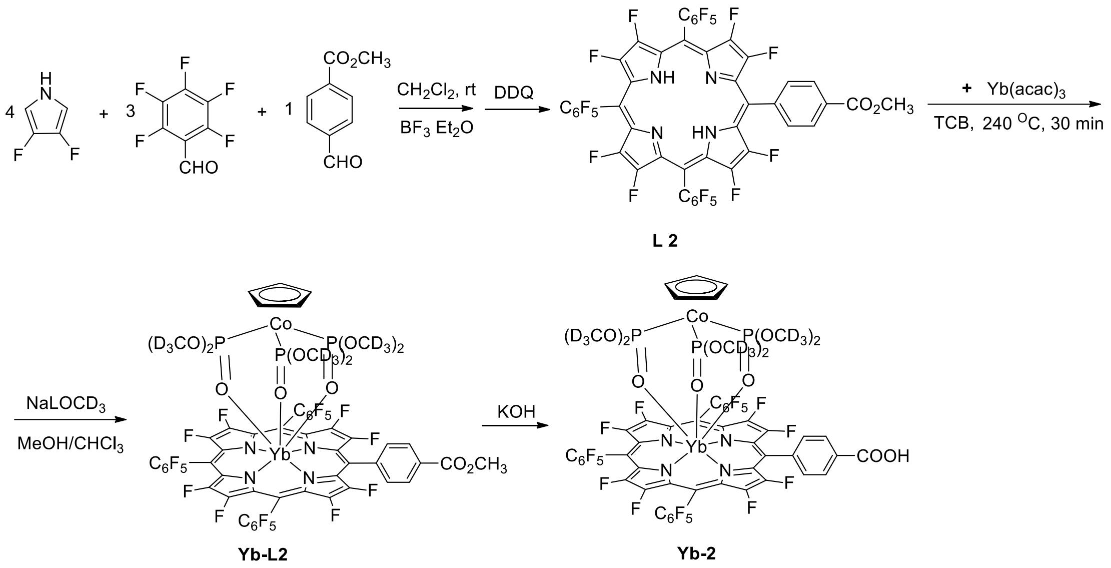

Scheme S2. Synthetic procedure of **Yb-2**.

Complex **L2**: Yield: 10%; ^{1}H NMR (400 MHz, $CDCl_3$) δ -4.20 (s, 2H), 4.10 (s, 3H), 8.14 (d, 2H), 8.46 (d, 2H). ^{19}F NMR (377 MHz, $CDCl_3$) δ -136.96 (s, 1F), -138.60 (m, 2F), -138.74 (m, 4F), -141.79-148.65 (m, 7F), -150.22 (m, 3F), -161.52 (m, 6F). UV/Vis (CH_2Cl_2, 25 °C): λ_{max}(nm) (log ε): 395 (5.62), 494 (4.45), 536 (4.38), 578 (4.32).

Complex **Yb-L2**: Yield: 70%; ^{1}H NMR (400 MHz, $CDCl_3$) δ -5.53 (s, 5H), 4.93 (s, 3H), 9.08 (s, 2H), 9.75 (s, 1H), 11.74 (s, 1H), 18.36 (s, 1H). ^{19}F NMR (471 MHz, CDC13) δ -129.75 (d, J = 30.8 Hz, 2F), -134.51 (m, 4F), -139.34 (m, 5F), -156.39 – -159.33 (m, 10F), -159.94 (s, 2F). UV/Vis (CH_2Cl_2, 25 °C): λ_{max}(nm) (log ε): 414 (5.51), 544 (4.60), 580 (4.08).

Complex **Yb-2**: Yield: 91%; ^{1}H NMR (400 MHz, $CDCl_3$) δ -5.48 (s, 5H), 8.08 (s, 1H), 9.47 (s, 1H), 9.92(s, 1H), 11.80(s, 1H), 18.41(s, 1H). ^{19}F NMR (377 MHz, $CDCl_3$) δ -128.70 (s, 2F), -130.53 (d, J = 23.1, 2F), -131.02 (d, J = 21.7, F), -136.75 (s, 2F), -137.12 (s, 2F), -137.68 (s, 2F), -139.35 (t, 2F), -150.21 (m, 3F), -159.96 (m, 3F), -161.18 (m, 3F). MALDI-TOF m/z $[M+H_2O]^+$: Calcd for $C_{56}H_{14}D_{18}CoF_{23}N_4O_{13}P_3Yb$ 1749.0662; found: 1748.62; UV/Vis (DMSO, 25 °C): λ_{max}(nm) (log ε): 420 (5.52), 548 (4.58), 592 (4.02).

Synthesis of Yb-3.

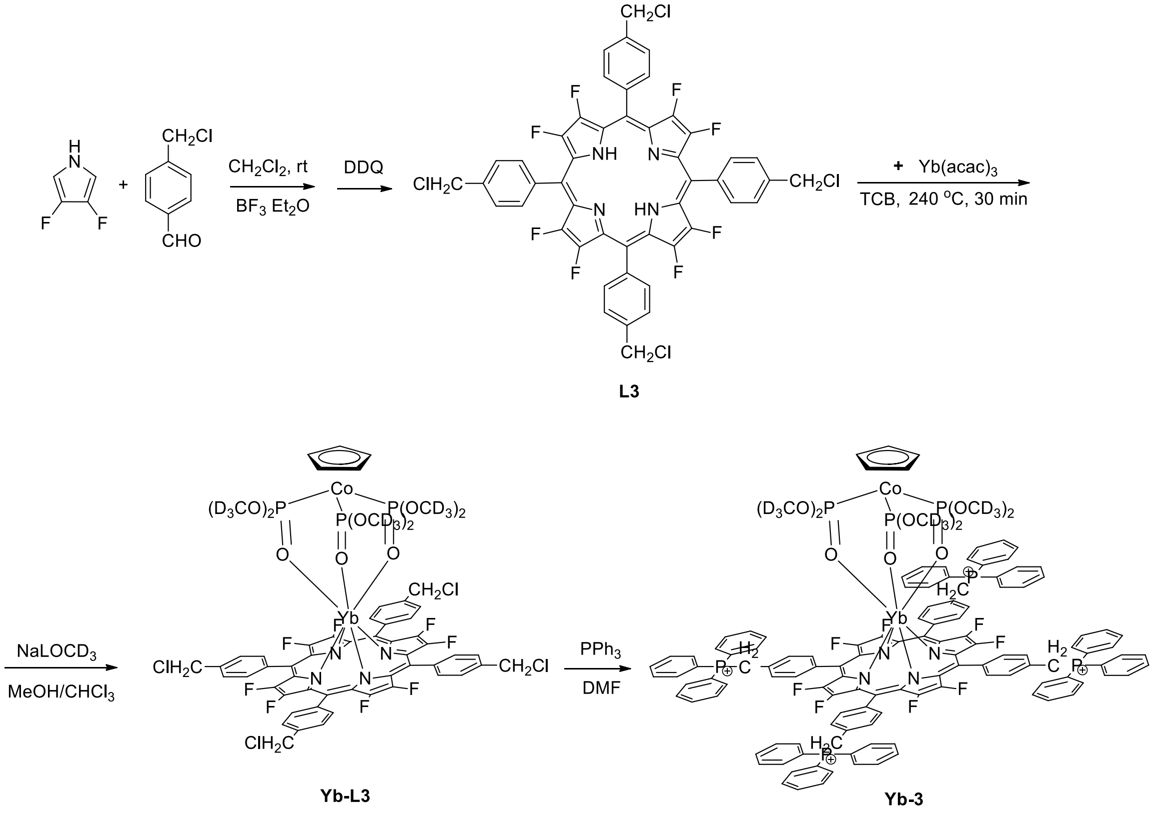

Scheme S3. Synthetic procedure of **Yb-3**.

Complex **L3**: Yield: 55%; ^{1}H NMR (400 MHz, $CDCl_3$) δ -4.15 (s, 2H), 4.91 (s, 8H), 7.76 (d, 8H), 8.02 (d, 8H). ^{19}F NMR (377 MHz, $CDCl_3$) δ -140.20 (s, 4F), -145.29 (s, 4F). UV/Vis (CH_2Cl_2, 25 °C): λ_{max}(nm) (log ε): 403 (5.71), 499 (4.55), 503 (4.50), 582 (4.51), 636 (4.47).

Complex **Yb-L3**: Yield: 12%; ^{1}H NMR (400 MHz, $CDCl_3$) δ -5.16 (s, 5H), 6.29 (d, 8H), 9.01 (s, 8H), 10.72 (s, 4H), 16.95 (s, 4H). ^{19}F NMR (377 MHz, $CDCl_3$) δ -132.00 (s, 8F). UV/Vis (CH_2Cl_2, 25 °C): λ_{max} (log ε): 415 (5.55), 543 (4.41), 586 (4.28).

Complex **Yb-3**: Yield: 85%; ^{1}H NMR (400 MHz, CD_3OD) δ -5.47 (s, 5H), 6.71 (d, 8H), 7.47 (m, 12H), 7.61 (m, 12H), 8.45 (m, 12H), 8.56(m, 16H), 8.97 (m, 24H). ^{19}F NMR (377 MHz, CD_3OD) δ -133.83 (s, 8F). HR-MS (ESI^+) *m/z* $[M]^{3+}$: Calcd for $C_{131}H_{89}D_{18}CoF_8N_4O_9P_7Yb^{3+}$ 833.1967; found: 833.1961; UV/Vis (DMSO, 25 °C): λ_{max}(nm) (log ε): 416 (5.35), 545 (4.22), 588 (4.09).

Synthesis of Yb-4.

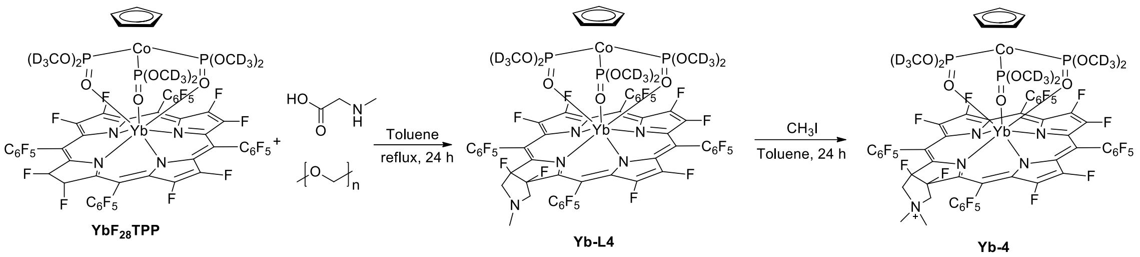

Scheme S4. Synthetic procedure of **Yb-4**.

Complex **Yb-L4**: Yield: 35%; ^{1}H NMR (400 MHz, $CDCl_3$) δ -5.41 (s, 5H), 5.86 (s, 3H), 11.66 (s, 2H), 13.11 (s, 2H). ^{19}F NMR (471 MHz, $CDCl_3$) δ -122.56 (s, 2F), -125.74 (d, *J* = 20.4 Hz, 2F), -127.81 (s, 2F), -135.73 (d, *J* = 24.2 Hz, 2F), -137.71 (s, 2F), -143.04 (s, 2F), -145.00 (d, *J* = 19.3 Hz, 2F), -146.06 (s, 2F), -149.16 (t, *J* = 19.8 Hz, 2F), -150.72 (t, *J* = 20.4 Hz, 2F), -158.73 (t, *J* = 19.0 Hz, 2F), -158.97 (t, *J* = 18.2 Hz, 2F), -161.13 (t, *J* = 21.3 Hz, 2F), -162.87 (t, *J* = 21.4 Hz, 2F). UV/Vis (CH_2Cl_2, 25 °C): λ_{max}(nm) (log ε): 405 (5.50), 509 (4.17), 569 (4.30), 604 (5.14).

Complex **Yb-4**: Yield: 92%; ^{1}H NMR (400 MHz, CD_2Cl_2) δ -5.58 (s, 5H), 7.51 (s, 3H), 12.19 (s, 3H), 14.07 (s, 2H), 15.69 (s, 2H). ^{19}F NMR (471 MHz, CD_2Cl_2) δ -118.05 (s, 2F), -123.57 (s, 2F), -125.14 (s, 2F), -136.67 (s, 2F), -137.77 (s, 2F), -142.61 (s, 2F), -145.28 (s, 2F), -145.75 (s, 2F), -146.85 (s, 2F), -151.38 (s, 2F), -156.06 (s, 2F), -158.29 (s, 2F), -159.25 (s, 2F), -164.13 (s, 2F). HR-MS (ESI^{+}) *m/z* $[M]^{+}$: Calcd for $C_{59}H_{15}D_{18}CoF_{28}N_5O_9P_3Yb$ 1831.0889; found: 1831.0938. UV/Vis (DMSO, 25 °C): λ_{max}(nm) (log ε): 406 (5.44), 510 (4.09), 573 (4.22), 601 (5.06).

Synthesis of Yb-5.

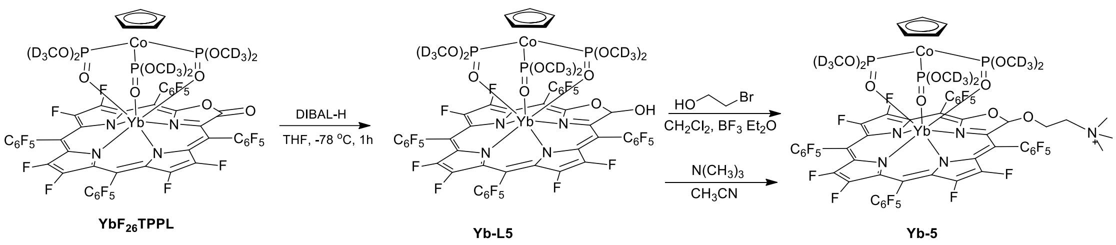

Scheme S5. Synthetic procedure of **Yb-5**.

Complex **Yb-L5**: Yield: 75%; ^{1}H NMR (400 MHz, $CDCl_3$) δ-5.43 (s, 5H), 21.47 (s, 1H), 38.21 (s, 1H). ^{19}F NMR (471 MHz, $CDCl_3$) δ -129.16 (d, *J* = 22.8 Hz, 1F), -130.29 (s, 1F), -131.84 (d, *J* = 21.7 Hz, 1F), -132.31 (s, 1F) , -133.65 (d, *J* = 26.2 Hz, 1F), -134.41 (d, *J* = 21.6 Hz, 1F), -137.18 (d, *J* = 24.5 Hz, 1F), -137.51 (d, *J* = 27.7 Hz, 1F), -138.33 (dd, *J* = 48.7, 21.6 Hz, 2F), -139.07 (s, 1F), -140.92 (s, 1F) , -148.02 (s, 1F) , -149.88 (t, *J* = 20.4 Hz, 1F), -150.38 – -150.95 (m, 3F), -151.50 (t, *J* = 20.5 Hz, 1F), -158.87 (t, *J* = 18.4 Hz, 1F), -159.57 (t, *J* = 21.6 Hz, 1F), -160.17 (t, *J* = 19.7 Hz, 1F), -160.48 – -161.35 (m, 5F). UV/Vis (CH_2Cl_2, 25 °C): λ_{max}(nm) (log ε): 406 (5.58), 510 (5.10), 573 (4.30), 616 (5.23).

Complex **Yb-5**: Yield: 55%; ^{1}H NMR (400 MHz, $CDCl_3$) δ -5.59 (s, 5H), 3.32 (s, 9H), .5.27 (s, 1H), 5.63 (s, 1H), 10.03 (s, 1H), 10.68 (s, 1H), 27.04 (s, 1H). ^{19}F NMR (471 MHz, $CDCl_3$) δ -128.98 (d, *J* = 22.2, 1F), 129.56 (t, 2F), -129.91 (d, *J* = 20.8, 1F) , -135.23 (s, 1F), -135.43 (d, *J* = 20.5, 1F), -138.82 (d, *J* = 19.2, 2F), -139.09 (s, 1F), -139.21 (d, *J* = 20.1, 1F), -142.27 (d, *J* = 21.2, 1F), -143.45 (d, *J* = 19.8, 1F), -145.44 (s, 1F), -147.07 (m, 1F), -147.71 (s, 1F), -148.83 (m, 1F), -150.61 (t, 1F), -151.00 (t, 1F), -156.13 (t, 1F), -157.97 (t, 1F), -159.57 (t, 1F), -159.84 (t, 1F), -160.77 (t, 1F), -161.02 (t, 1F), -162.06 (t, 1F), -162.62 (t, 1F). HR-MS (ESI^{+}) *m/z* [M]$^{+}$: Calcd for $C_{59}H_{19}D_{18}CoF_{26}N_5O_{11}P_3Yb$ 1829.1138; found: 1829.1132. UV/Vis (DMSO, 25 °C): λ_{max}(nm) (log ε): 410 (5.65), 511 (4.12), 574 (4.31), 618 (5.23).

Synthesis of Yb-2c.

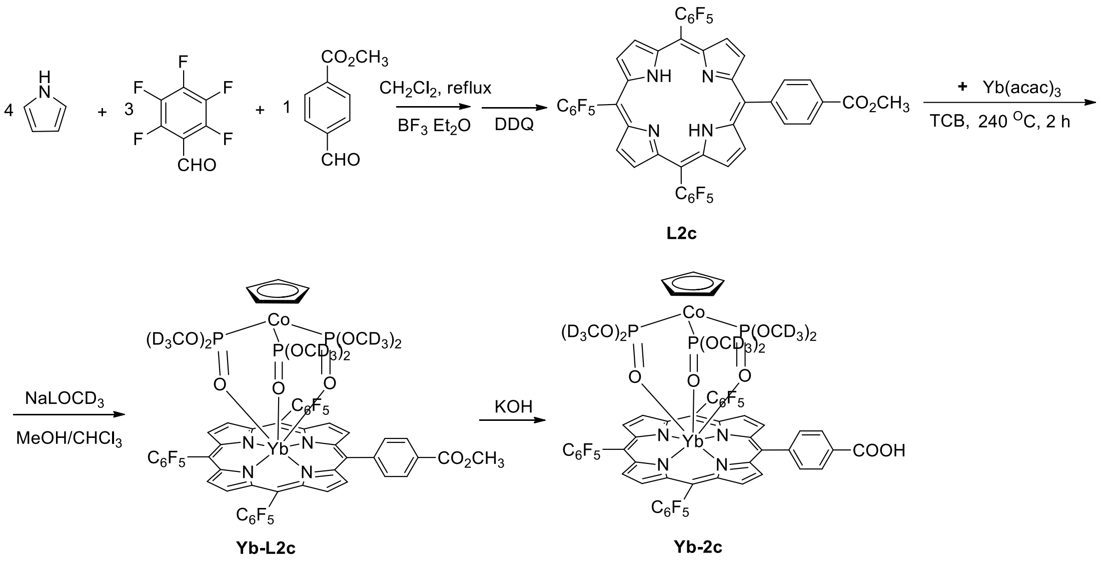

Scheme S6. Synthetic procedure of **Yb-2c**.

Complex **L2c**: Yield: 40%; ^{1}H NMR (400 MHz, $CDCl_3$) δ-2.86 (s, 2H), 4.13 (s, 3H), 8.32 (d, 2H), 8.47, 8.85-8.91 (m, 8H). ^{19}F NMR (471 MHz, $CDCl_3$) δ -136.54 (dd, *J* = 23.6, 8.1 Hz, 2F)), -136.68 (dd, *J* = 23.7, 8.0 Hz, 4F), -151.44 – -151.85 (m, 3F), -161.58 (dtd, *J* = 29.0, 23.6, 7.9 Hz, 6F). UV/Vis (CH_2Cl_2, 25 °C): λ_{max}(nm) (log ε): 413 (5.45), 508 (4.51), 543 (4.42), 583 (4.40).

Complex **Yb-L2c**: Yield: 55%; ^{1}H NMR (400 MHz, $CDCl_3$) δ -5.50 (s, 5H), 4.96 (s, 3H), 9.13 (s, 1H), 9.78 (s, 1H), 11.77 (s, 1H), 18.41(s, 1H). ^{19}F NMR (471 MHz, $CDCl_3$) δ -129.12 (s, 1F) , -131.11 (dd, *J* = 222.7, 21.4 Hz, 2F), -137.36 (d, *J* = 160.0 Hz, 3F), -138.12 (s, 1F), -139.39 (dd, *J* = 39.8, 22.2 Hz, 2F), -150.34 (dt, *J* = 61.9, 20.1 Hz, 2F), -160.13 (dt, *J* = 73.0, 18.7 Hz, 2F), -161.35 (q, *J* = 22.3, 21.4 Hz, 2F). UV/Vis (CH_2Cl_2, 25 °C): λ_{max}(nm) (log ε): 424 (5.60), 553 (4.43), 588 (4.05).

Complex **Yb-2c**: Yield: 91%; ^{1}H NMR (400 MHz, $CDCl_3$) δ -4.76 (s, 5H), 9.10 (s, 1H), 9.79 (s, 1H), 11.65 (s, 1H), 14.61 (s, 2H), 14.83 (s, 2H), 15.23 (s, 2H), 15.64 (s, 2H), 17.58 (s, 1H). ^{19}F NMR (377 MHz, $CDCl_3$) δ -130.67 (d, *J* = 23.5, 4F), -138.45 (d, *J* = 22.7 2F), -138.88 (d, *J* = 23.3, 1F), -156.66 (t, 4F), -157.79 (m, 4F). MALDI-TOF *m/z* $[M+2H_2O]^+$: Calcd for $C_{56}H_{22}D_{18}CoF_{15}N_4O_{13}P_3Yb$ 1605.1416; Found: 1605.1946; UV/Vis (DMSO, 25 °C): λ_{max}(nm) (log ε): 425 (5.56), 533 (4.41).592 (4.03).

Synthesis of Yb-3c.

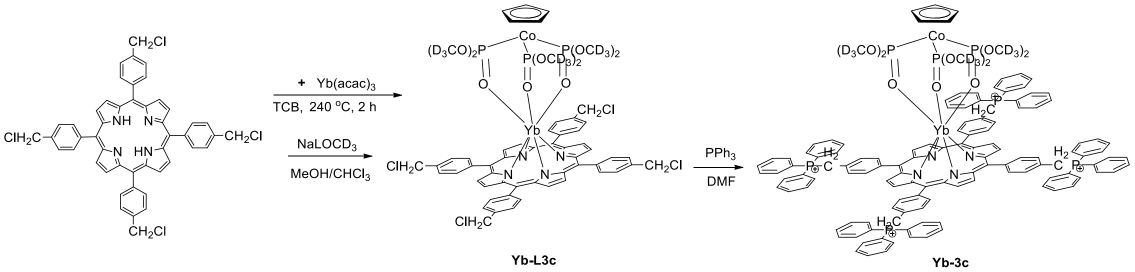

Scheme S7. Synthetic procedure of **Yb-3c**.

Complex **Yb-L3c**: Yield: 13%; ^{1}H NMR (400 MHz, $CDCl_3$) δ -4.50 (s, 5H), 6.19 (s, 8H), 8.63 (s, 4H), 8.77 (s, 4H), 10.59 (s, 4H), 15.21 (s, 4H), 16.75 (s, 4H). UV/Vis (CH_2Cl_2, 25 °C): λ_{max}(nm) (log ε): 428 (5.52), 558 (4.60), 598 (4.20).

Complex **Yb-3c**: Yield: 92%; ^{1}H NMR (400 MHz, $CDCl_3$) δ -4.78 (s, 5H), 7.40 (m, 16H), 7.48-7.61 (m, 8H), 8.25 (m, 12H), 8.35 (m, 16H), 8.80(m, 24H), 10.34 (s, 4H), 15.04 (s, 8H), 16.55 (s, 4H). HR-MS (ESI^+) *m/z* $[M]^{4+}$: Calcd for $[C_{131}H_{97}D_{18}CoN_4O_9P_7Yb]^{4+}$ 588.9170; found: 588.9154; UV/Vis (DMSO, 25 °C): λ_{max} (nm) (log ε):430 (5.59), 561 (4.61), 599 (4.22).

Synthesis of Yb-4c.

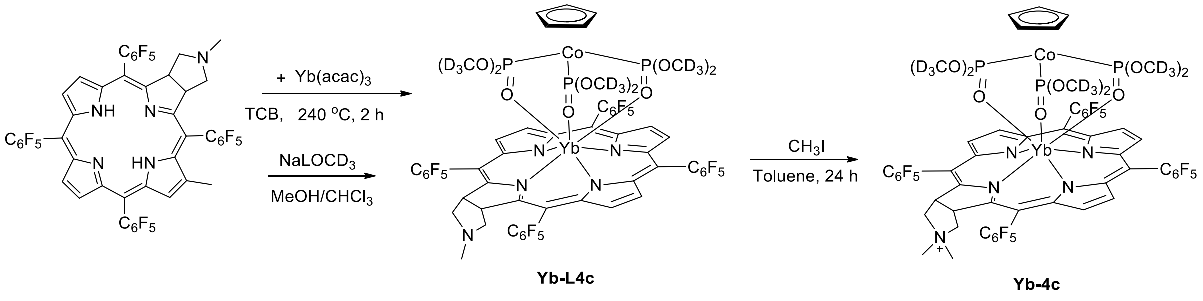

Scheme S8. Synthetic procedure of **Yb-4c**.

Complex **Yb-L4c**: Yield: 41%; ^{1}H NMR (400 MHz, $CDCl_3$) δ -4.28 (s, 5H), 5.70 (s, 2H), 6.71 (s, 2H), 8.53 (s, 3H), 14.19 (s, 2H), 19.02 (s, 2H), 20.15 (s, 2H), 23.02 (s, 2H). ^{19}F NMR (471 MHz, $CDCl_3$) δ -129.96 (d, *J* = 20.8 Hz, 2F), -131.06 (d, *J* = 21.8 Hz, 2F), -134.24 (d, *J* = 23.1 Hz, 2F), -138.52 (dd, *J* = 25.1, 7.9 Hz, 2F), -151.96 (t, *J* = 20.0 Hz, 2F), -152.73 (t, *J* = 20.2 Hz, 2F), -159.66 (t, *J* = 21.1 Hz, 2F), -160.19 (t, *J* = 18.2 Hz, 2F), -160.84 (t, *J* = 18.8 Hz, 2F), -162.35 (t, *J* = 21.6 Hz, 2F). UV/Vis (CH_2Cl_2, 25 °C): λ_{max}(nm) (log ε): 418 (5.71), 517 (4.34), 580 (4.42), 625 (5.20).

Complex **Yb-4c**: Yield: 96%; ^{1}H NMR (400 MHz, CD_2Cl_2) δ -5.18 (s, 5H), 5.03 (s, 2H), 6.35 (s, 2H), 7.53 (s, 3H), 7.71 (s, 3H), 10.56 (s, 2H), 14.62 (s, 2H), 15.44 (s, 2H), 19.25 (s, 2H). ^{19}F NMR (377 MHz, CD_2Cl_2) δ -164.07 (s, 2F), -159.71 (s, 2F), -157.92 (s, 2F), -155.72 (s, 2F), -153.12 (s, 2F), -148.35 (s, 2F), -144.59 (s, 2F), -134.50 (s, 2F), -125.82 (s, 2F), -123.77 (s, 2F). HR-MS (ESI^+) *m/z* $[M]^+$: Calcd for $C_{59}H_{23}D_{18}CoF_{20}N_5O_9P_3Yb$ 1687.1648, Found: 1687.1627; UV/Vis (CH_2Cl_2, 25 °C): λ_{max}(nm) (log ε): 418 (5.62), 516 (4.14), 577 (4.20), 622 (5.12).

Synthesis of Yb-5c.

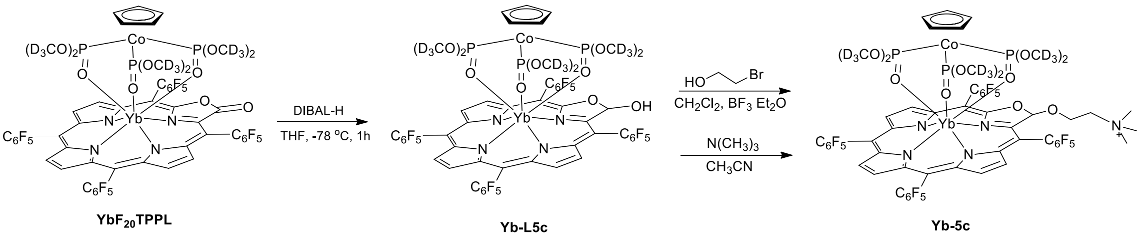

Scheme S9. Synthetic procedure of **Yb-5c**.

Complex **Yb-L5c**: Yield: 86%; ^{1}H NMR (400 MHz, $CDCl_3$) δ -5.34 (s, 5H), 5.43 (s, 1H), 6.26 (s, 1H), 12.78 (s, 1H), 18.16 (s, 1H), 18.56 (s, 1H), 30.58 (s, 1H). ^{19}F NMR (377 MHz, $CDCl_3$) δ -126.48 (d, *J* = 22.6 Hz,2F), -129.43 (d, *J* = 23.1 Hz, 1F), -130.75 (d, *J* = 19.8 Hz, 1F), -132.07 (d, *J* = 20.3 Hz, 1F), -134.30 (d, *J* = 22.1 Hz, 1F), -140.53 (d, *J* = 22.2 Hz, 1F), -141.51 (d, *J* = 20.5 Hz, 1F), -150.86 (t, *J* = 19.2 Hz, 1F), -152.06 (m, 3F), -158.99 (t, *J* = 19.6 Hz, 1F), -159.61 (t, *J* = 20..1 Hz, 2F), -159.88 (t, *J* = 23.2 Hz, 2F), -160.26 (t, *J* = 22.2 Hz, 1F), -162.35 (t, *J* = 21.5 Hz, 1F), -162.61 (t, *J* = 22.6 Hz, 1F). UV/Vis (CH_2Cl_2, 25 °C): λ_{max}(nm) (log ε): 417 (5.56), 517 (4.11), 580 (4.17), 624 (5.05).

Complex **Yb-5c**: Yield: 45%; ^{1}H NMR (400 MHz, $CDCl_3$) δ -5.16 (s, 5H), 3.73 (s, 9H), 5.71 (s, 1H), 6.55 (s, 1H), 7.10 (s, 2H), 7.66 (s, 1H), 7.97 (s, 1H), 11.14 (s, 1H), 11.97 (s, 1H), 18.51 (s, 1H), 18.97 (s, 1H), 29.46 (s, 1H). ^{19}F NMR (471 MHz, $CDCl_3$) δ -126.98 (d, *J* = 19.0, 1F), -127.51 (d, *J* = 20.1, 1F), -129.51 (d, *J* = 20.5, 1F), -130.08 (d, *J* = 21.3, 1F), -133.24 (d, *J* = 19.3, 1F), -136.41 (d, *J* = 21.7, 1F), -140.58 (d, *J* = 21.1, 1F), -141.35 (d, *J* = 20.5, 1F), -148.17 (t, 1F), -149.88 (t, 1F), -151.75 (t, 1F), -151.98 (t, 1F), -156.52 (t, 1F), -158.16 (t, 1F), -159.39 (t, 1F), - 159.57 (t, 1F), -160.49 (t, 1F), -160.97 (t, 1F), -162.23 (t, 1F), -162.49 (t, 1F). HR-MS (ESI^+) *m/z* $[M]^+$: Calcd for $C_{59}H_{25}D_{18}CoF_{20}N_5O_{11}P_3Yb$ 1721.1703, Found: 1721.1688; UV/Vis (DMSO, 25 °C): λ_{max}(nm) (log ε): 416 (5.57), 513 (4.10), 577 (4.15), 623 (5.06).

Table S1. Lipophilicity (log $P_{o/w}$) of **Yb-1-5** and **Yb-2c-5c**.

Compound	**Yb-1**	**Yb-2**	**Yb-3**	**Yb-4**	**Yb-5**	**Yb-2c**	**Yb-3c**	**Yb-4c**	**Yb-5c**
log $P_{o/w}$	1.32	1.41	0.56	1.34	1.48	1.26	0.24	1.33	1.33

Table S2. Determination of cellular uptake in HeLa cells by ICP.

Sample	Concentration of Yb^{3+} complex in cell cedia	Theoretical concentration of Yb^{3+} in cell media (μg/mL)	Concentration of Yb^{3+} in $1x10^6$ cells (μg/mL)
**blank**	0	0	0±0.0136
**Yb-1**	10 μM	1.74	0.0259±0.0217
**Yb-2**	10 μM	1.74	0.1872±0.0456
**Yb-3**	10 μM	1.74	0.1542±0.0388
**Yb-4**	10 μM	1.74	0.1286±0.0441
**Yb-5**	10 μM	1.74	0.1057±0.0364
**Yb-2c**	10 μM	1.74	0.2190±0.0549
**Yb-3c**	10 μM	1.74	0.2796±0.0431
**Yb-4c**	10 μM	1.74	0.2578±0.0272
**Yb-5c**	10 μM	1.74	0.3203±0.0376

Table S3. Decay lifetime (τ_{obs}) monitored at 980 nm of Yb-1-5 in water and 0.2 M bovine albumin (BSA) under air-saturated condition.[a,b]

Solution	**Yb-1**	**Yb-2**	**Yb-3**	**Yb-4**	**Yb-5**
H_2O	95(2)	173(5)	56(2)	140(5)	64(2)
BSA	100(3)	181(4)	90(2)	145(3)	74(3)

[a] Standard error values are given in parentheses; they refer to the reproducibility of the measurements. Experimental relative errors: τ_{obs}, ±5%; [b] Measured with 0.1% DMSO.

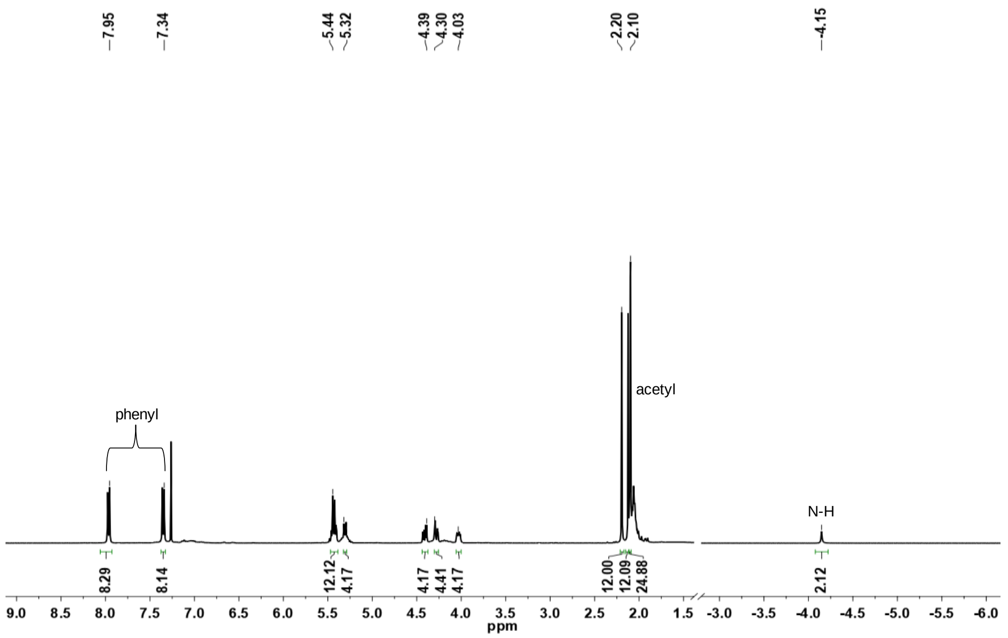

Fig. S1 ^{1}H NMR spectrum (400 MHz) of **L1** in $CDCl_3$.

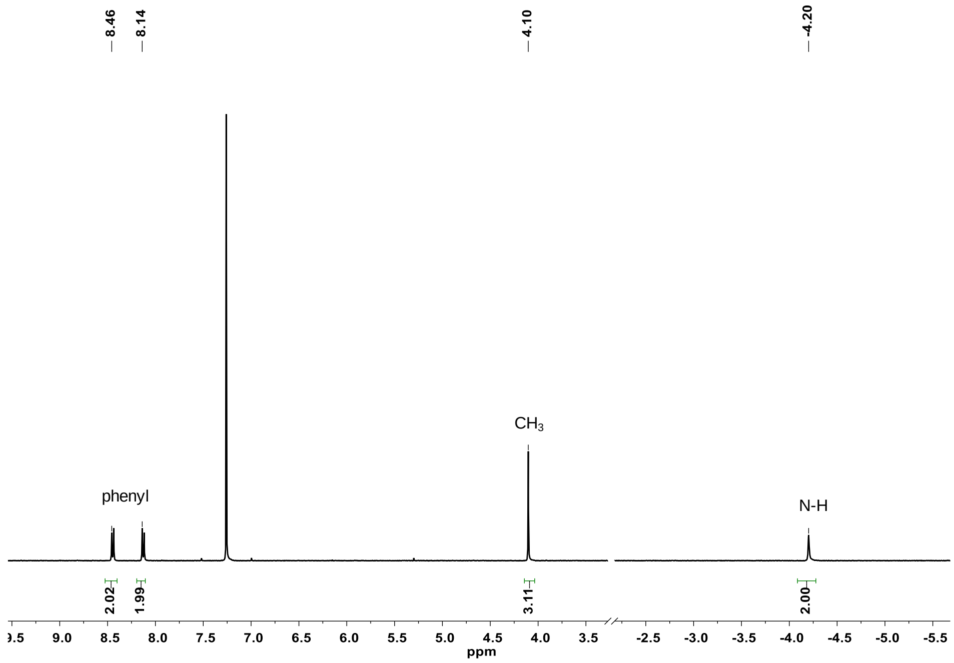

Fig. S2 ^{1}H NMR spectrum (400 MHz) of **L2** in $CDCl_3$.

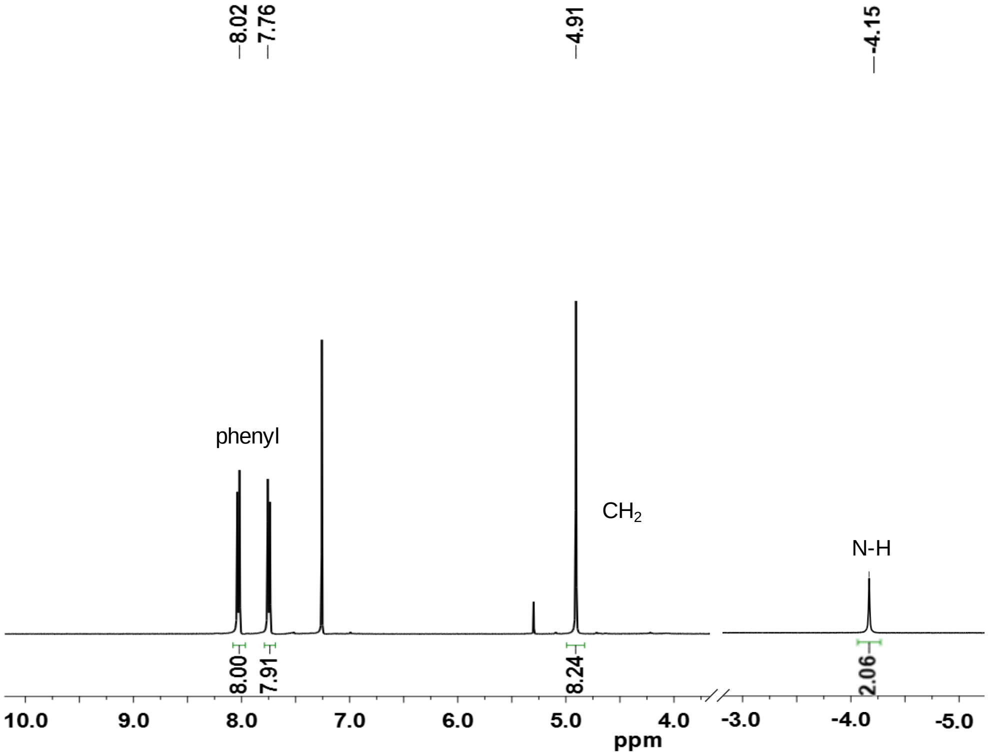

Fig. S3 ^{1}H NMR spectrum (400 MHz) of **L3** in $CDCl_3$.

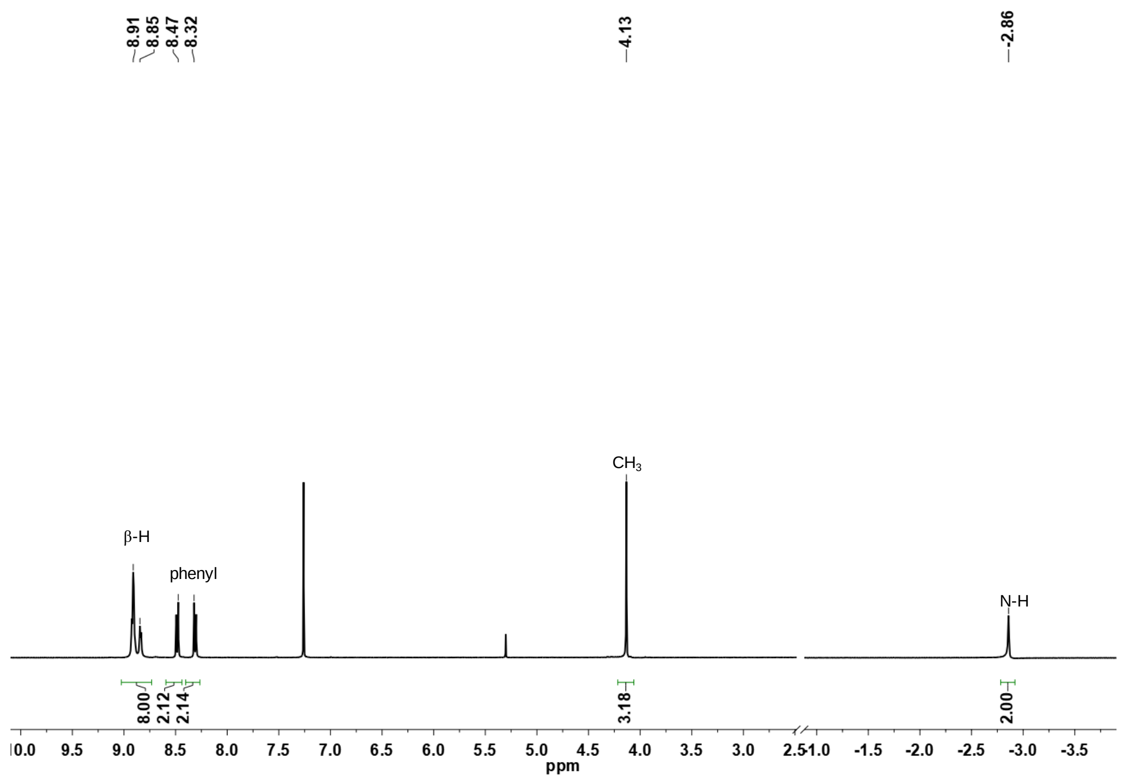

Fig. S4 ^{1}H NMR spectrum (400 MHz) of **L2c** in $CDCl_3$.

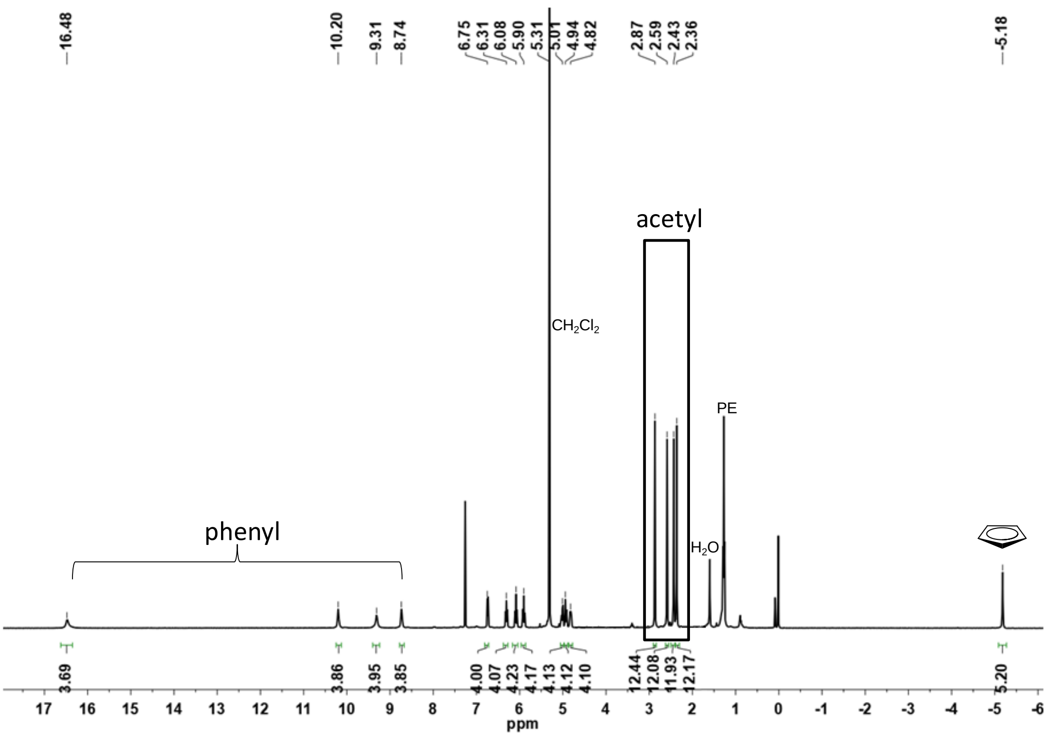

Fig. S5 ^{1}H NMR spectrum (400 MHz) of **Yb-L1** in $CDCl_3$.

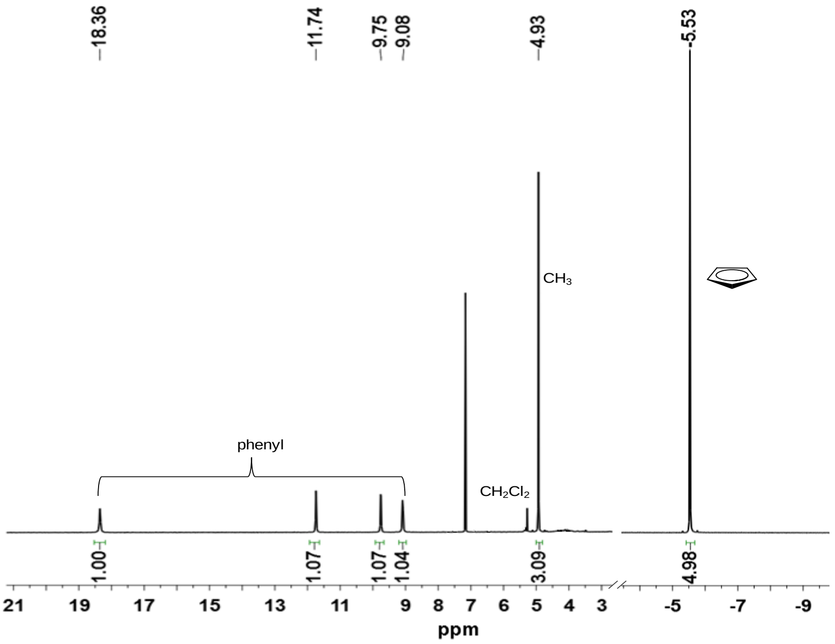

Fig. S6 ^{1}H NMR spectrum (400 MHz) of **Yb-L2** in $CDCl_3$.

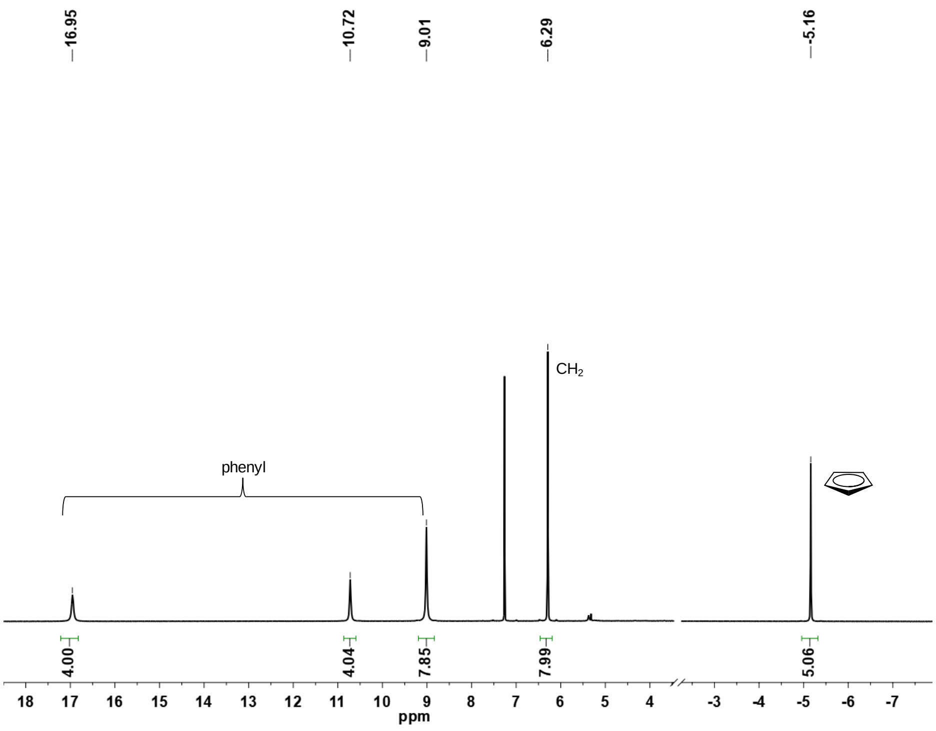

Fig. S7 ^{1}H NMR spectrum (400 MHz) of **Yb-L3** in $CDCl_3$.

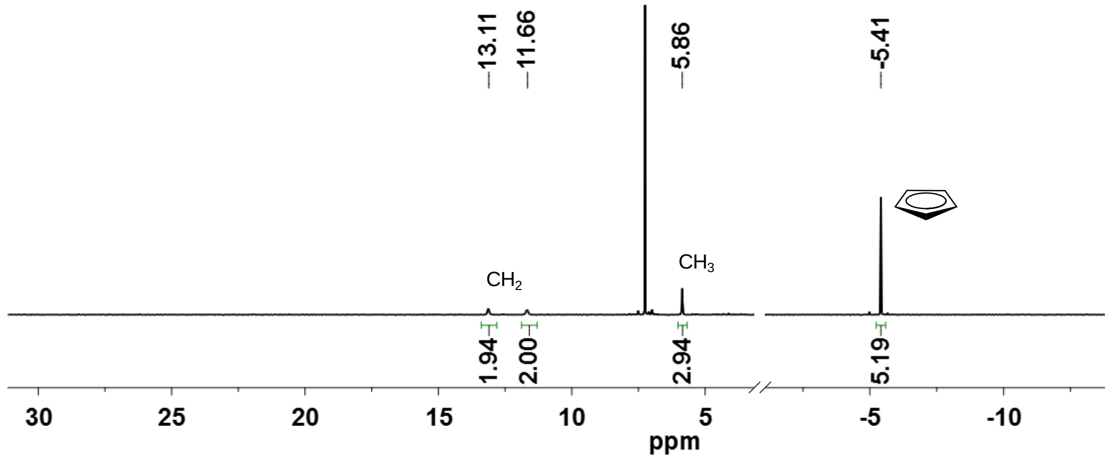

Fig. S8 ^{1}H NMR spectrum (400 MHz) of **Yb-L4** in $CDCl_3$.

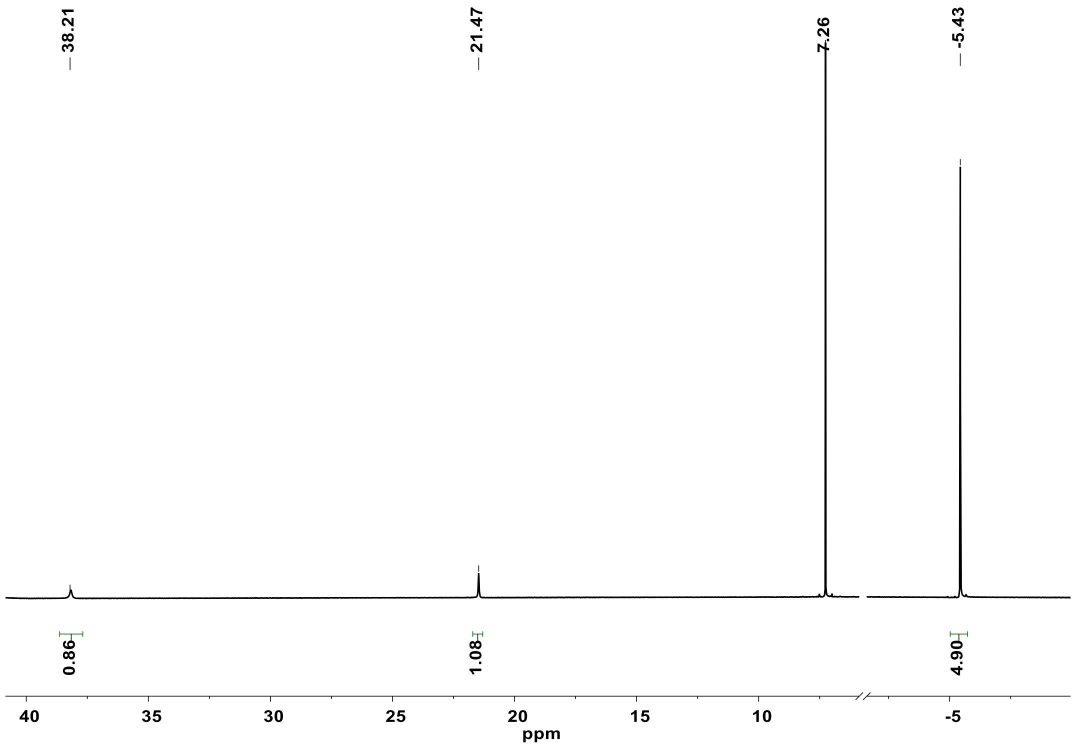

Fig. S9 ^{1}H NMR spectrum (400 MHz) of **Yb-L5** in $CDCl_3$.

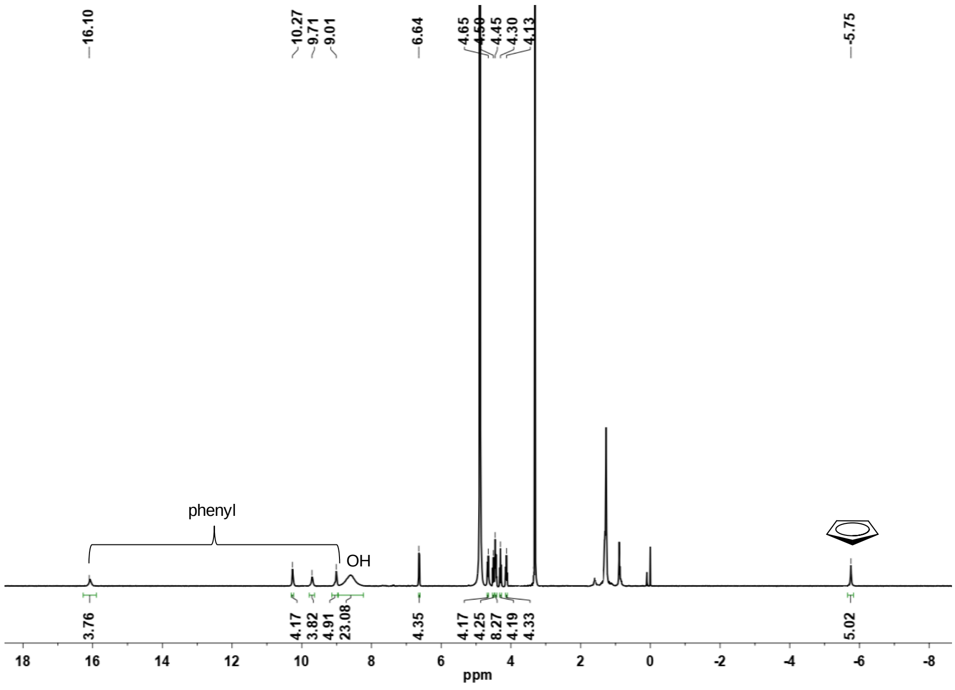

Fig. S10 ^{1}H NMR spectrum (400 MHz) of **Yb-1** in CD_3OD.

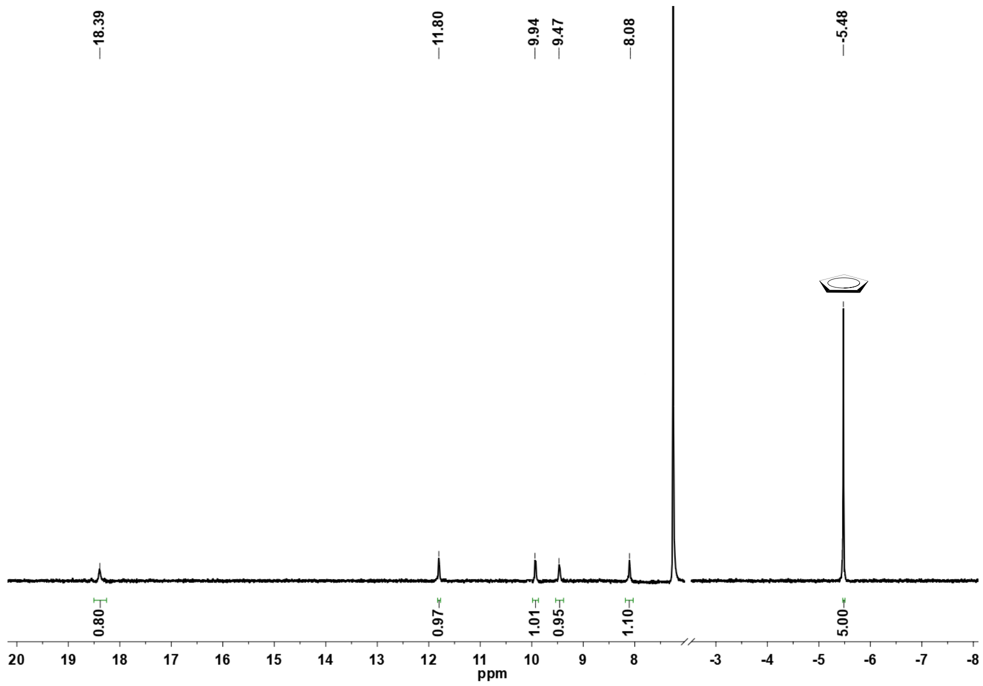

Fig S11 ^{1}H NMR spectrum (400 MHz) of **Yb-2** in $CDCl_3$.

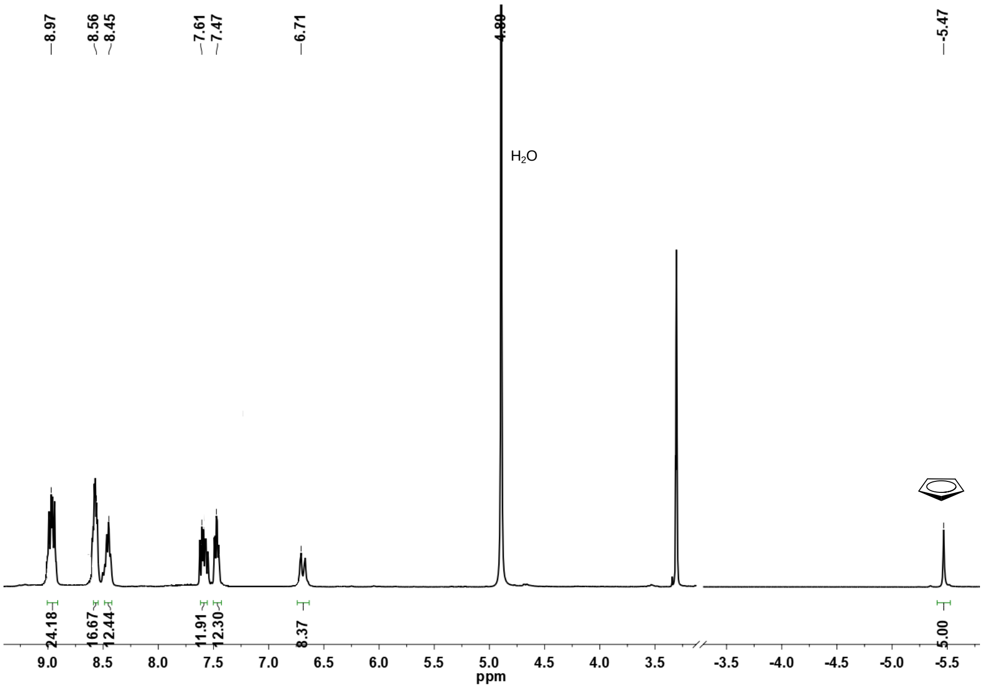

Fig. S12 ^{1}H NMR spectrum (400 MHz) of **Yb-3** in CD_3OD.

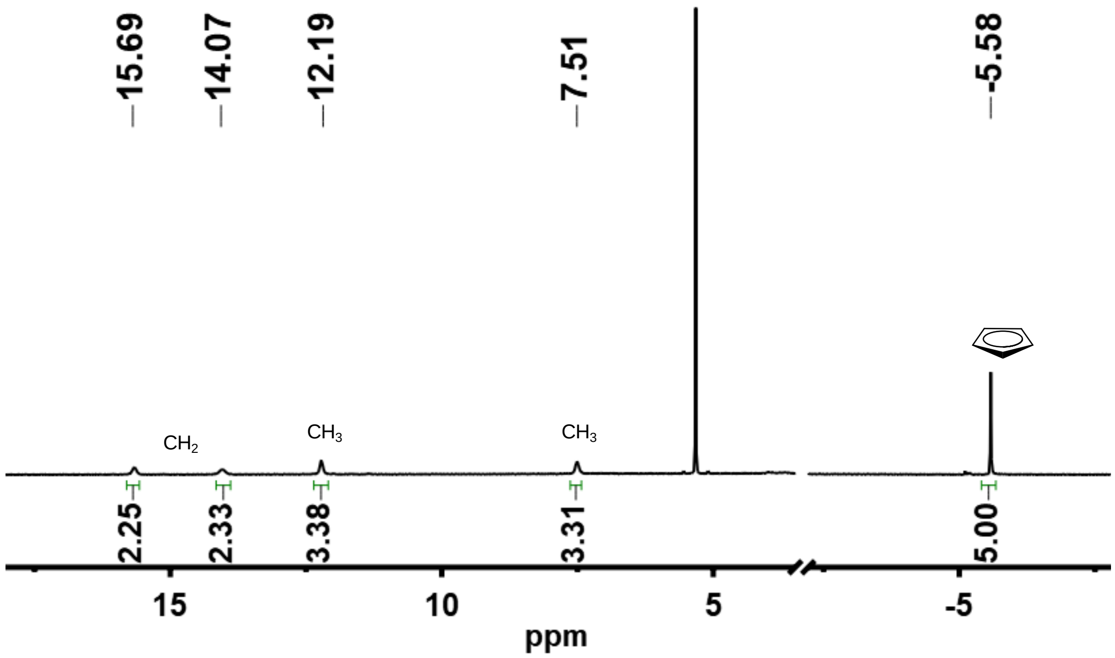

Fig. S13 ^{1}H NMR spectrum (400 MHz) of **Yb-4** in CD_2Cl_2.

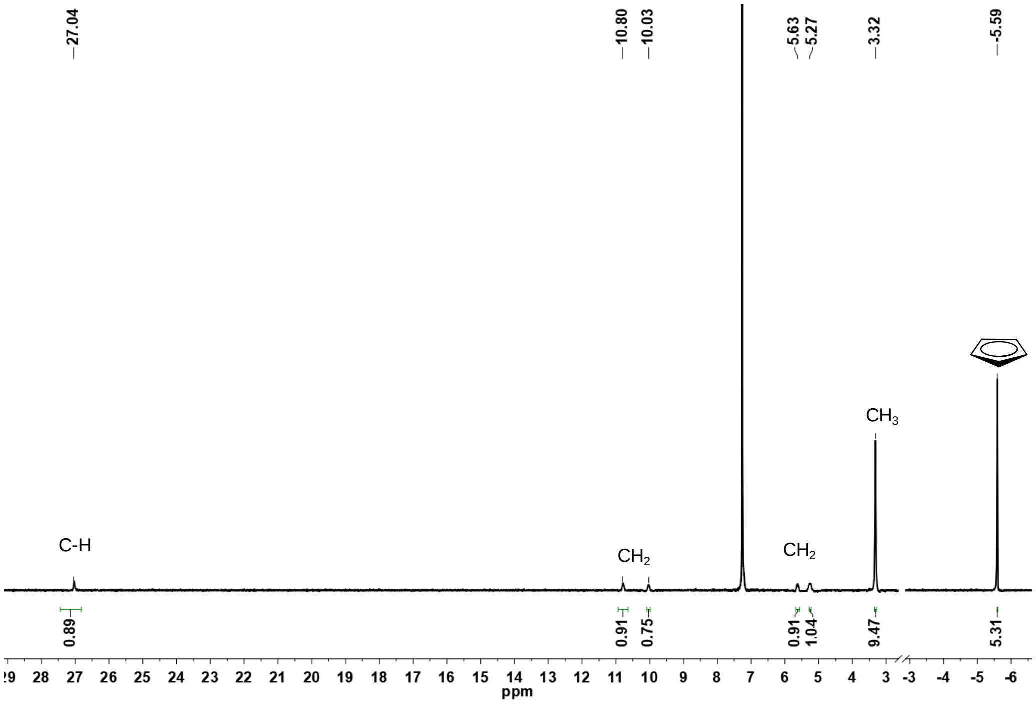

Fig. S14 ^{1}H NMR spectrum (400 MHz) of **Yb-5** in $CDCl_3$.

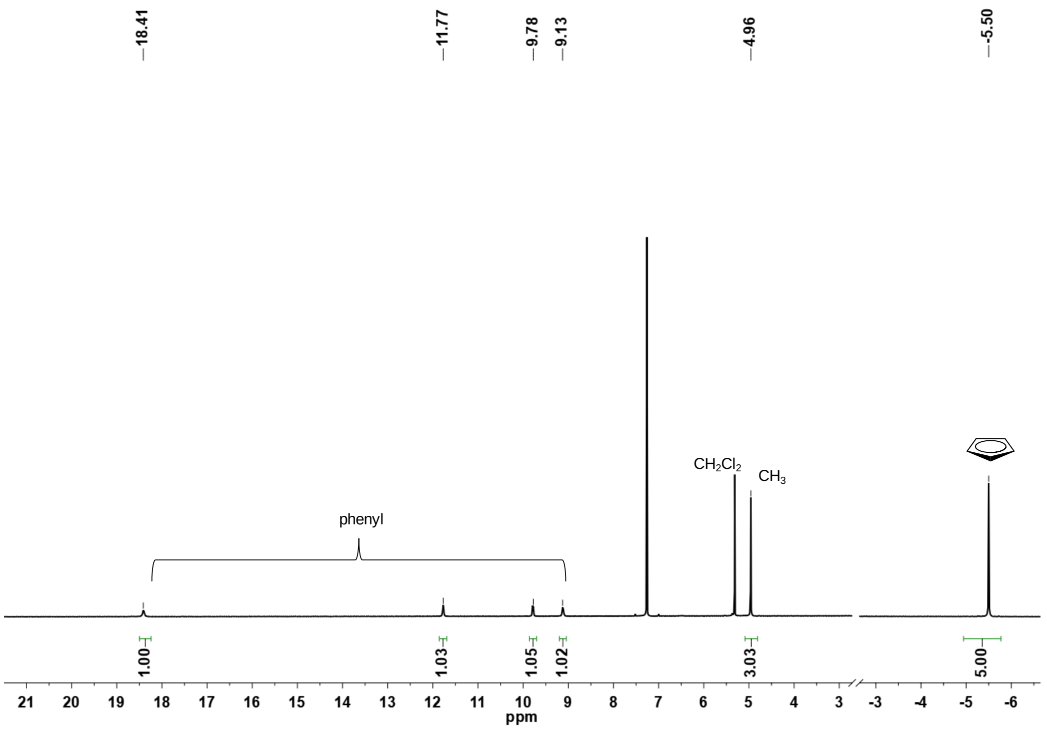

Fig. S15 ^{1}H NMR spectrum (400 MHz) of **Yb-L2c** in $CDCl_3$.

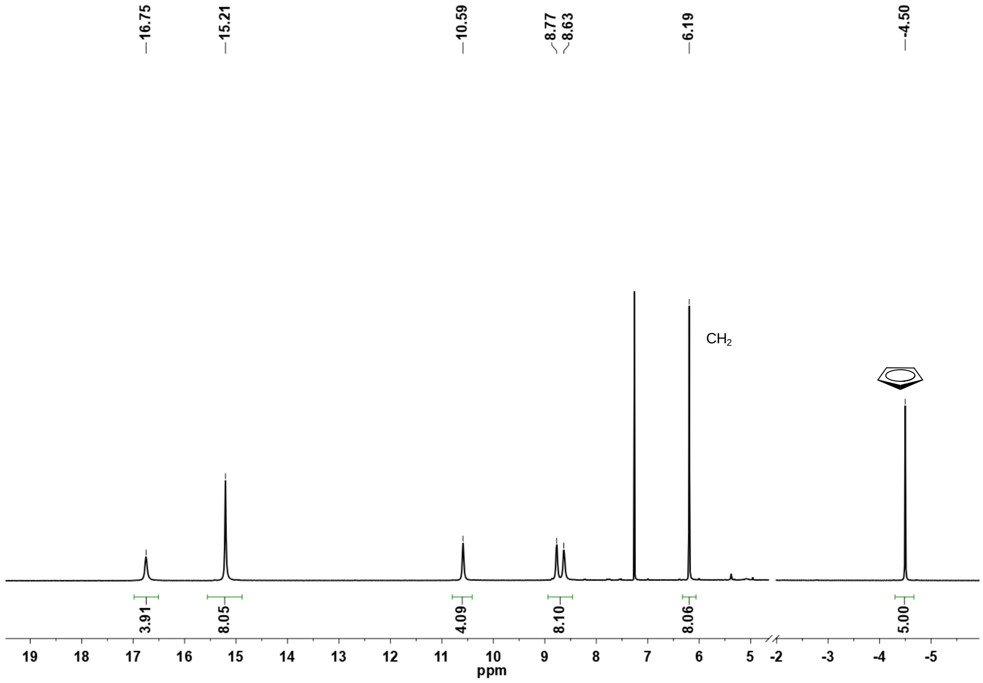

Fig. S16 ^{1}H NMR spectrum (400 MHz) of **Yb-L3c** in $CDCl_3$.

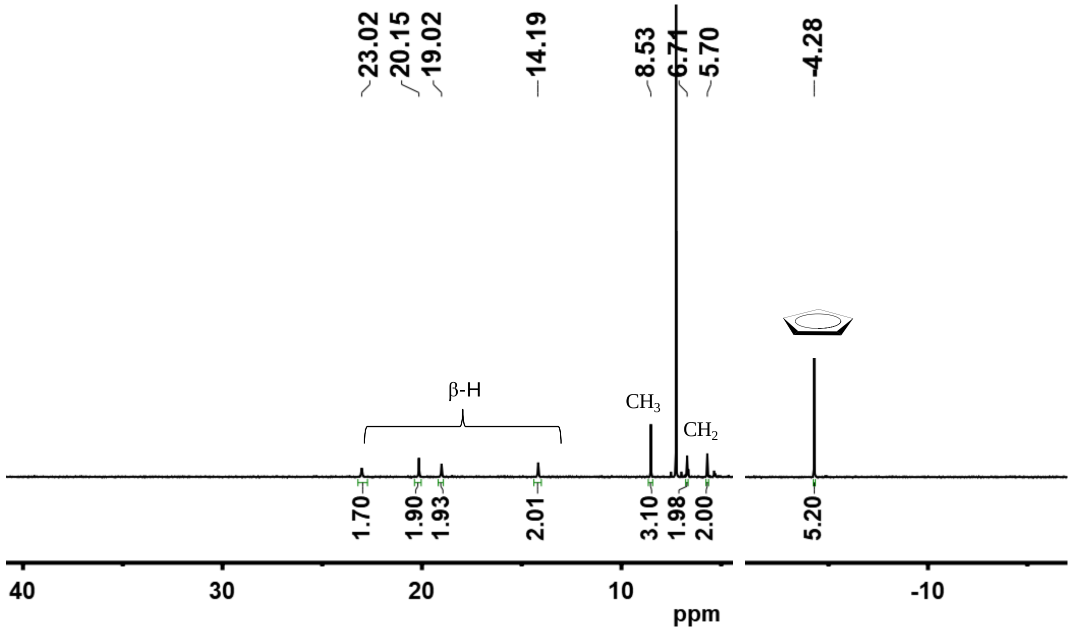

Fig. S17 ^{1}H NMR spectrum (400 MHz) of **Yb-L4c** in $CDCl_3$.

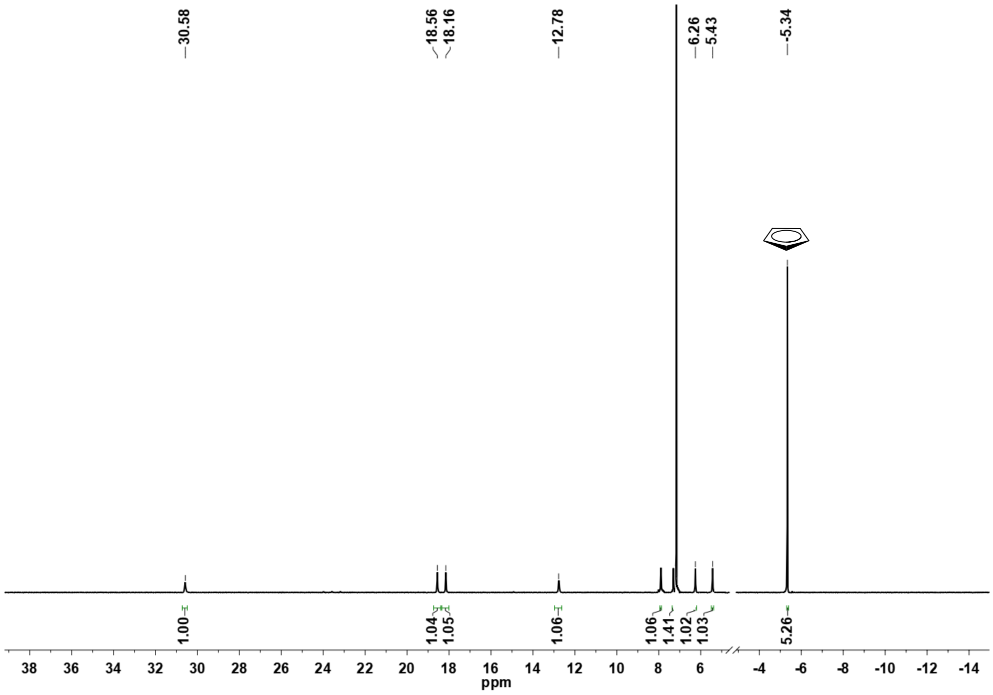

Fig. S18 ^{1}H NMR spectrum (400 MHz) of **Yb-L5c** in $CDCl_3$.

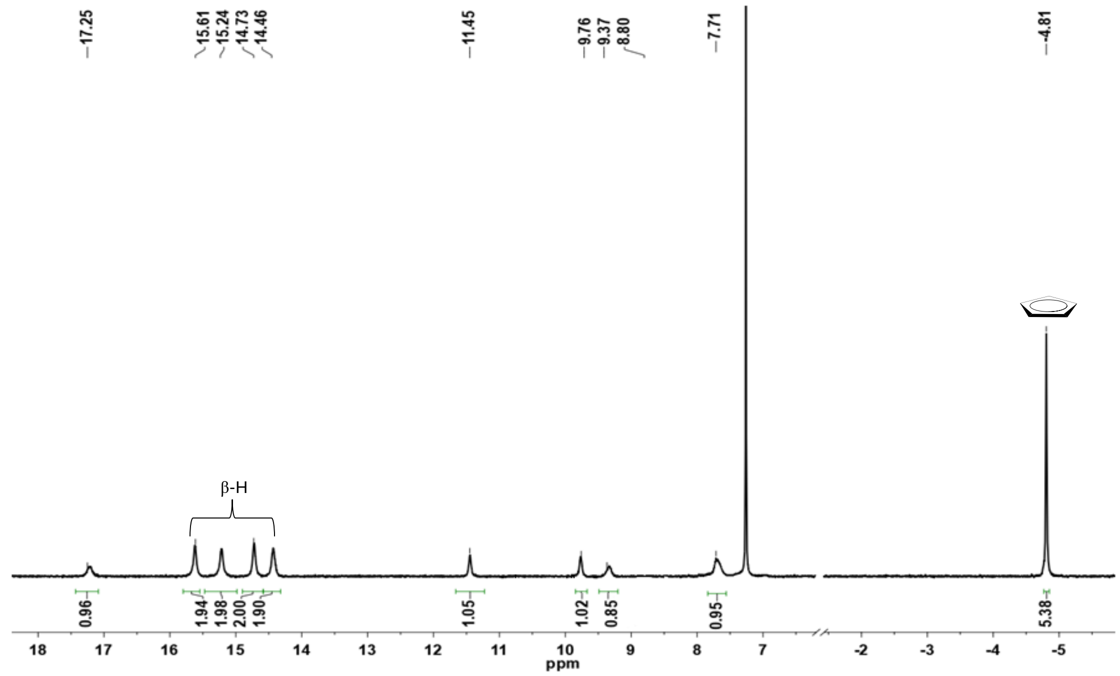

Fig. S19 ^{1}H NMR spectrum (400 MHz) of **Yb-2c** in $CDCl_3$.

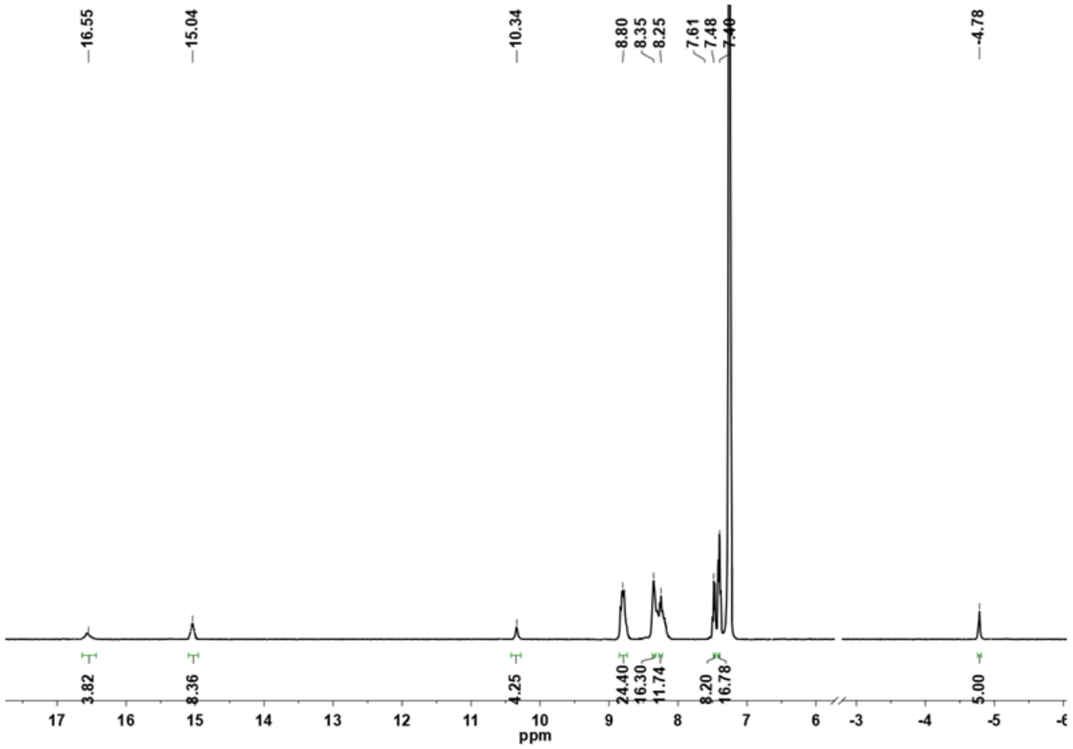

Fig. S20 ^{1}H NMR spectrum (400 MHz) of **Yb-3c** in $CDCl_3$.

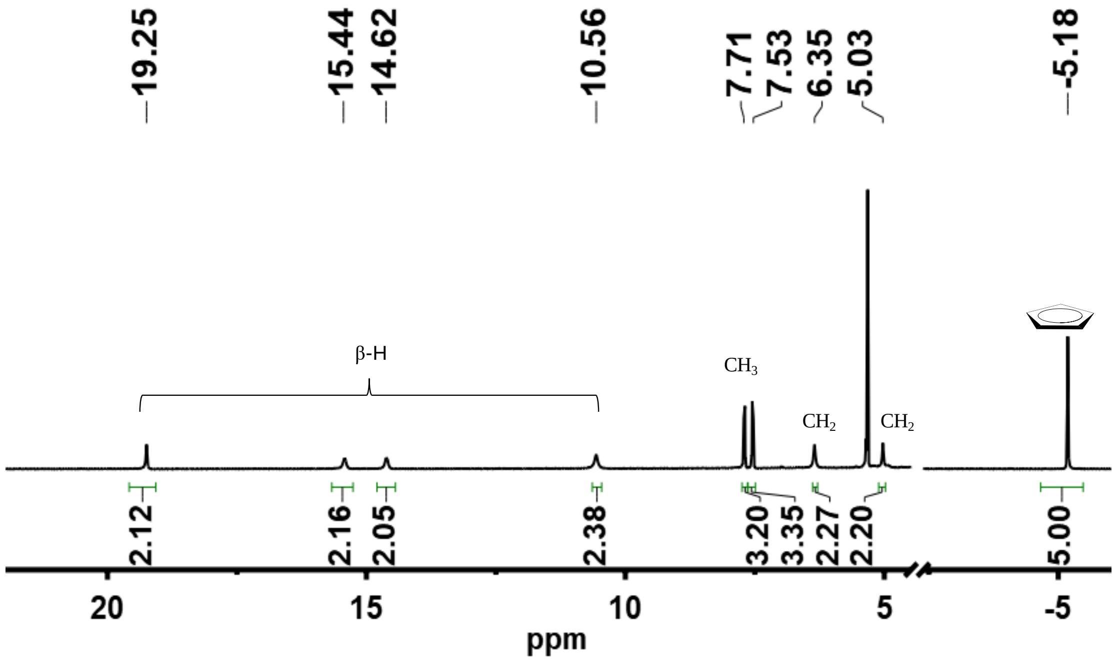

Fig. S21 ^{1}H NMR spectrum (400 MHz) of **Yb-4c** in CD_2Cl_2.

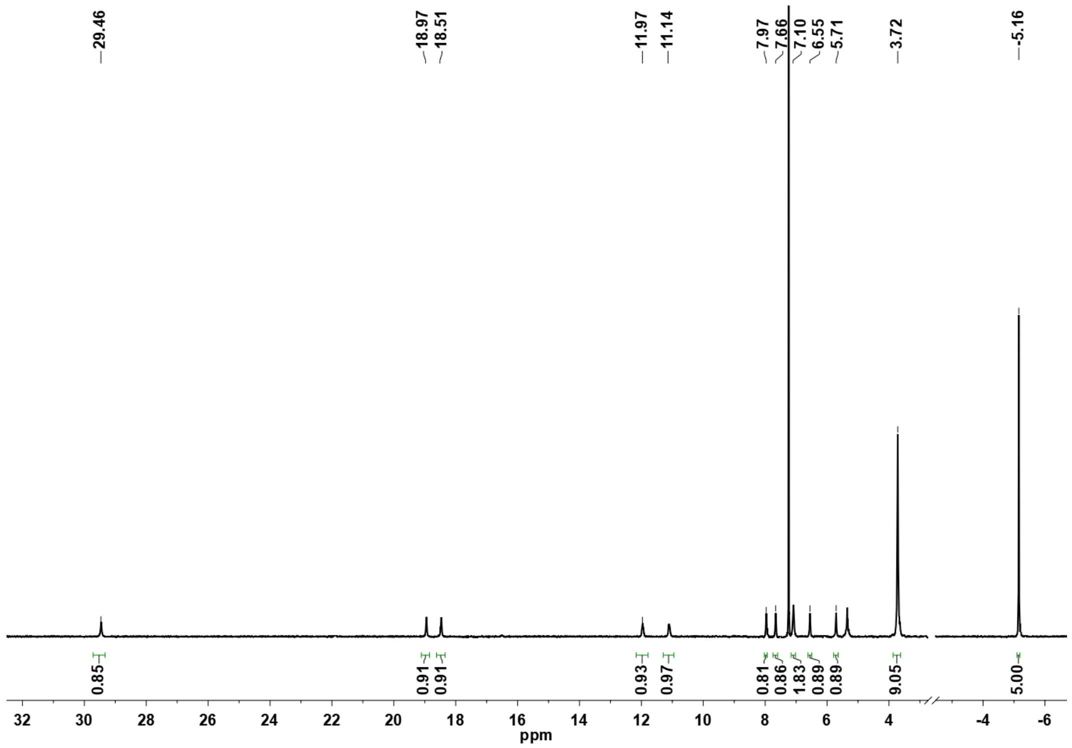

Fig. S22 ^{1}H NMR spectrum (400 MHz) of **Yb-5c** in $CDCl_3$.

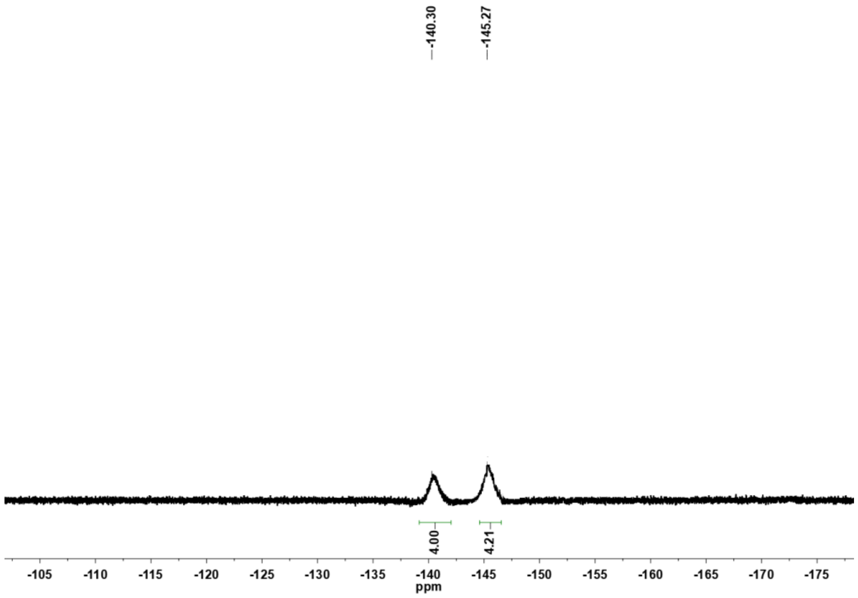

Fig. S23 ^{19}F NMR spectrum (377 MHz) of **L1** in $CDCl_3$.

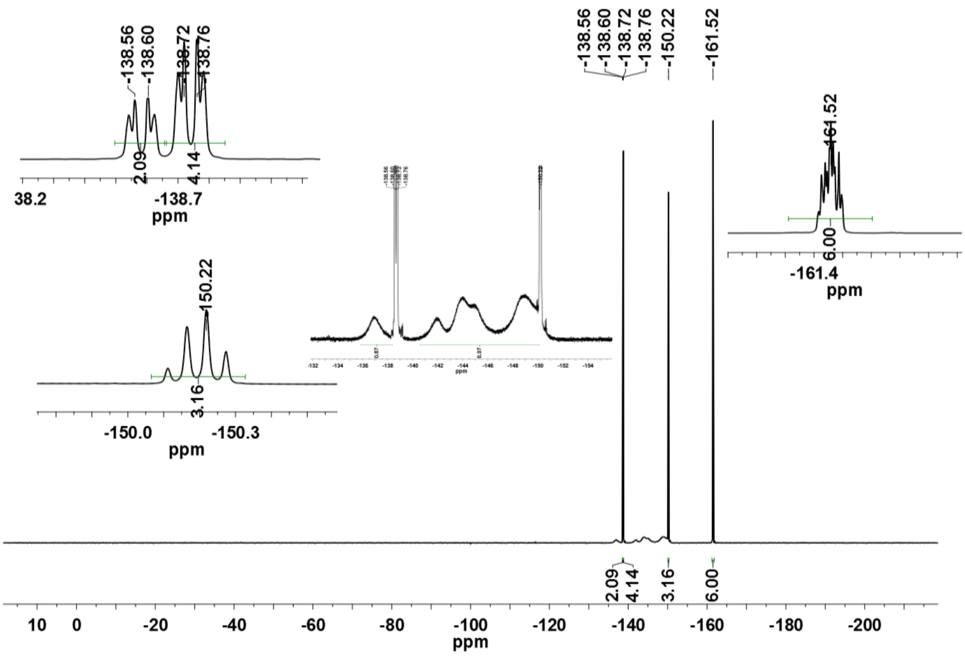

Fig. S24 ^{19}F NMR spectrum (377 MHz) of **L2** in $CDCl_3$.

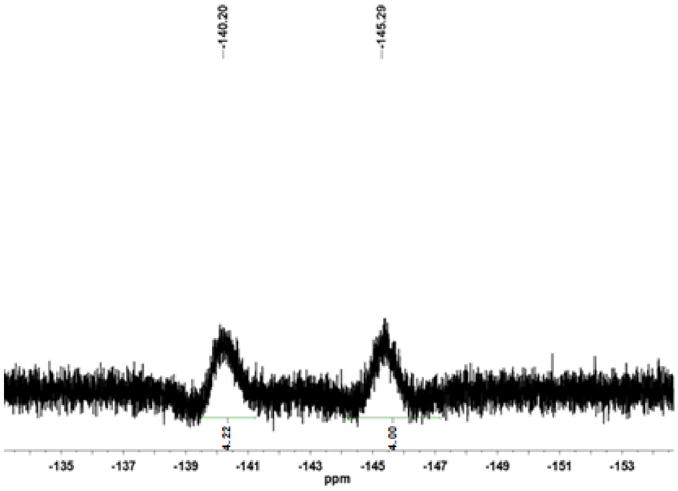

Fig. S25 ^{19}F NMR spectrum (377 MHz) of **L3** in $CDCl_3$.

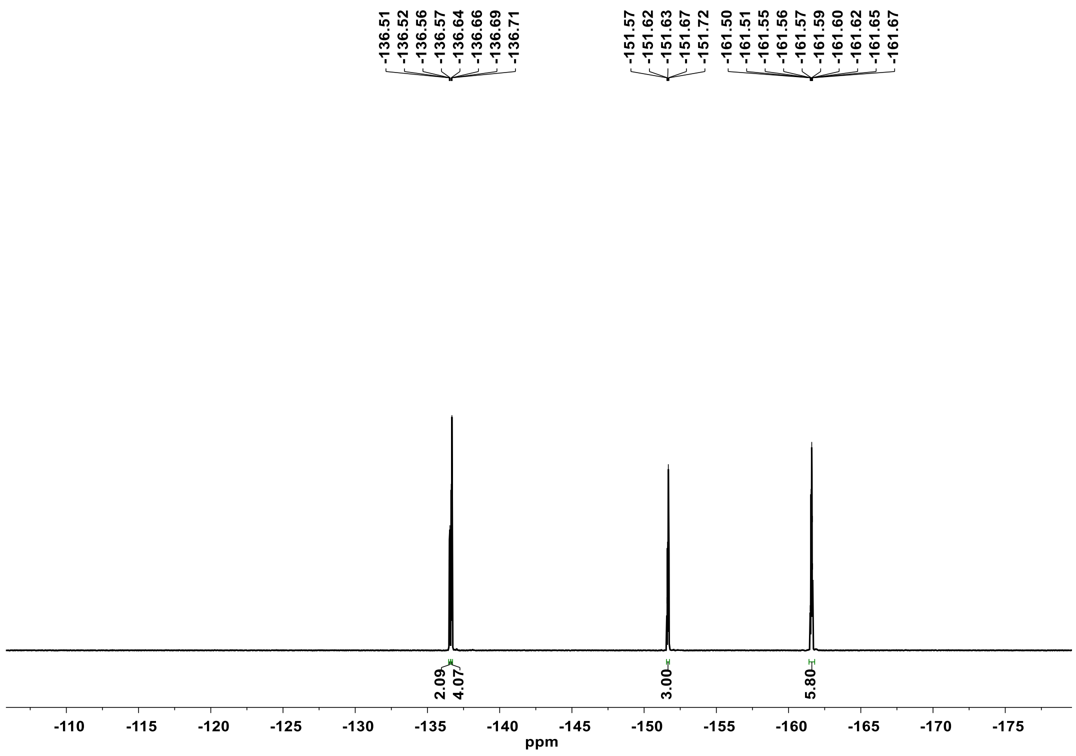

Fig. S26 ^{19}F NMR spectrum (471 MHz) of **L2c** in $CDCl_3$.

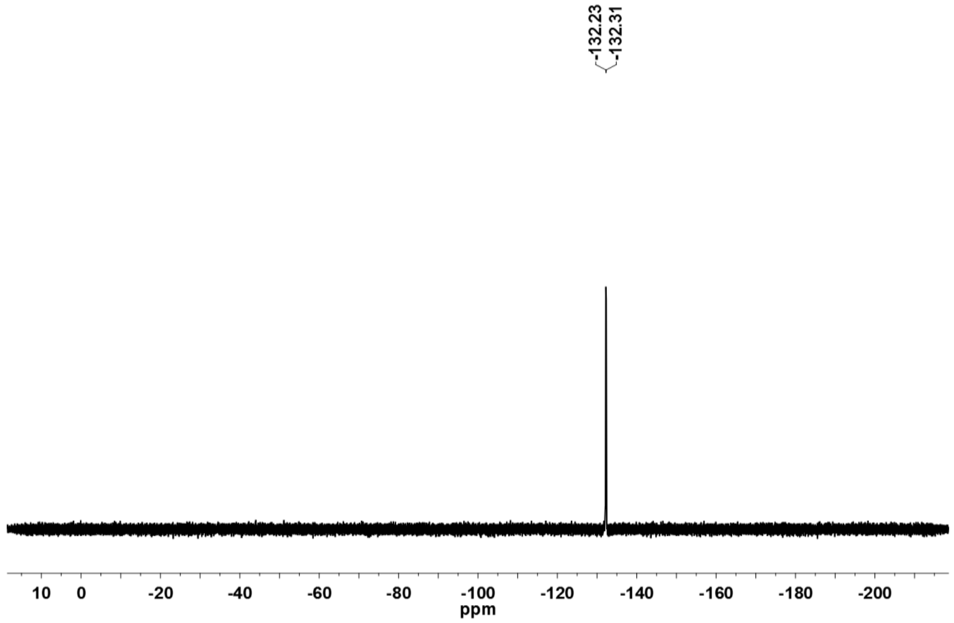

Fig. S27 ^{19}F NMR spectrum (377 MHz) of **Yb-L1** in $CDCl_3$.

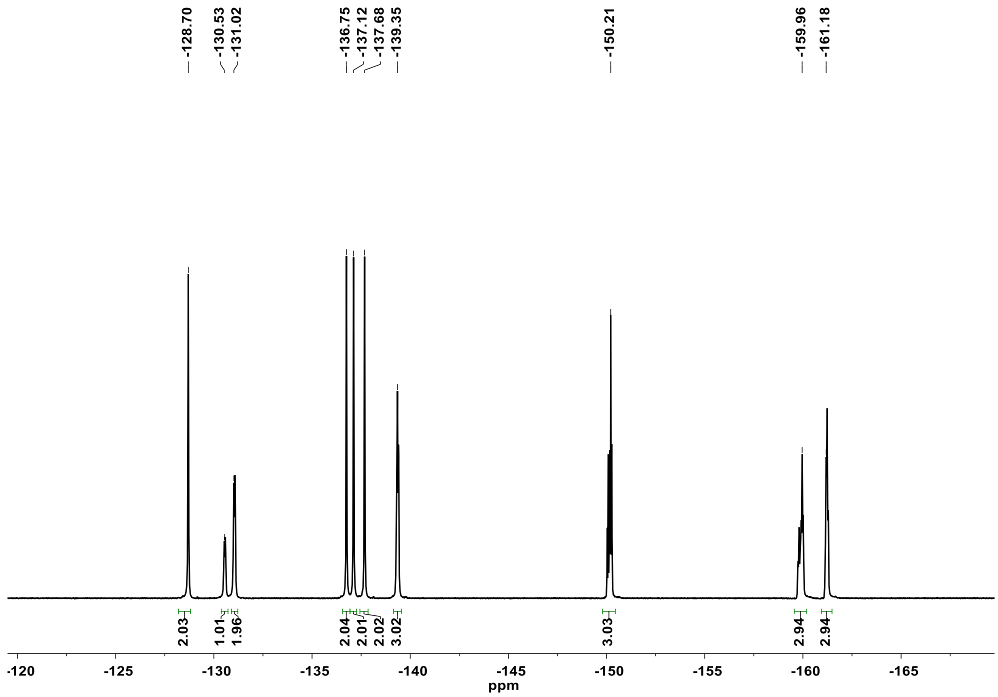

Fig. S28 ^{19}F NMR spectrum (377 MHz) of **Yb-L2** in $CDCl_3$.

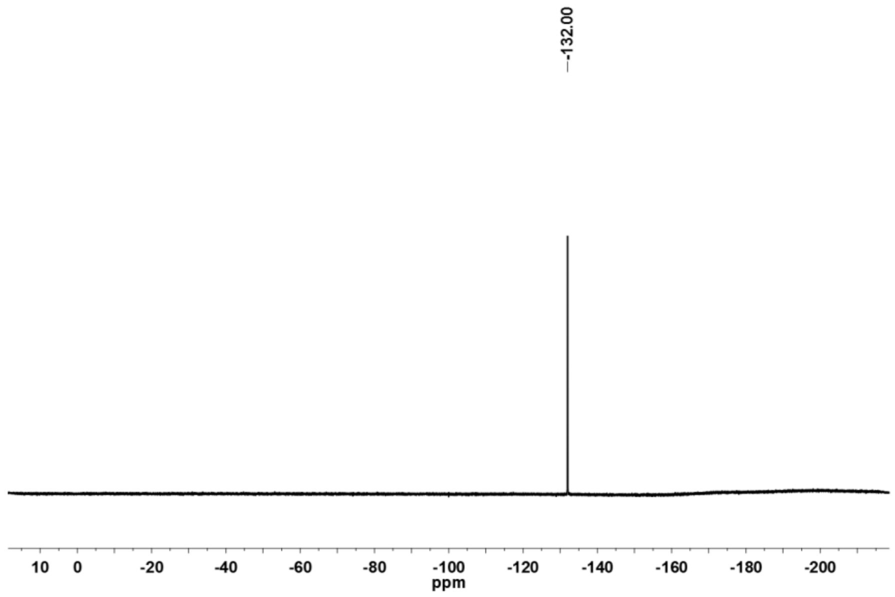

Fig. S29 ^{19}F NMR spectrum (377 MHz) of **Yb-L3** in $CDCl_3$.

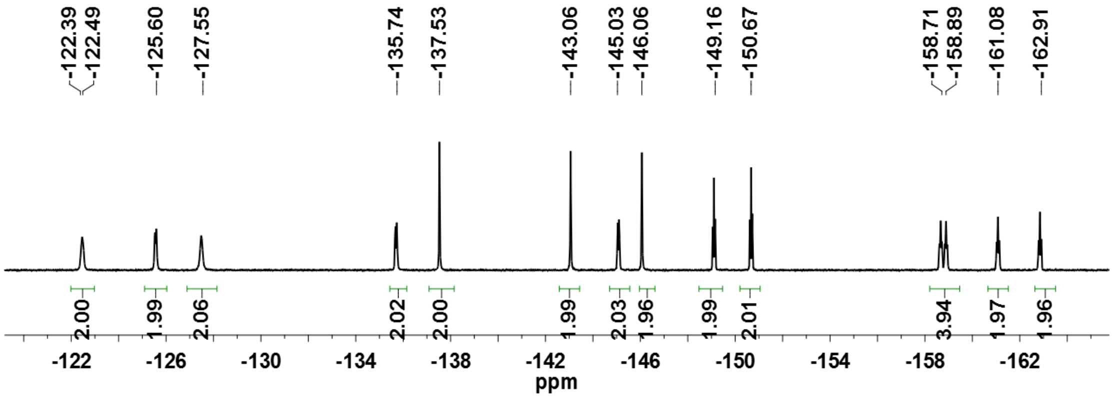

Fig. S30 ^{19}F NMR spectrum (471 MHz) of **Yb-L4** in $CDCl_3$.

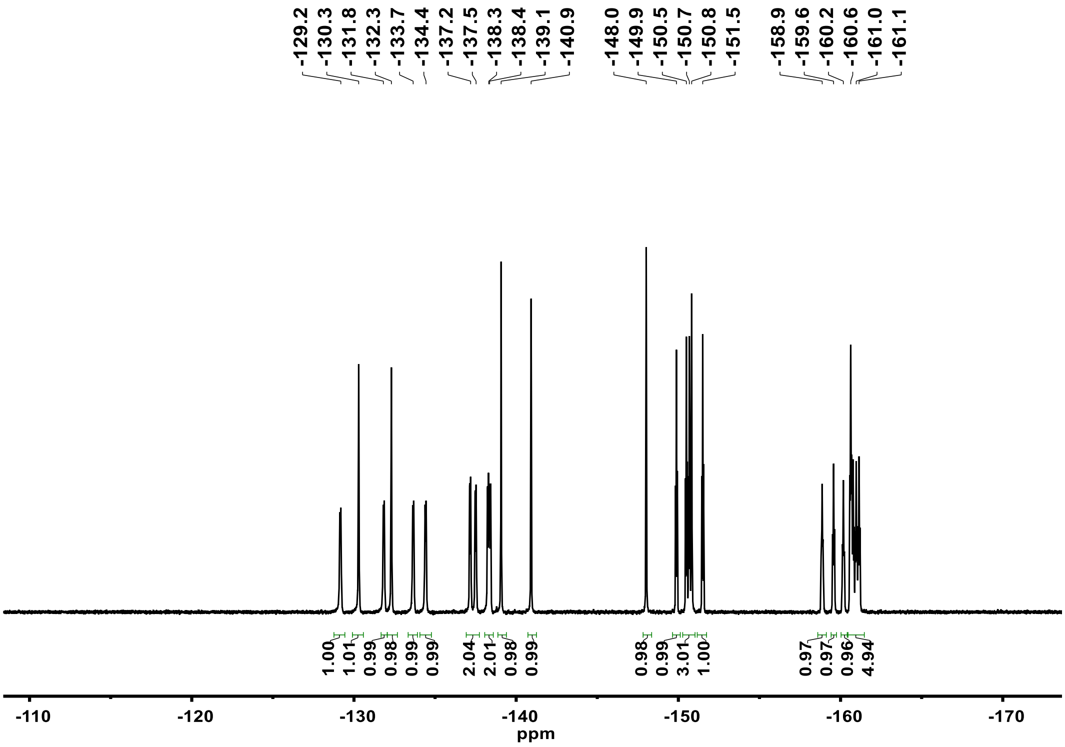

Fig. S31 ^{19}F NMR spectrum (471 MHz) of **Yb-L5** in $CDCl_3$.

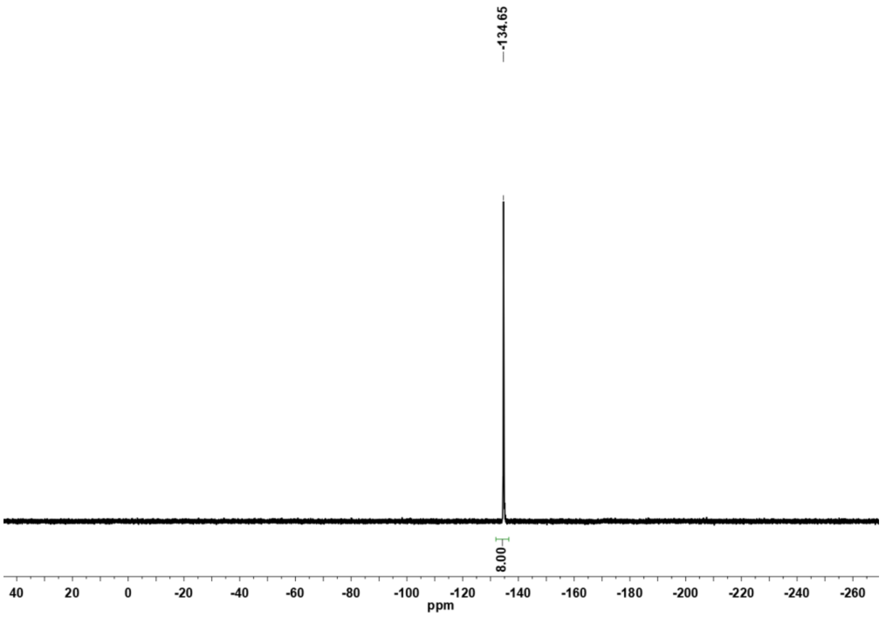

Fig. S32 ^{19}F NMR spectrum (377 MHz) of **Yb-1** inCD_3OD.

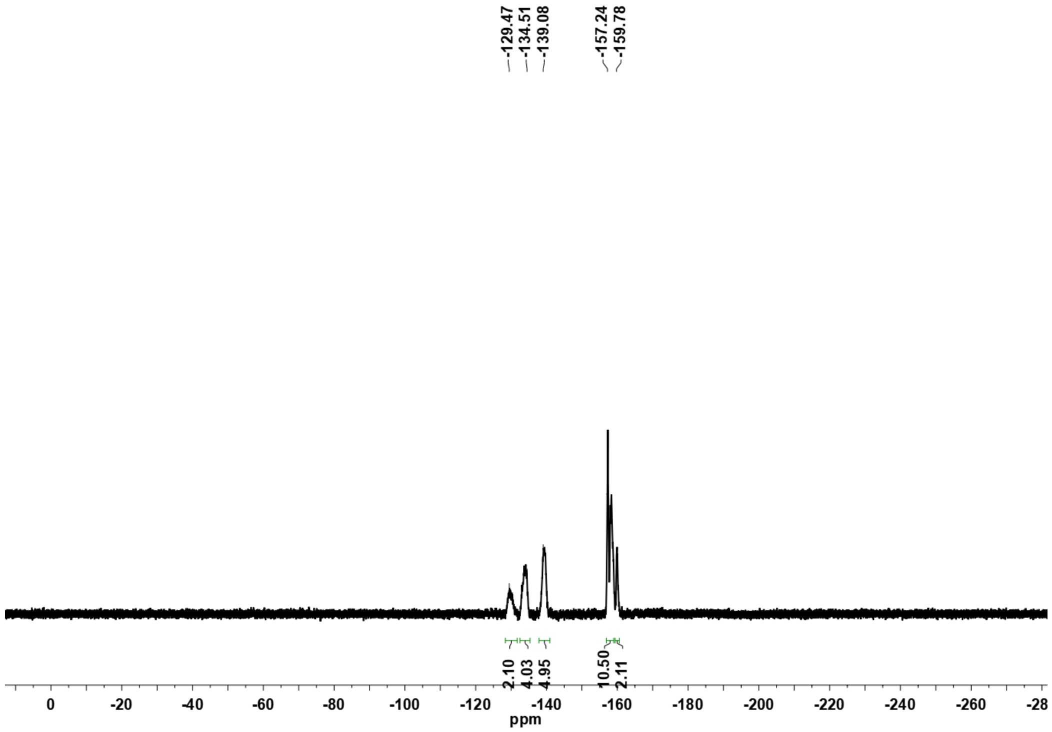

Fig. S33 ^{19}F NMR spectrum (377 MHz) of **Yb-2** in $CDCl_3$.

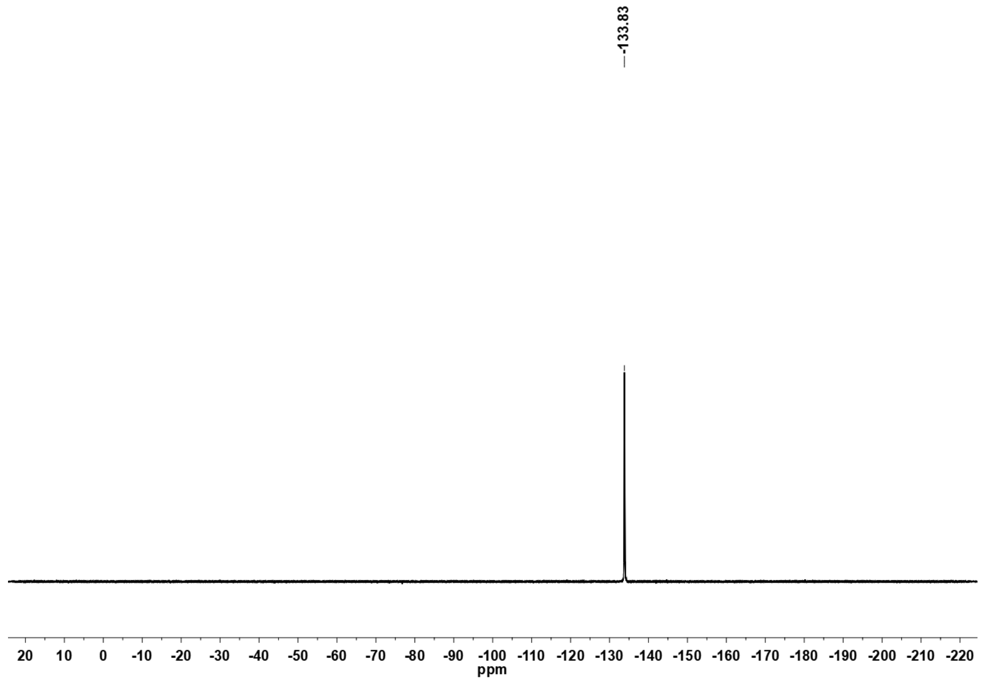

Fig. S34 ^{19}F NMR spectrum (377 MHz) of **Yb-3** in CD_3OD.

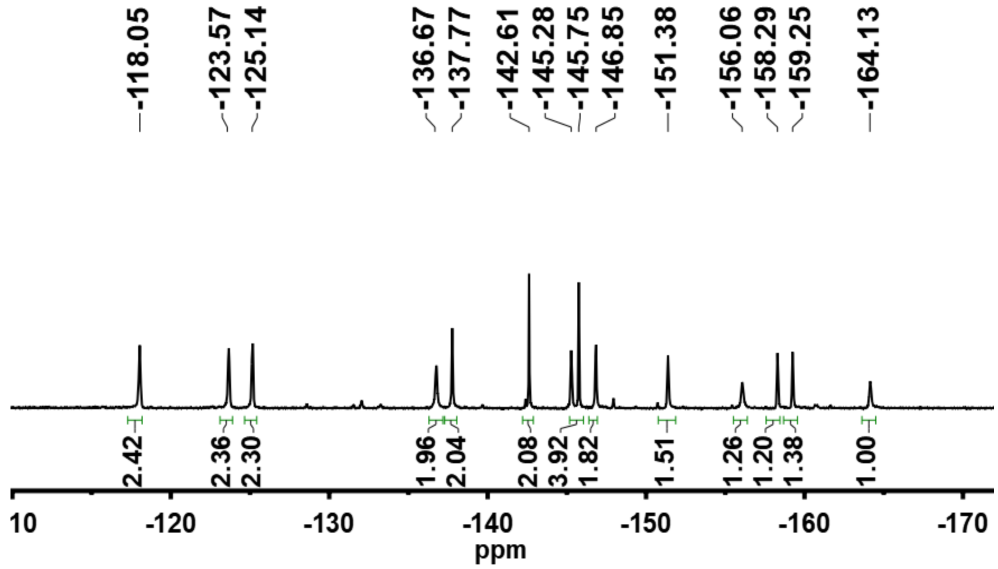

Fig. S35 ^{19}F NMR spectrum (471 MHz) of **Yb-4** in CD_2Cl_2.

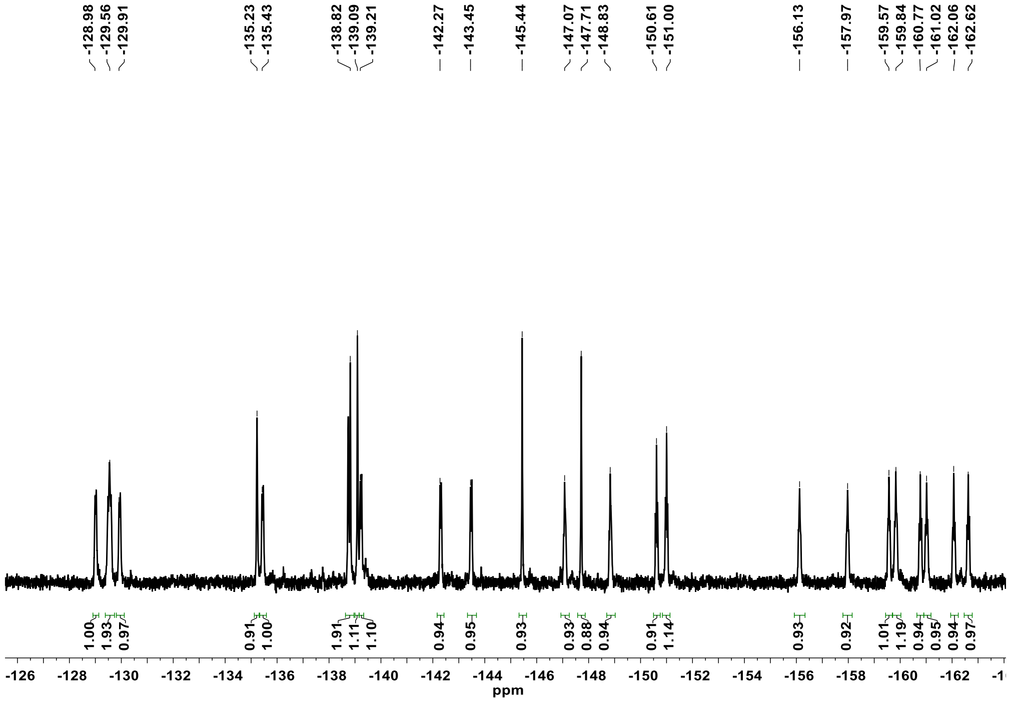

Fig. S36 ^{19}F NMR spectrum (471 MHz) of **Yb-5** in $CDCl_3$.

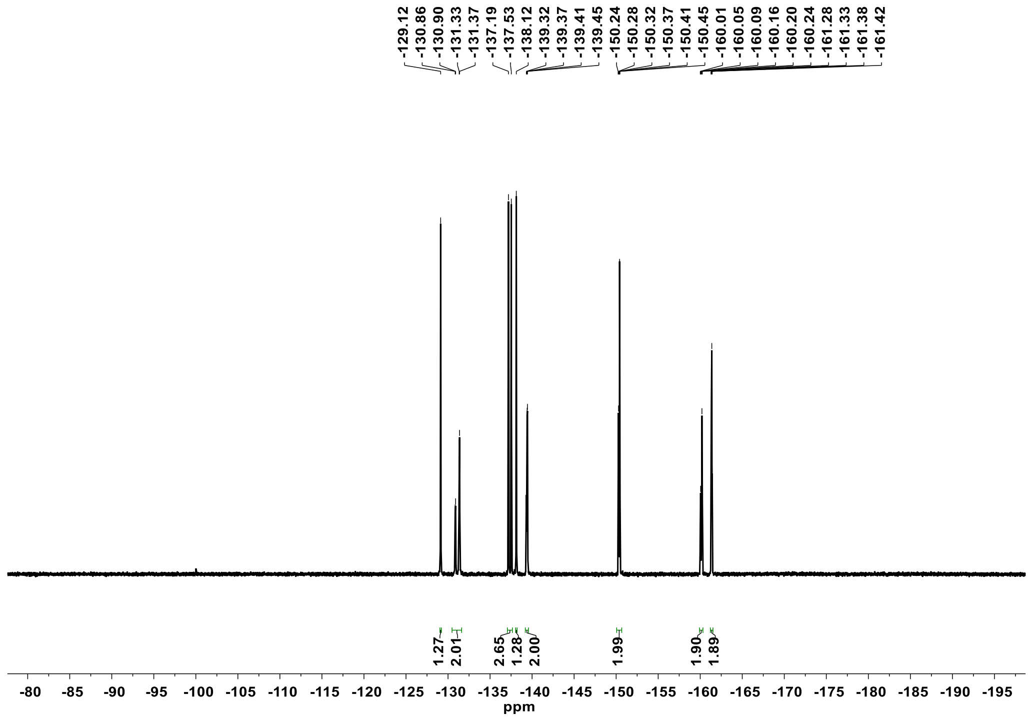

Fig. S37 ^{19}F NMR spectrum (471 MHz) of **Yb-L2c** in $CDCl_3$.

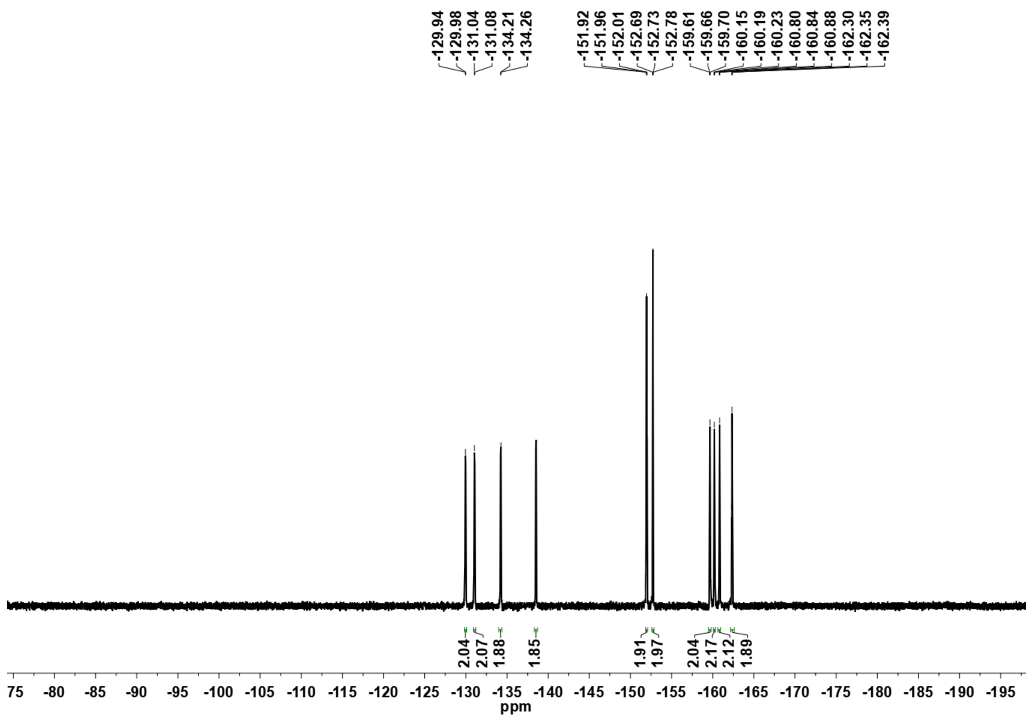

Fig. S38 ^{19}F NMR spectrum (471 MHz) of **YbL4c** in $CDCl_3$.

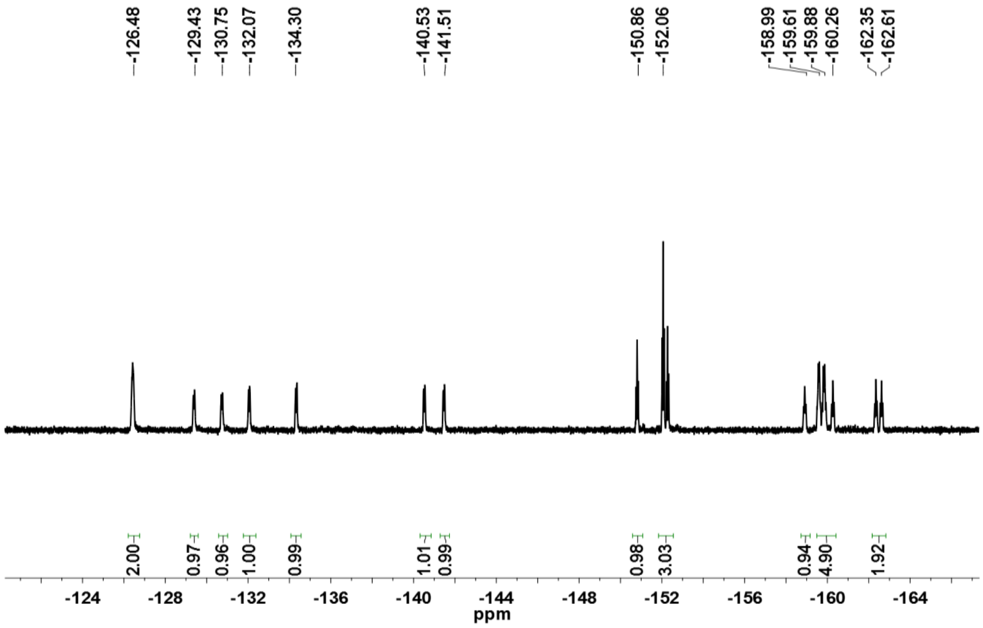

Fig. S39 ^{19}F NMR spectrum (471 MHz) of **YbL5c** in $CDCl_3$.

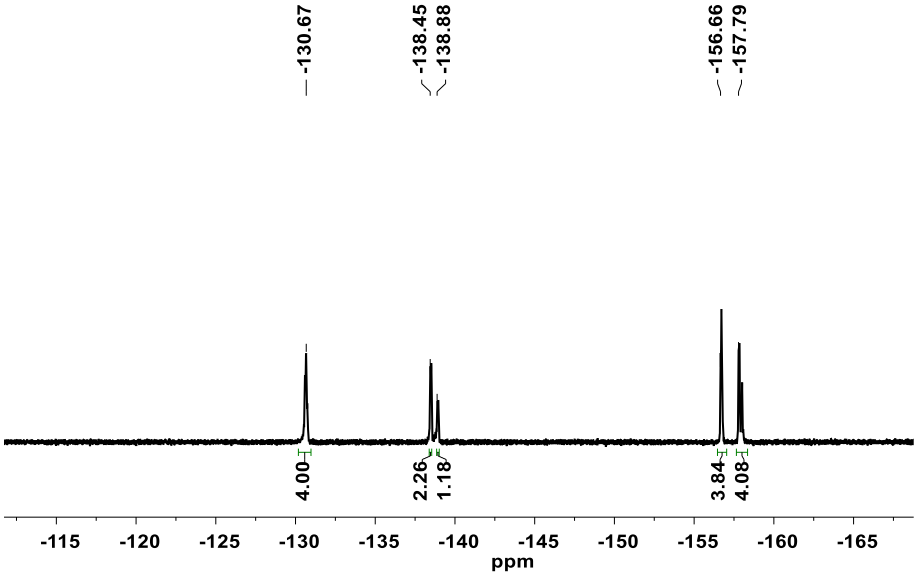

Fig. S40 ^{19}F NMR spectrum (377 MHz) of **Yb-2c** in $CDCl_3$.

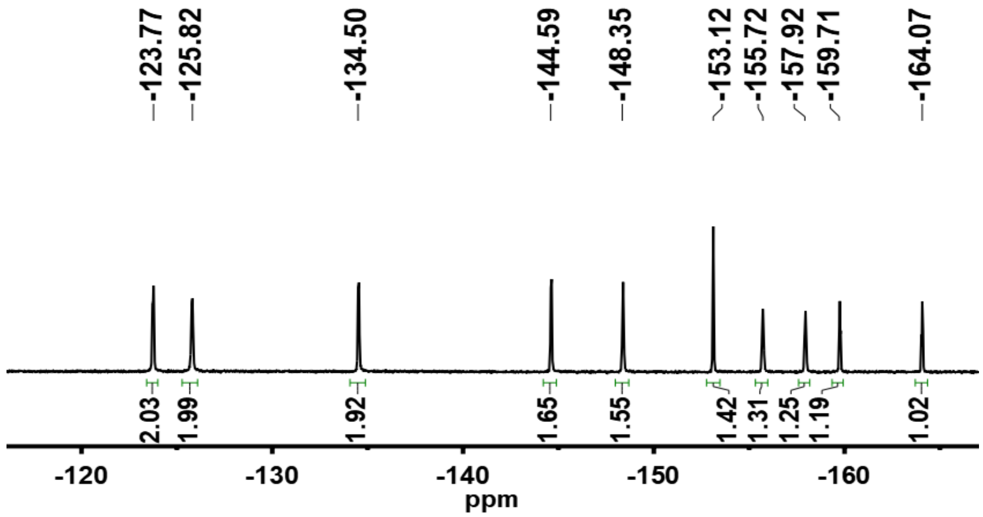

Fig. S41 ^{19}F NMR spectrum (377 MHz) of **Yb-4c** in CD_2Cl_2.

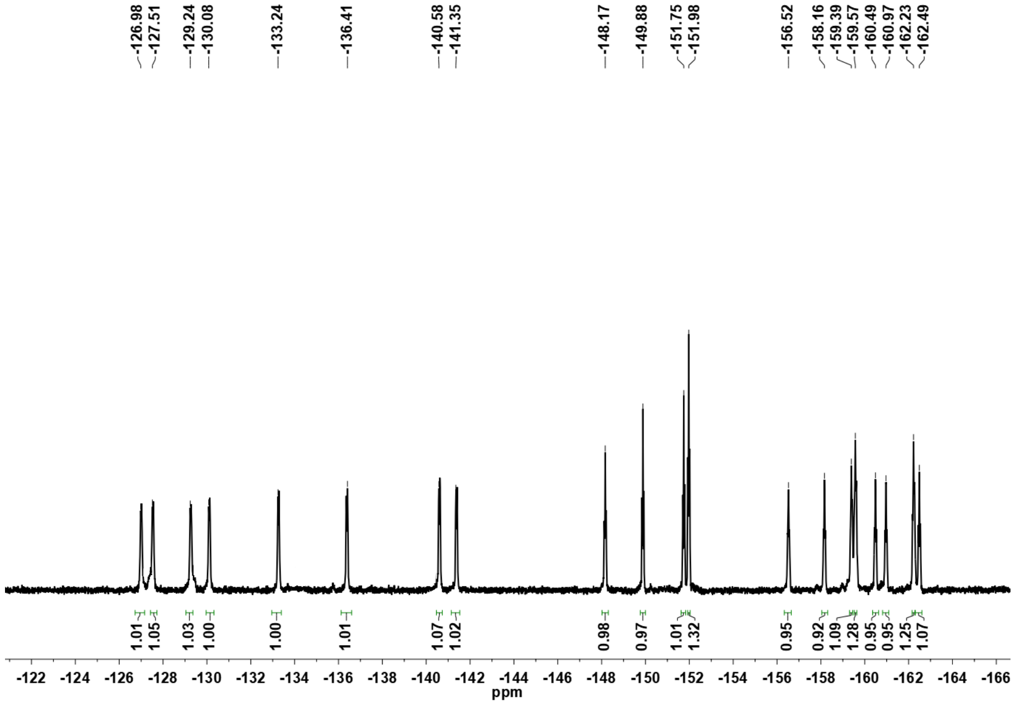

Fig. S42 ^{19}F NMR spectrum (377 MHz) of **Yb-5c** in $CDCl_3$.

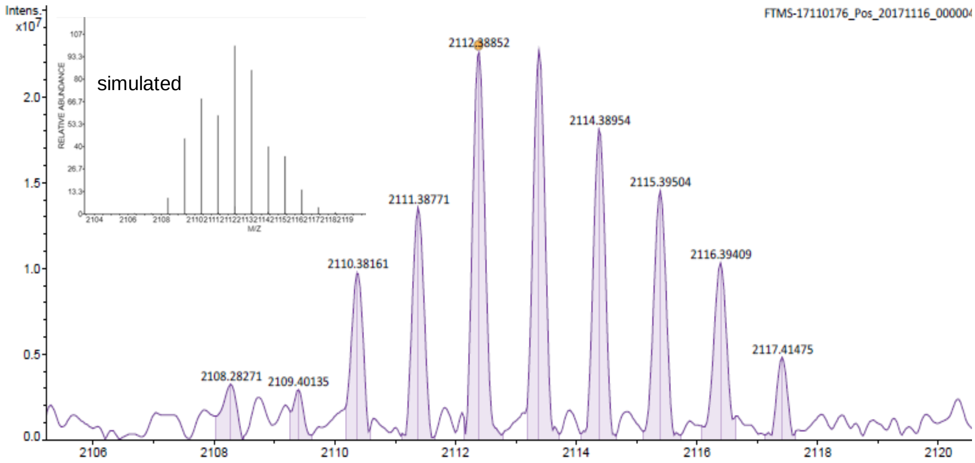

Meas. m/z	#	Ion Formula	Score	m/z	err [ppm]	Mean err [ppm]	mSigma	rdb	e^- Conf	N-Rule
2112.388523	1	C79H66CoD18F8N4O33P3Yb	100.00	2112.394782	3.0	2.4	292.9	38.0	even	-

Fig. S43 HR ESI-MS spectrum of **Yb-1**.

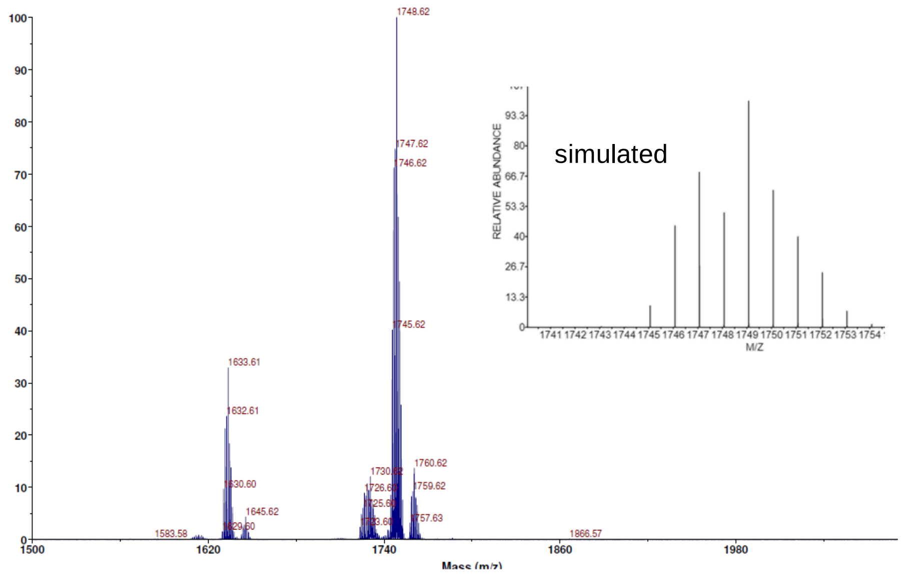

Fig. S44 MALDI-TOF mass spectrum of **Yb-2**.

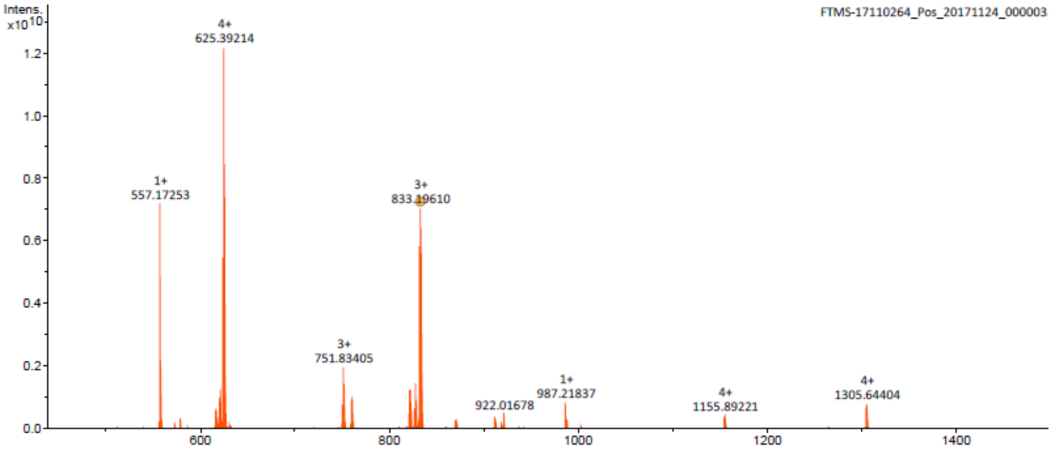

Meas. m/z	#	Ion Formula	Score	m/z	err [ppm]	Mean err [ppm]	mSigma	rdb	e¯ Conf	N-Rule
833.196099	1	C131H89CoD18F8N4O9P7Yb	100.00	833.196919	1.0	5.1	124.2	81.5	odd	-

Fig. S45 HR ESI-MS spectrum of **Yb-3**.

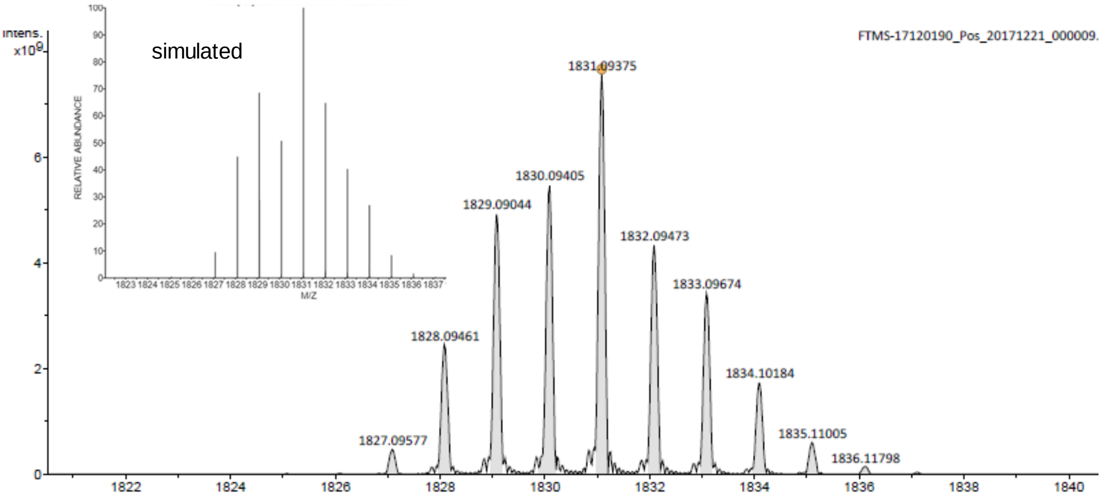

Meas. m/z	#	Ion Formula	Score	m/z	err [ppm]	Mean err [ppm]	mSigma	rdb	e⁻ Conf	N-Rule
1831.093751	1	C59H15CoD18F28N5O9P3Yb	100.00	1831.088893	-2.7	4.3	15.6	34.0	even	-

Fig. S46 HR ESI-MS spectrum of **Yb-4**.

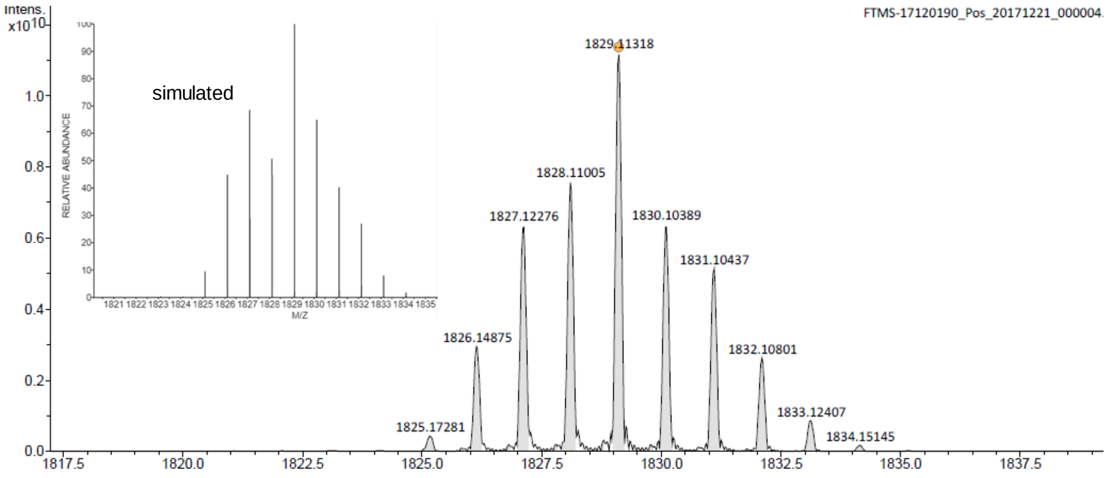

Meas. m/z	#	Ion Formula	Score	m/z	err [ppm]	Mean err [ppm]	mSigma	rdb	e⁻ Conf	N-Rule
1829.113183	1	C59H19CoD18F26N5O11P3Yb	100.00	1829.113216	0.6	0.3	330.6	33.0	even	-

Fig. S47 HR ESI-MS spectrum of **Yb-5**.

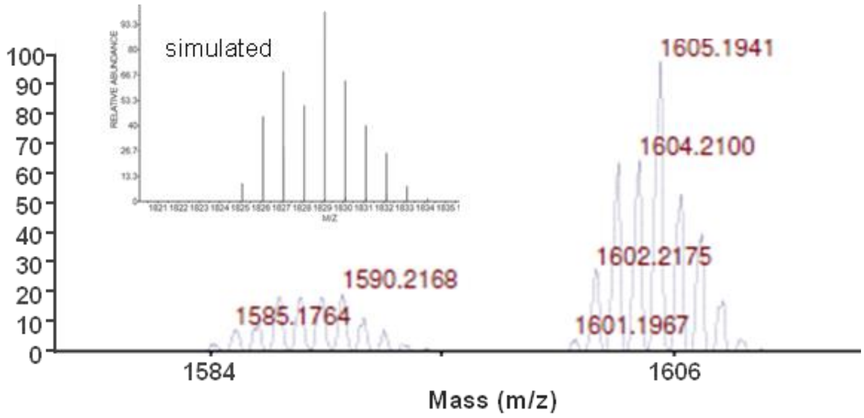

Fig. S48 MALDI-TOF mass spectrum of **Yb-2c**.

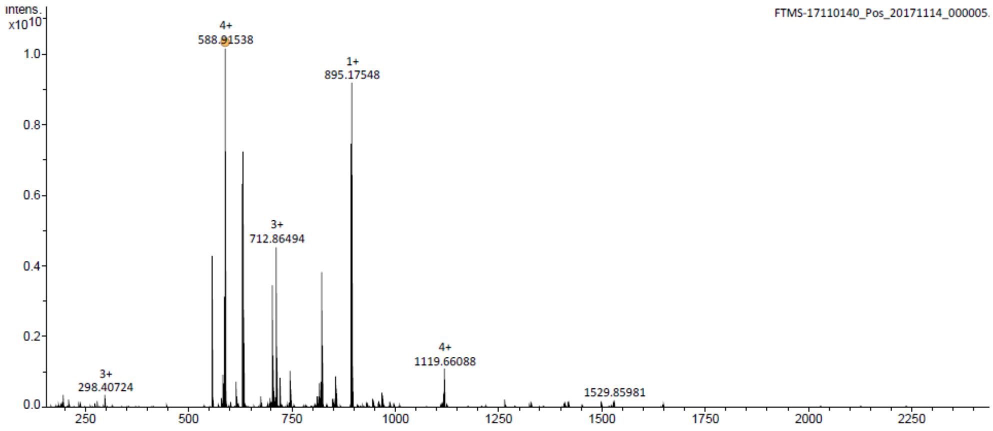

Meas. m/z	#	Ion Formula	Score	m/z	err [ppm]	Mean err [ppm]	mSigma	rdb	e⁻ Conf	N-Rule
588.915377	1	C131H97CoD18N4O9P7Yb	100.00	588.916395	2.8	0.7	79.7	82.0	even	-

Fig. S49 HR ESI-MS spectrum of Yb-**3c**.

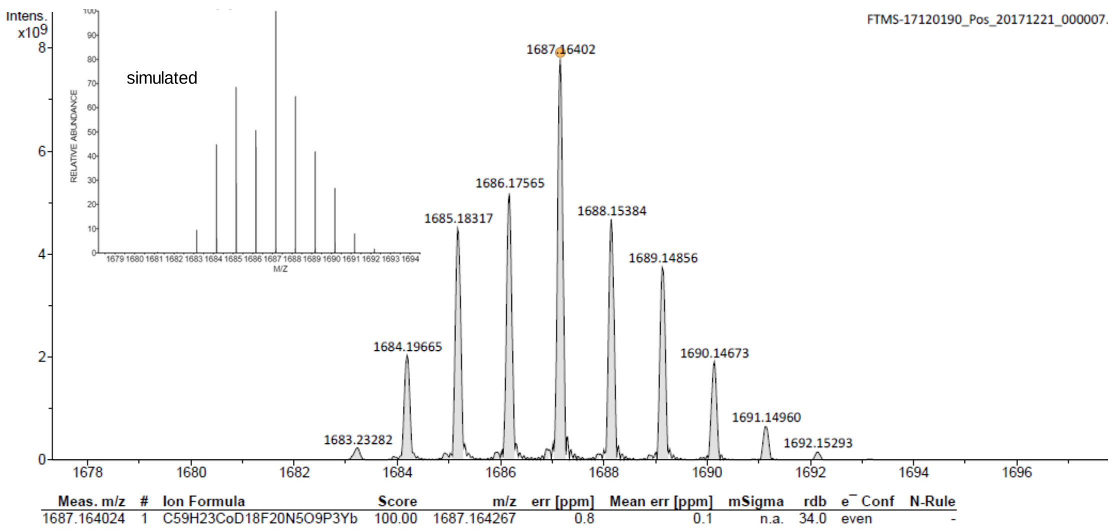

Fig. S50 HR ESI-MS spectrum of **Yb-4c**.

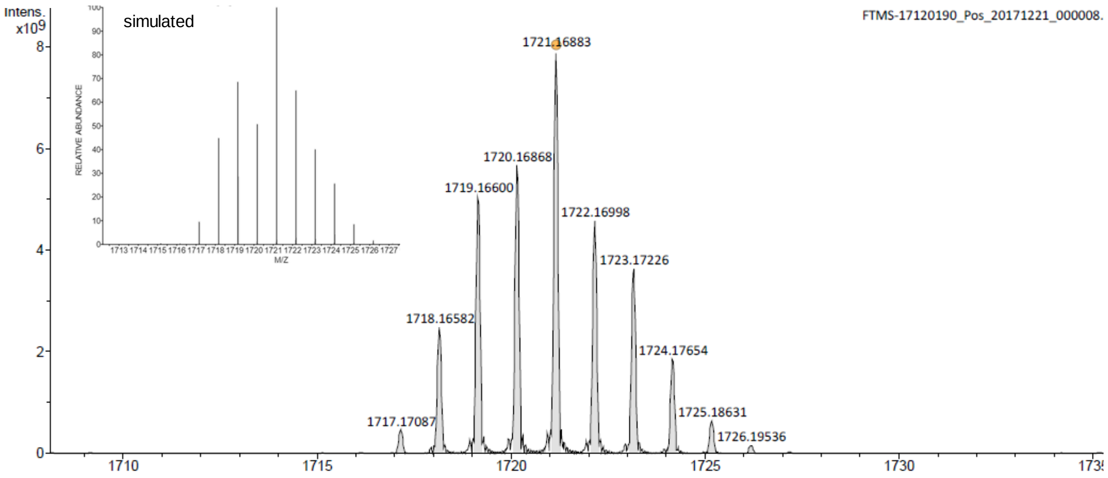

Meas. m/z	#	Ion Formula	Score	m/z	err [ppm]	Mean err [ppm]	mSigma	rdb	e⁻ Conf	N-Rule
1721.168829	1	C59H25CoD18F20N5O11P3Yb	100.00	1721.169746	1.2	0.5	17.2	33.0	even	-

Fig. S51 HR ESI-MS spectrum of **Yb-5c**.

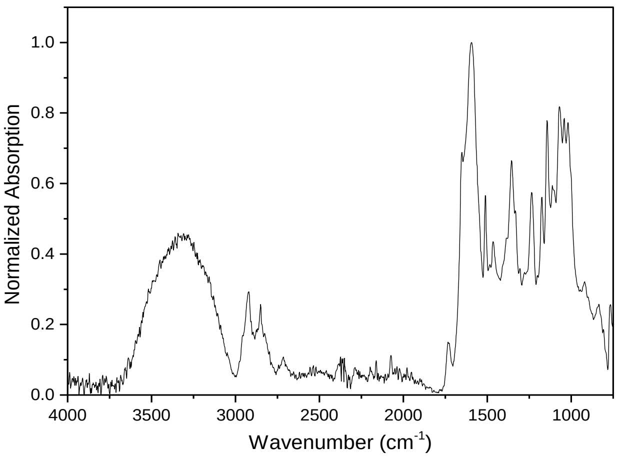

Fig. S52 Normalized FT-IR spectrum of **Yb-1**.

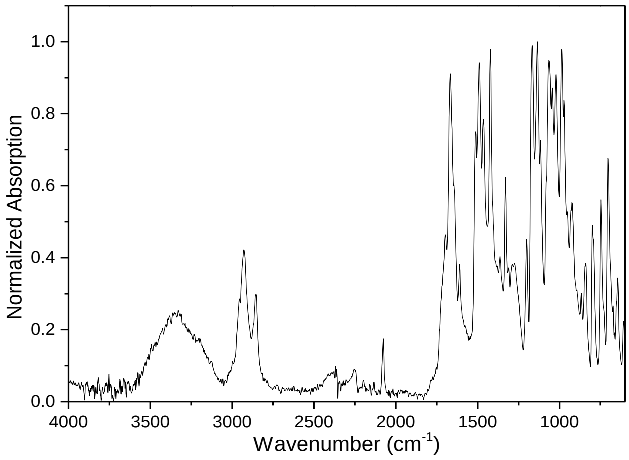

Fig. S53 Normalized FT-IR spectrum of **Yb-2**.

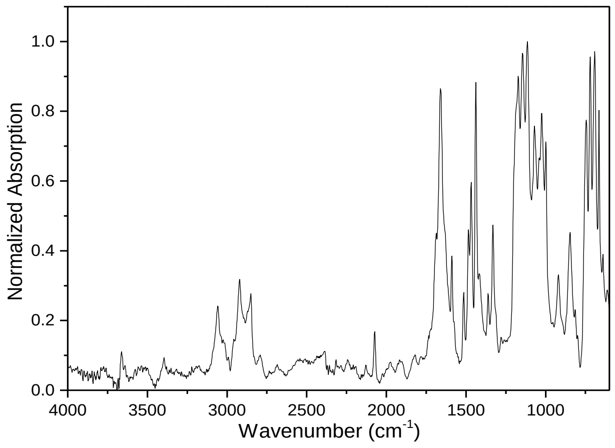

Fig. S54 Normalized FT-IR spectrum of **Yb-3**.

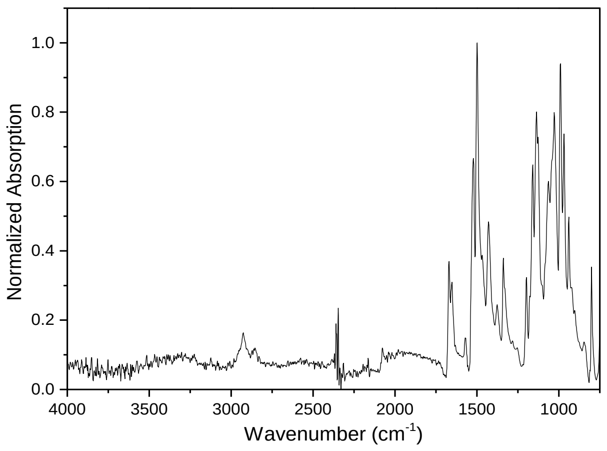

Fig. S55 Normalized FT-IR spectrum of **Yb-4**.

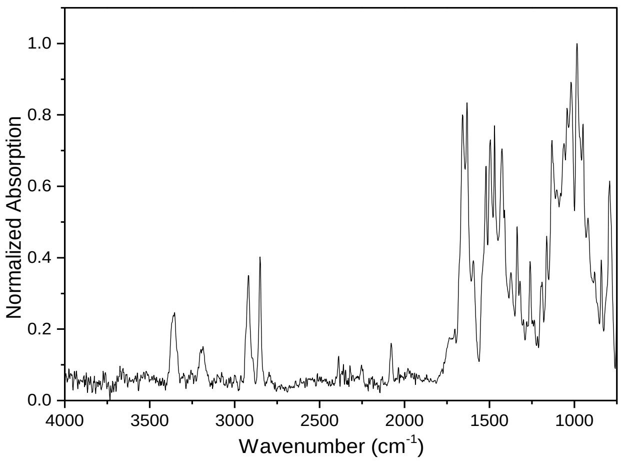

Fig. S56 Normalized FT-IR spectrum of **Yb-5**.

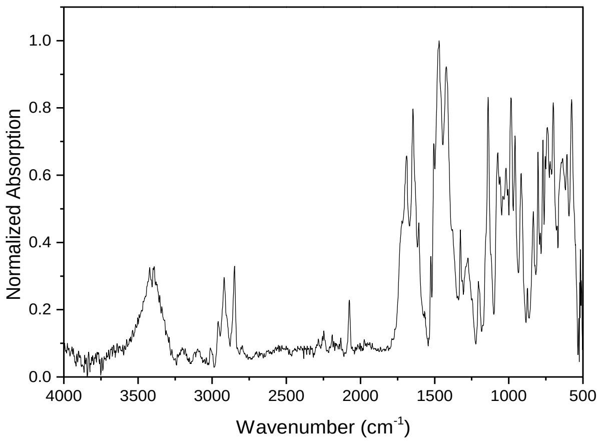

Fig. S57 Normalized FT-IR spectrum of **Yb-2c**.

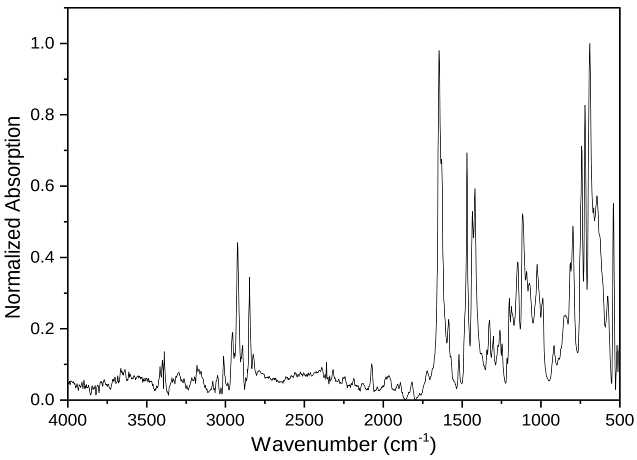

Fig. S58 Normalized FT-IR spectrum of **Yb-3c**.

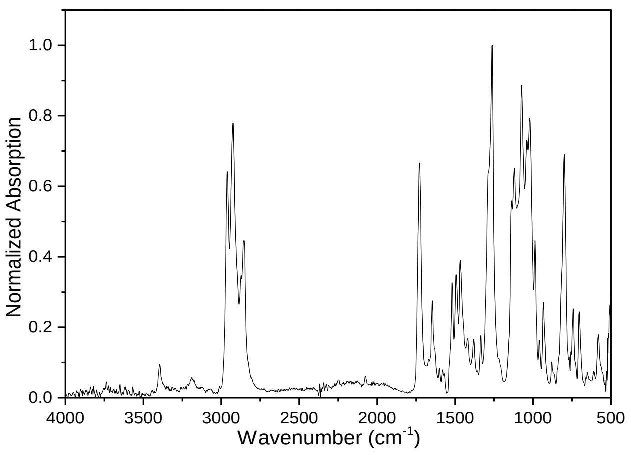

Fig. S59 Normalized FT-IR spectrum of **Yb-4c**.

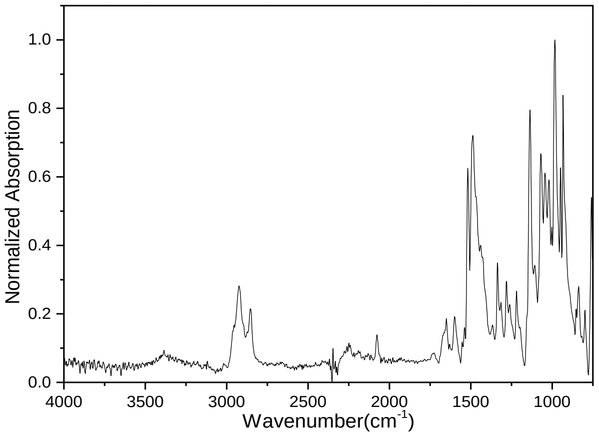

Fig. S60 Normalized FT-IR spectrum of **Yb-5c**.

Supporting information for photophysical property measurements.

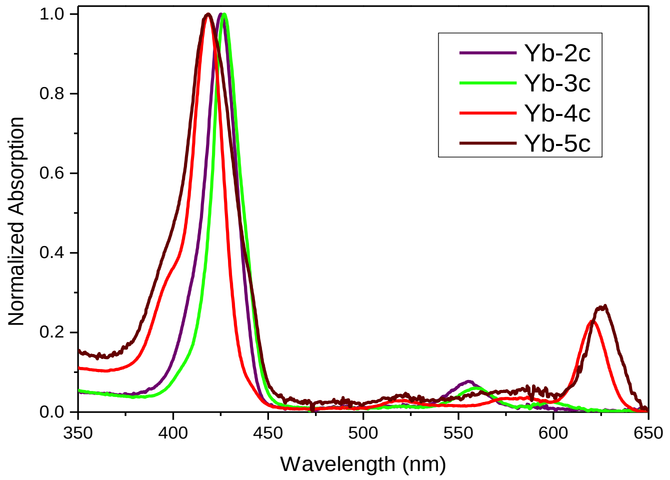

Fig. S61 Normalized absorption spectra of **Yb-2c-5c** in H_2O at room temperature (with 0.1% DMSO).

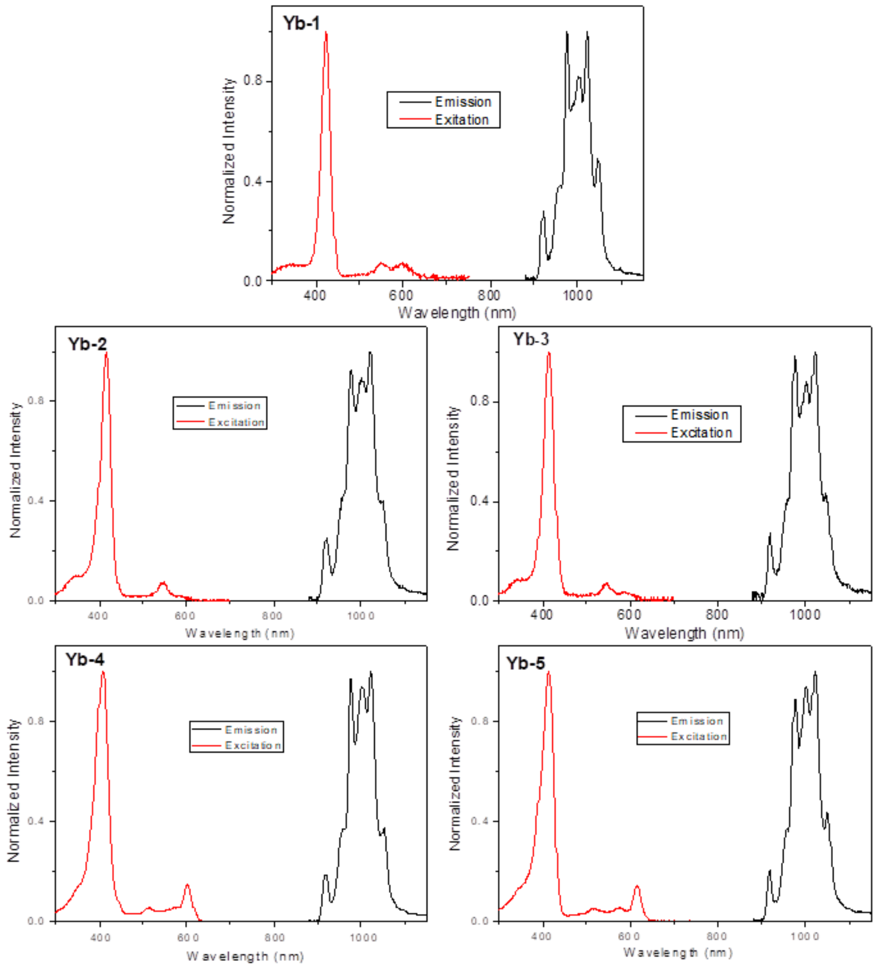

Fig. S62 Normalized excitation and emission spectra of **Yb-1-5** in DMSO at room temperature.

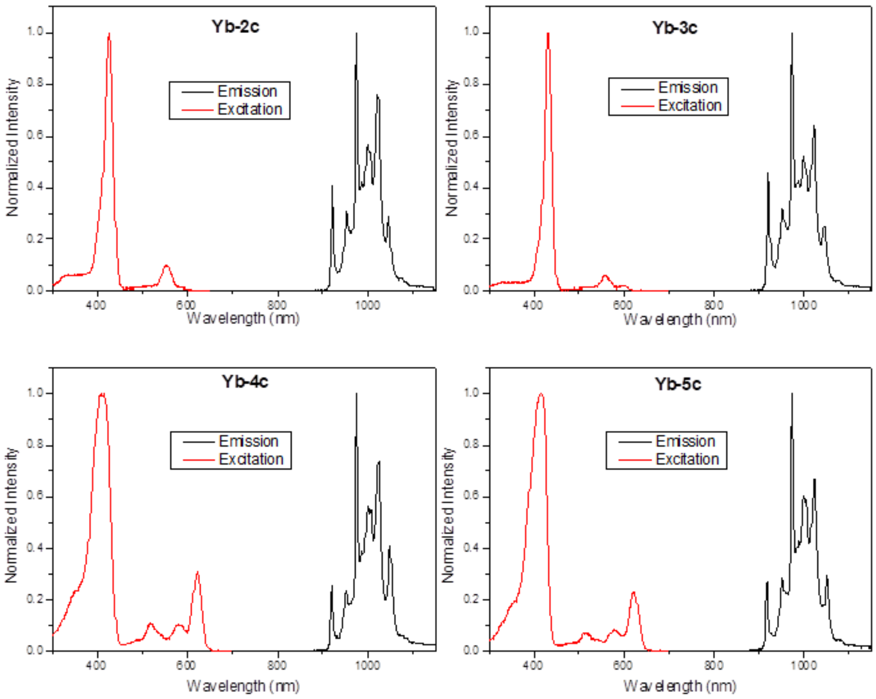

Fig. S63 Normalized excitation and emission spectra of **Yb-2c-5c** in water at room temperature.

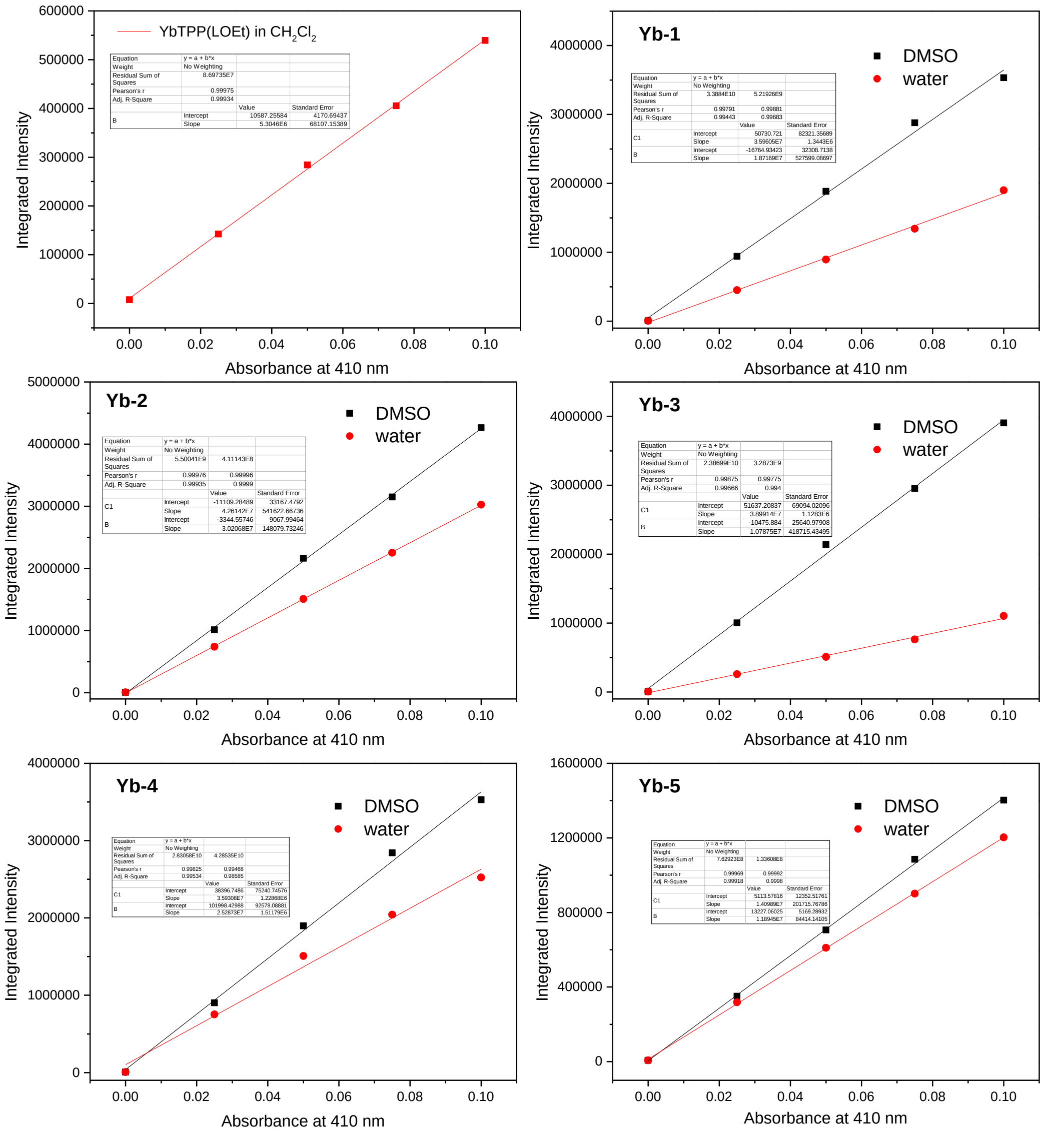

Fig. S64 Integrated emission intensity vs absorbance plots for relative quantum yield determination of **Yb-1-5** vs YbTPP(LOEt) (λ_{ex} = 410 nm, Φ_r = 0.024) in DMSO and H_2O at room temperature.

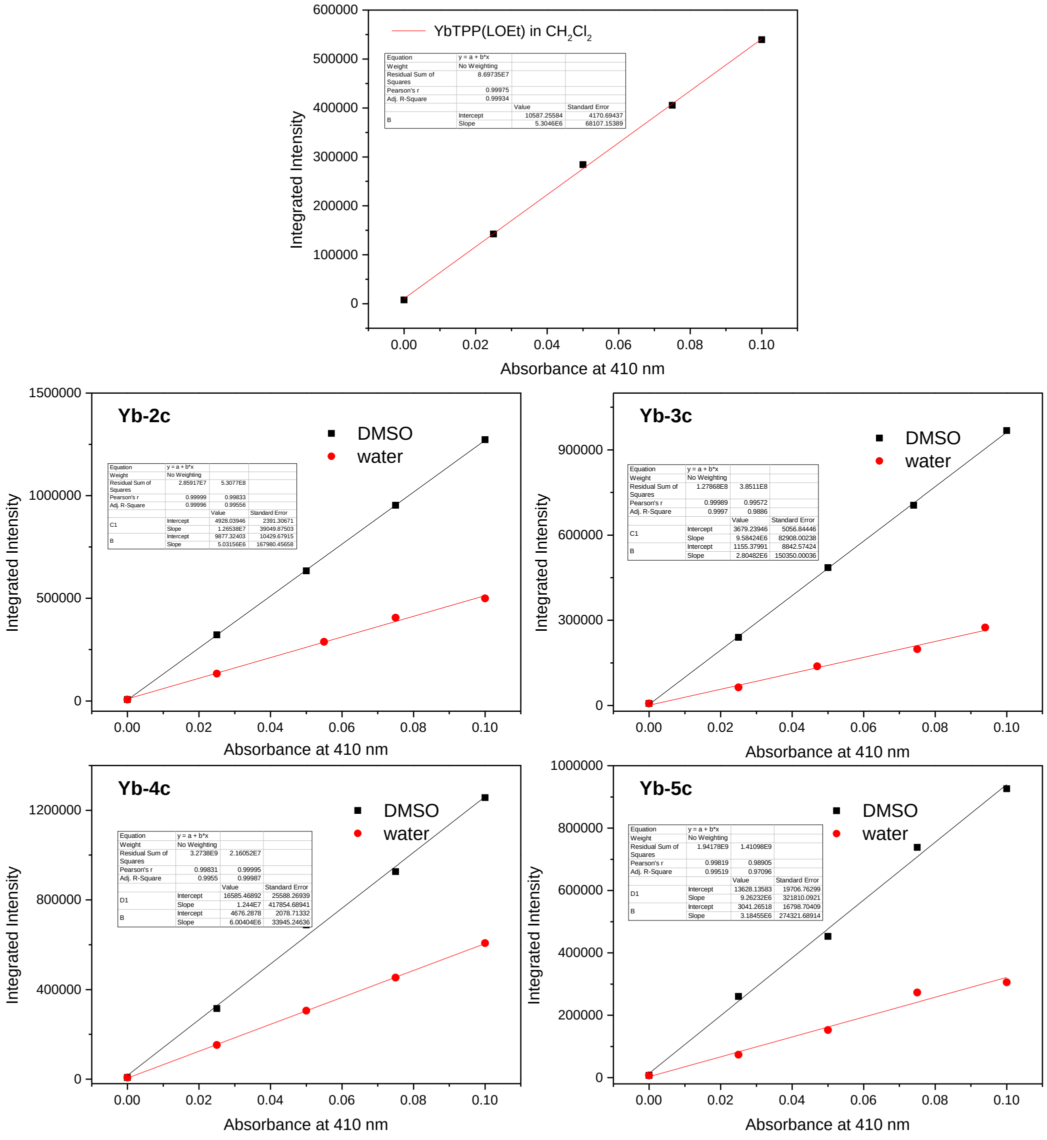

Fig. S65 Integrated emission intensity vs absorbance plots for relative quantum yield determination of **Yb-2c-5c** vs YbTPP(LOEt) (λ_{ex} = 410 nm, Φ_r = 0.024) in DMSO and H_2O at room temperature.

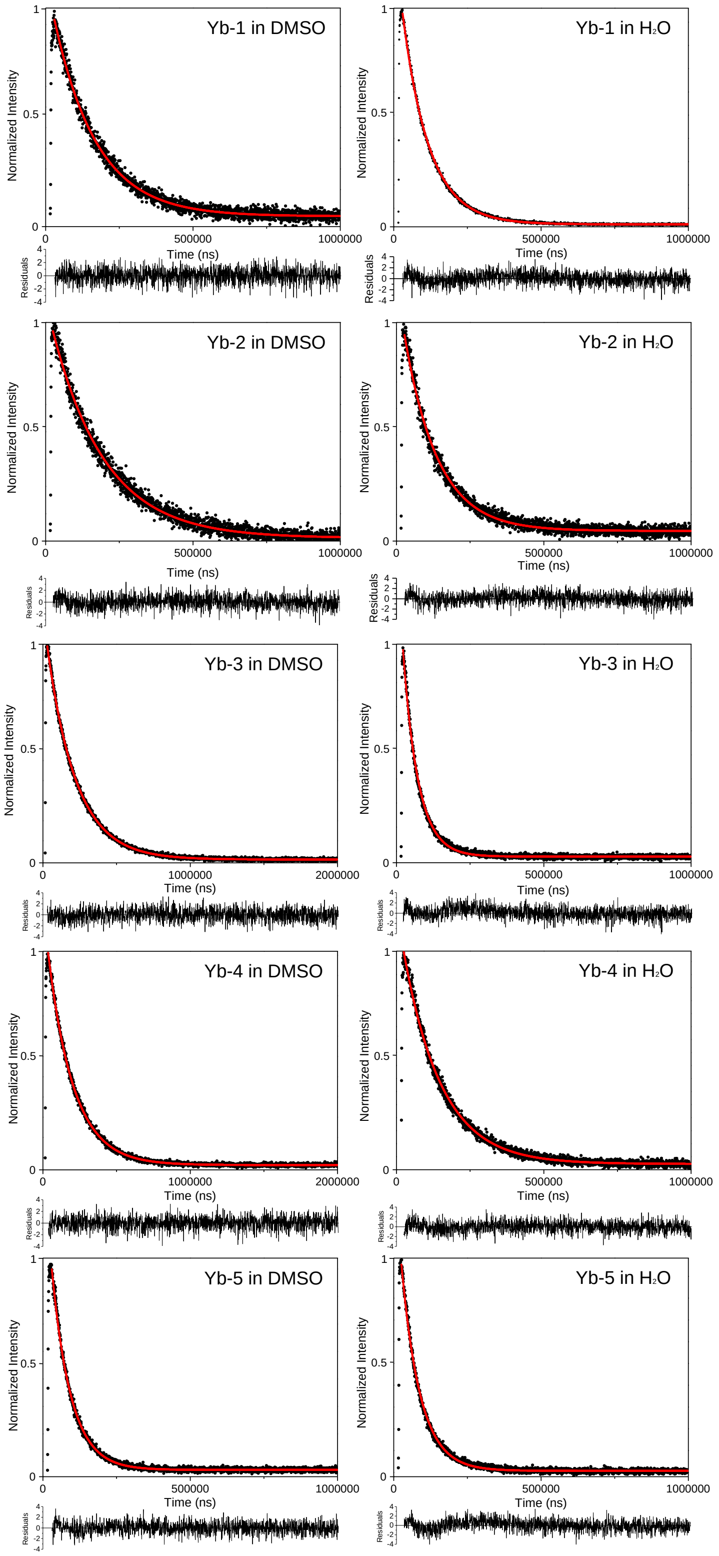

Fig. S66 NIR luminescence decay curve monitored at 980 nm **Yb-1-5** in DMSO and H_2O at room temperature (λ_{ex} = 410 nm, $OD_{410\ nm}$ = 0.1).

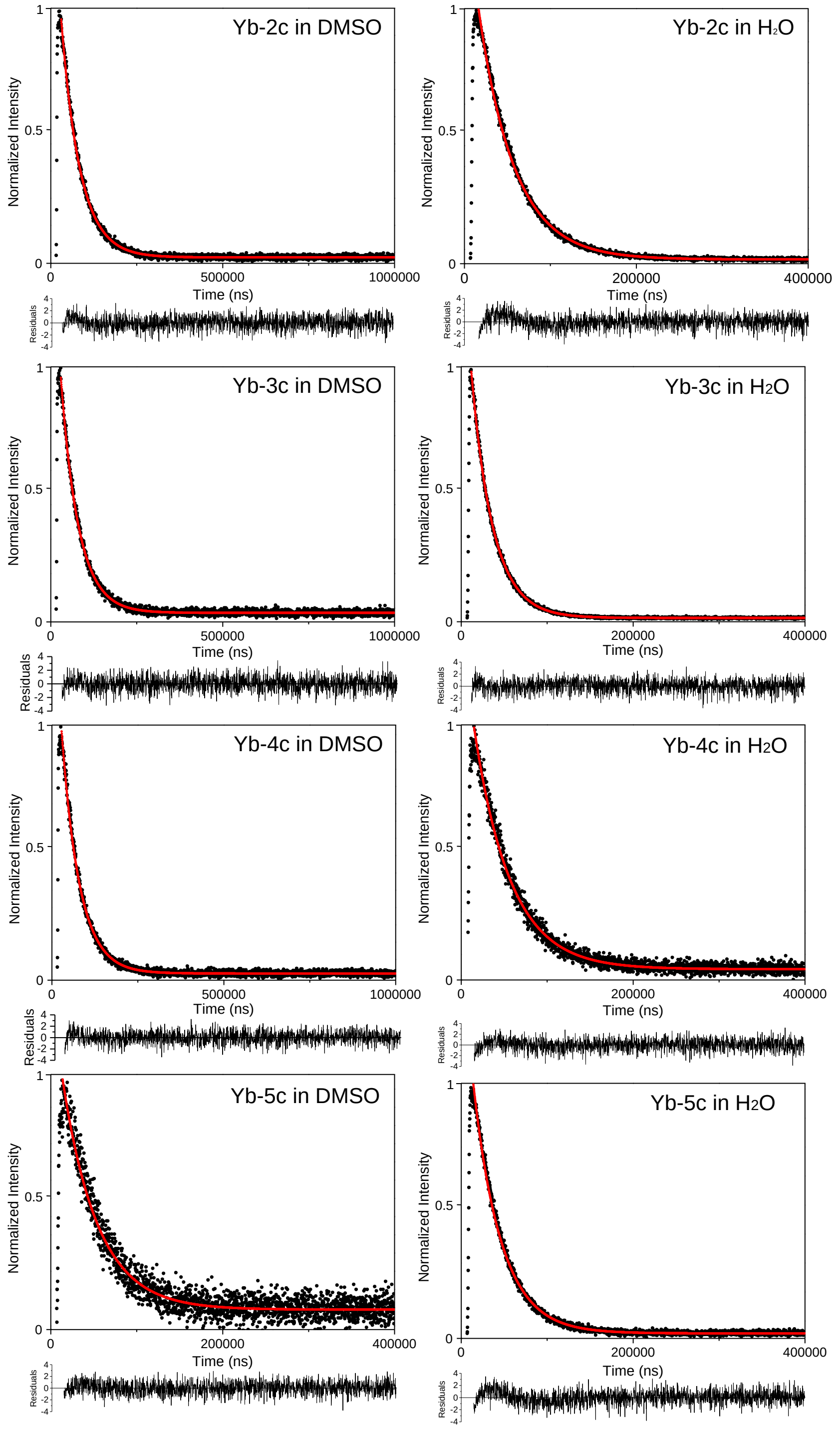

Fig. S67 NIR luminescence decay curve monitored at 980 nm **Yb-2c-5c** in DMSO and H_2O at room temperature (λ_{ex} = 410 nm, $OD_{410\ nm}$ = 0.1).

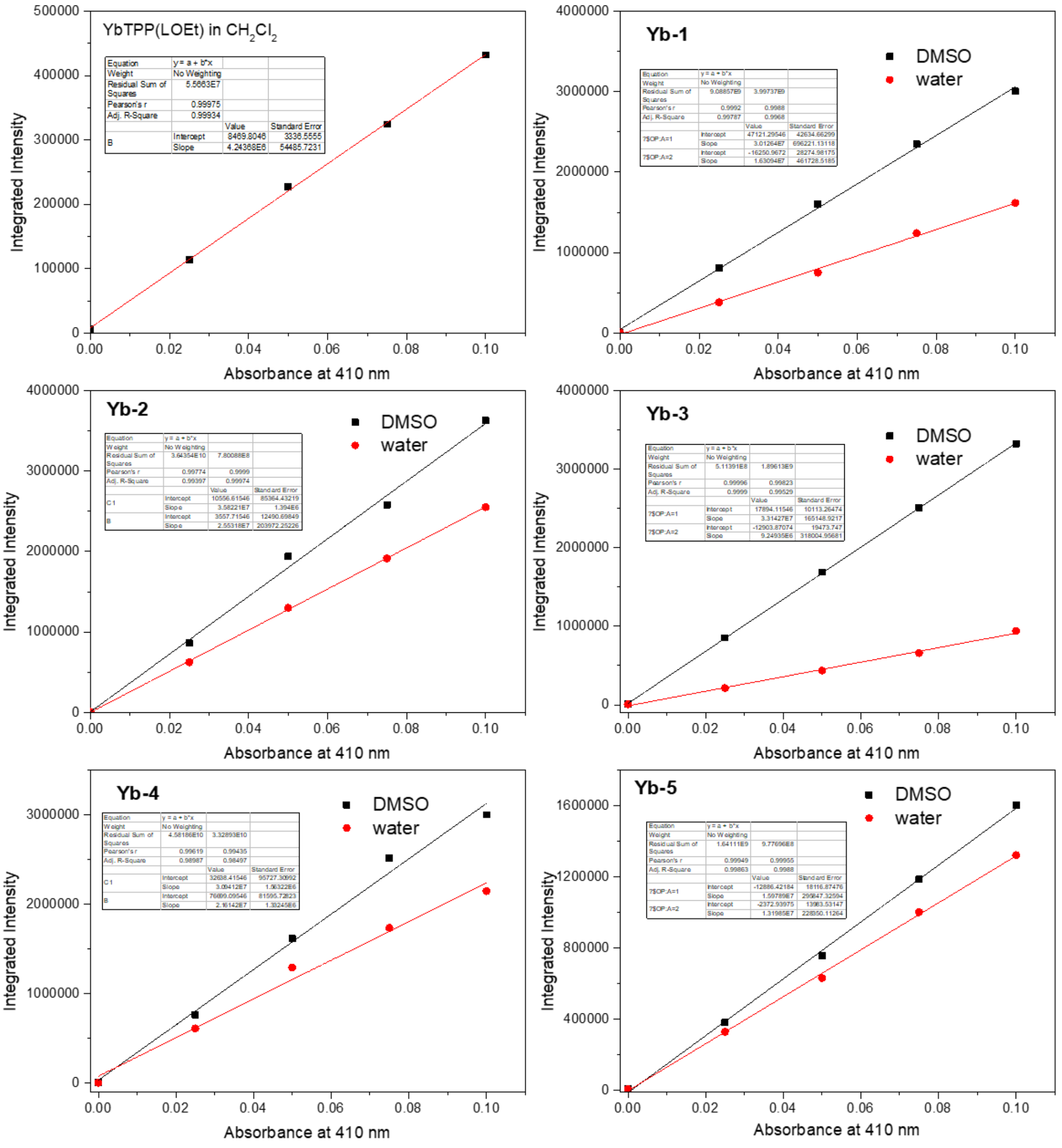

Fig. S68 Integrated emission intensity vs absorbance plots for relative quantum yield determination of **Yb-1-5** vs YbTPP(LOEt) (λ_{ex} = 410 nm, Φ_r = 0.024) in degassed DMSO and H_2O at room temperature.

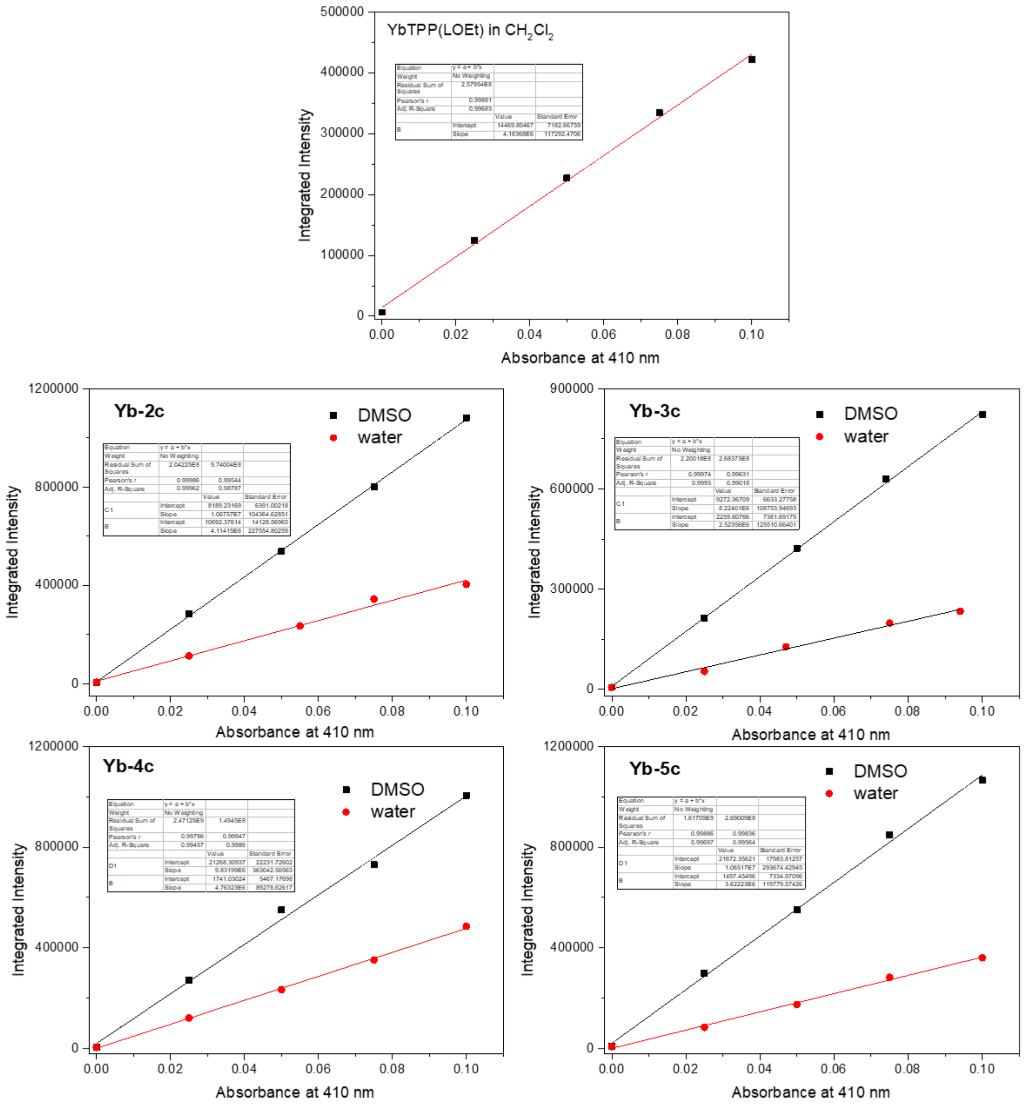

Fig. S69 Integrated emission intensity vs absorbance plots for relative quantum yield determination of **Yb-2c-5c** vs YbTPP(LOEt) (λ_{ex} = 410 nm, Φ_r = 0.024) in degassed DMSO and H_2O at room temperature.

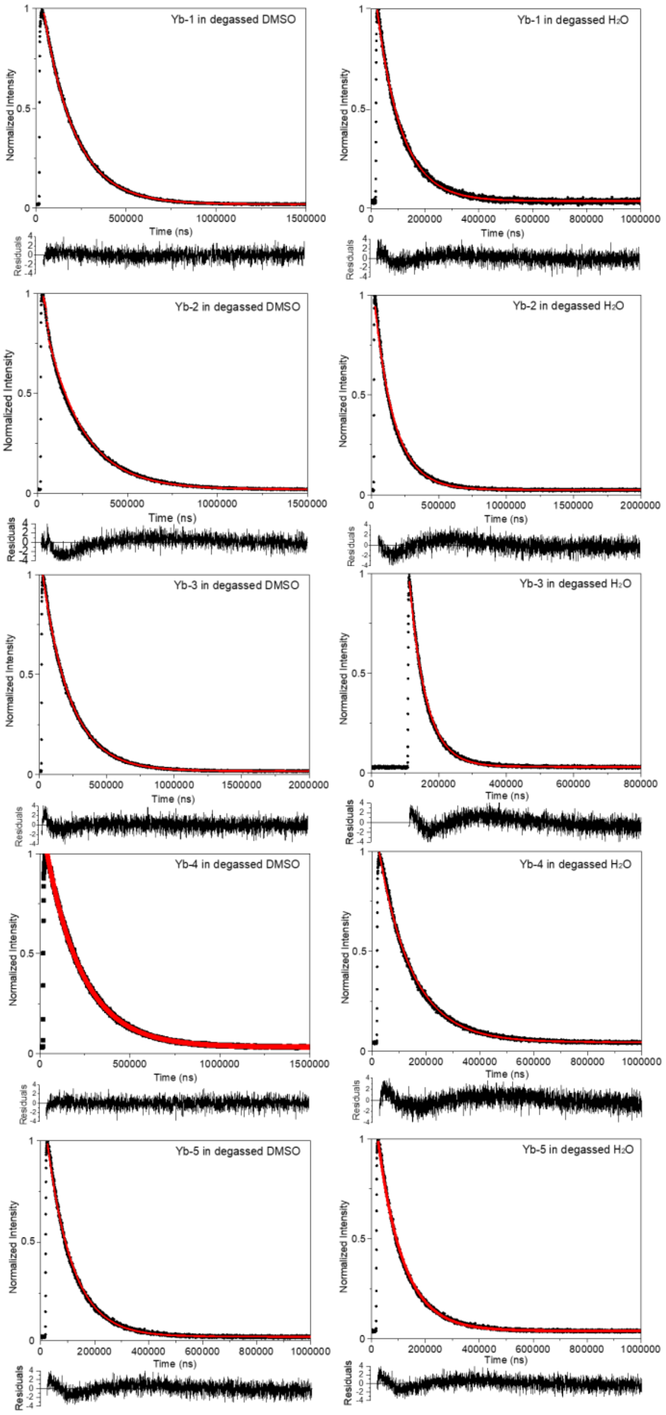

Fig. S70 NIR luminescence decay curve monitored at 980 nm **Yb-1-5** in degassed DMSO and H_2O at room temperature (λ_{ex} = 410 nm, $OD_{410\ nm}$ = 0.1).

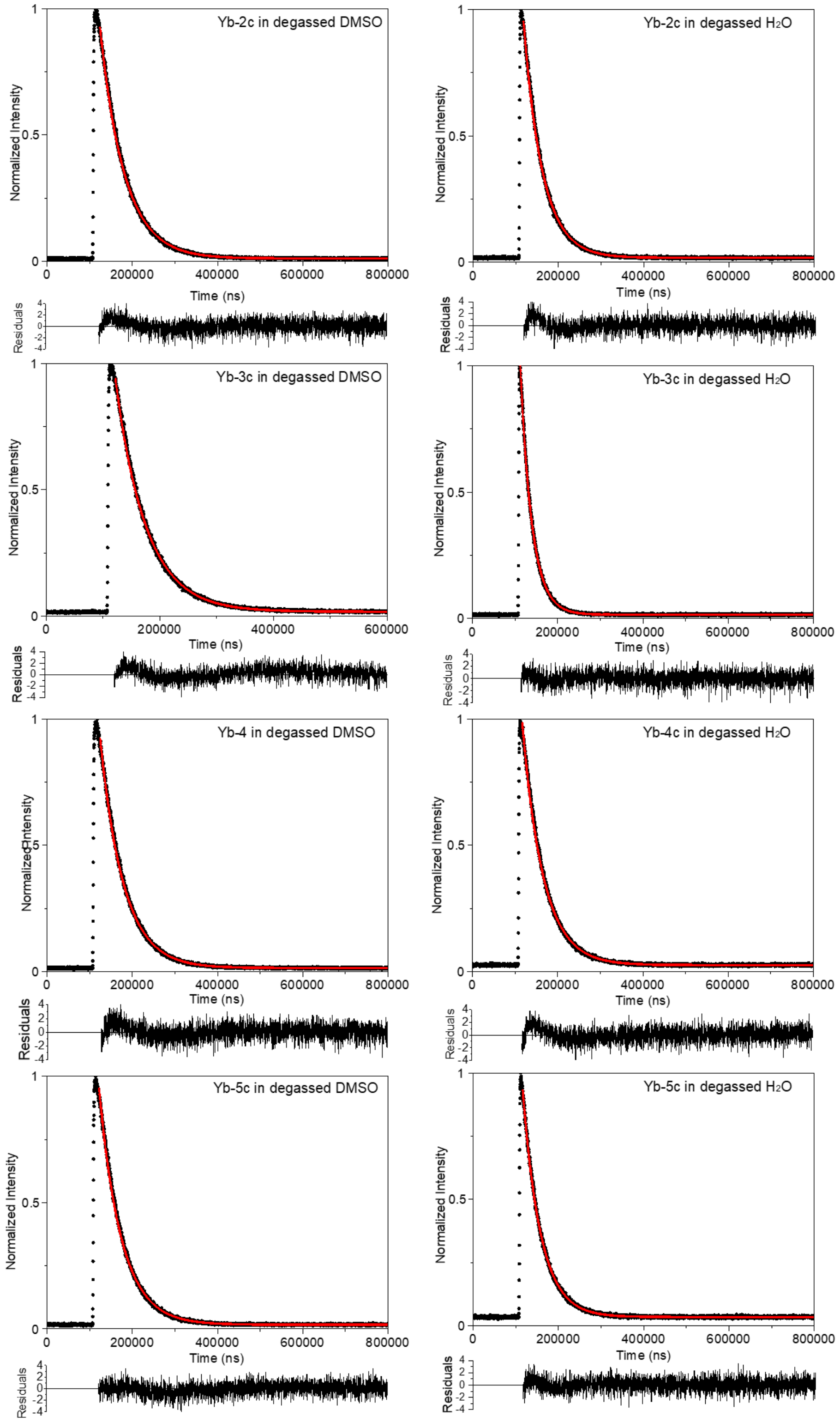

Fig. S71 NIR luminescence decay curve monitored at 980 nm **Yb-2c-5c** in degassed DMSO and H_2O at room temperature (λ_{ex} = 410 nm, $OD_{410\ nm}$ = 0.1).

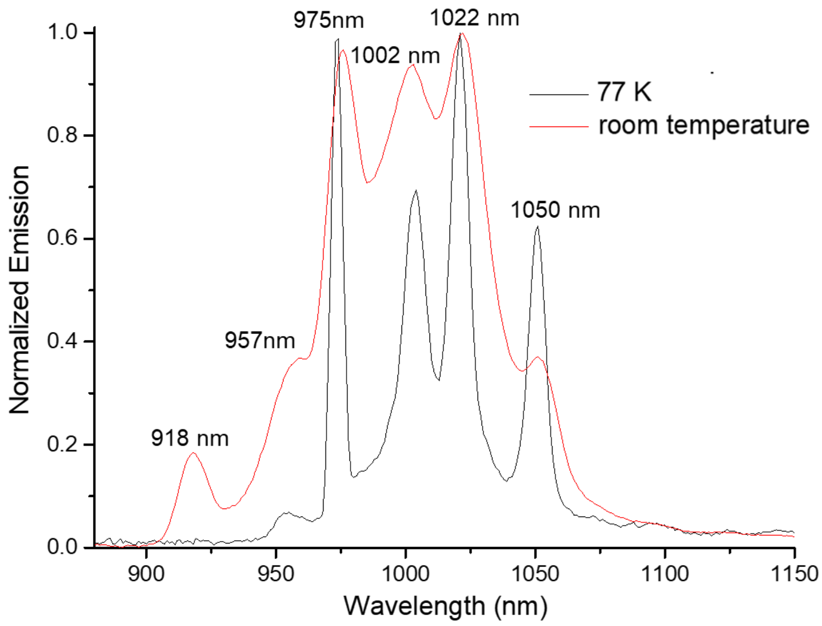

Fig. S72 Normalized emission intensity of **Yb-4** in DMSO at room temperature and MeOH/EtOH (v/v = 1/1) at 77 K.

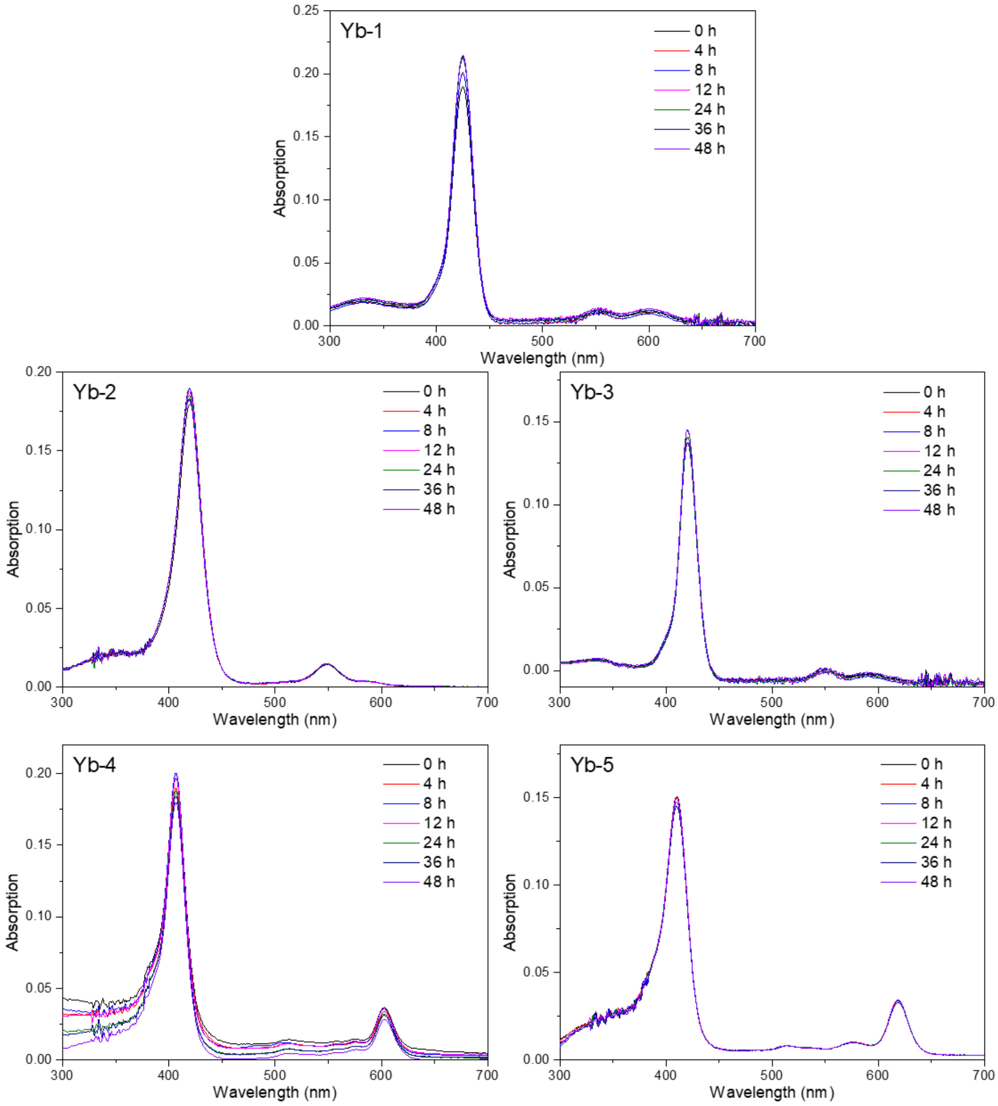

Fig. S73 Stabilities of **Yb-1-5** in DMSO.

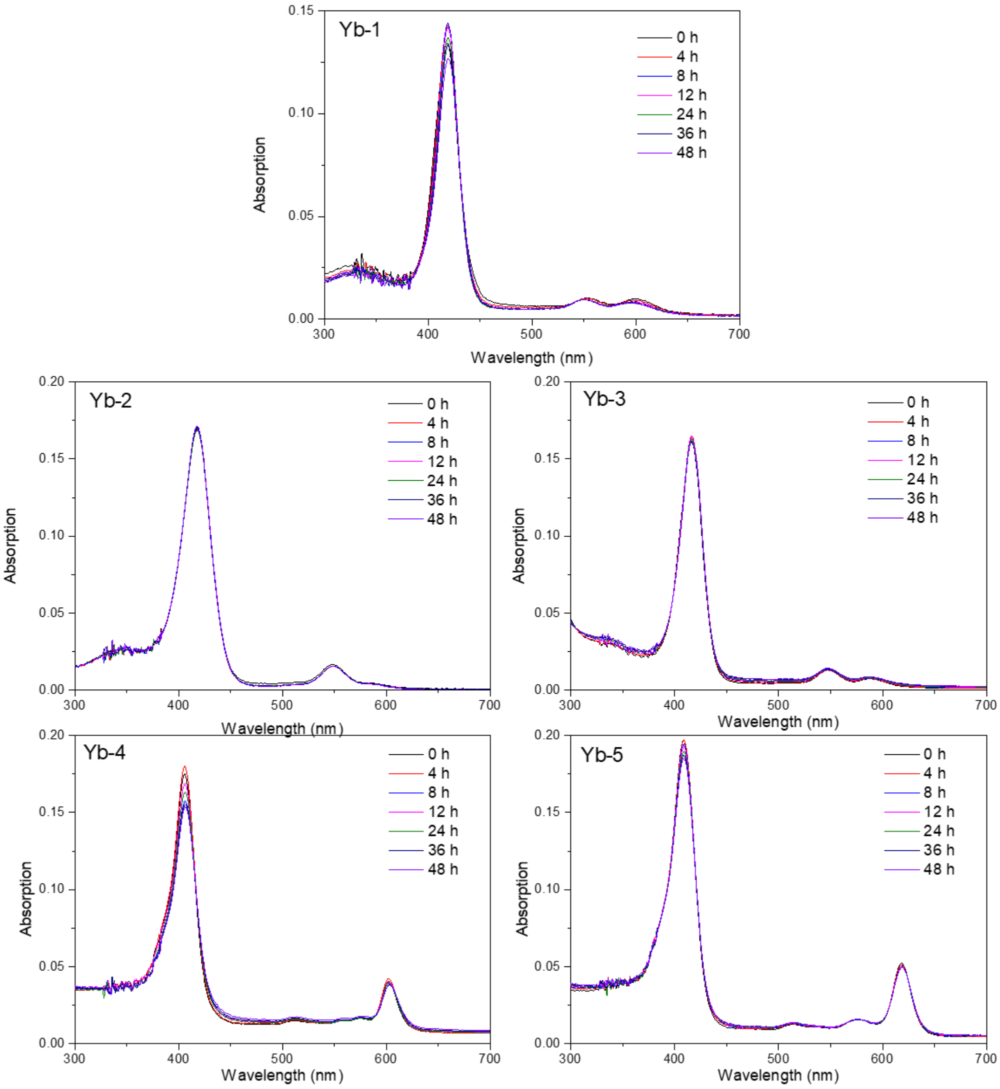

Fig. S74 Stabilities of **Yb-1-5** in H_2O with 1% DMSO.

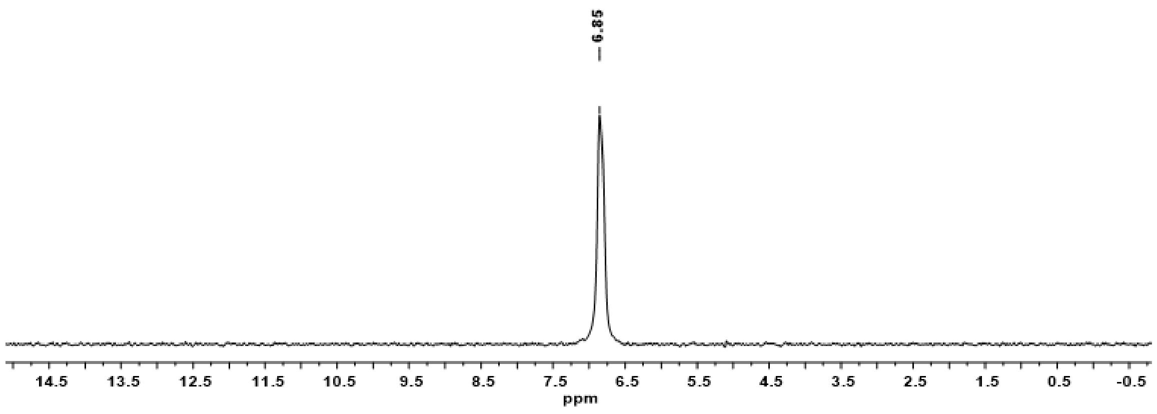

Fig. S75 ^{2}D NMR (77 MHz) of **Yb-4** in CH_3OH.

Supporting information for singlet oxygen quantum yield determination and cell experiments

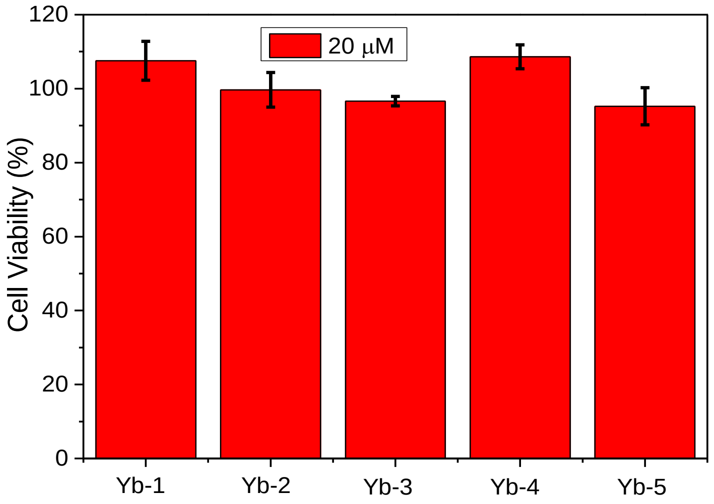

Fig. S76 Dark cytotoxicity of 20 μM **Yb-1-5** toward Hela cells.

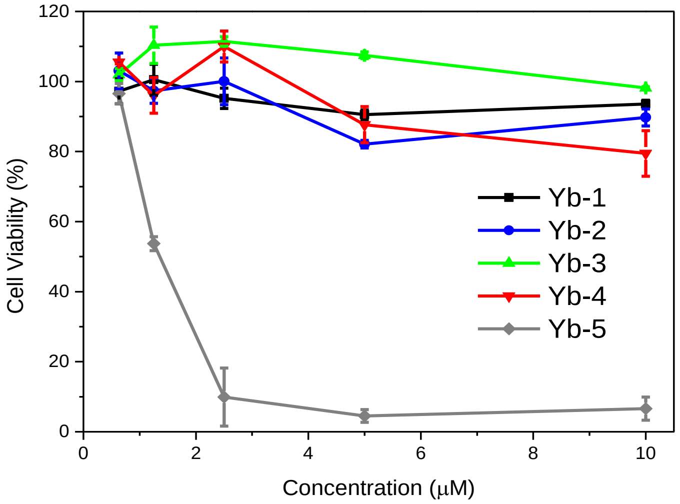

Fig. S77 Photocytotoxicity of **Yb-1-5** toward Hela cells using CCK-8 assay under the light irradiation (400–700 nm, 6.5 mW/cm^2) for 30 min, and then cultured in the dark for another 24 hours.

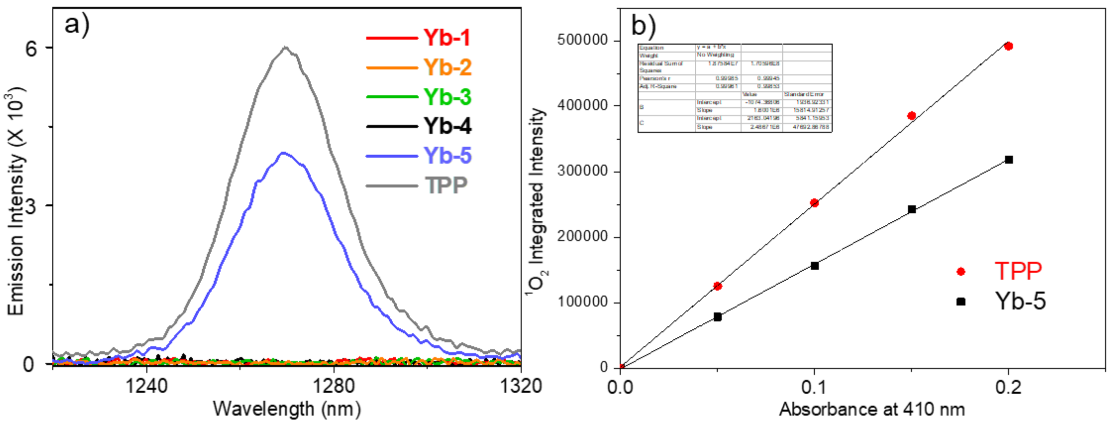

Fig. S78 a) NIR emission of $^1O_2$ at 1270 nm excited at 410 nm in the $CHCl_3$ solution of **Yb-1-5**; b) Relative intensity of singlet oxygen production vs absorption for **Yb-5** and TPP at 410 nm.

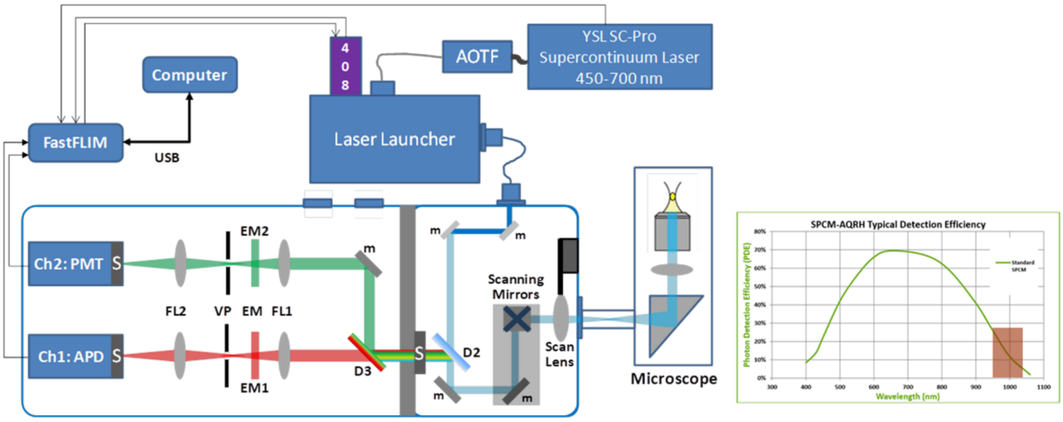

Fig. S79 Experimental setup for NIR living cell imaging. D: dichroic mirror, Filter 1: 935/170 nm band pas; Filter 2: 530/43 nm bandpass, PMT: photomultiplier, APD: avalanche photodiode

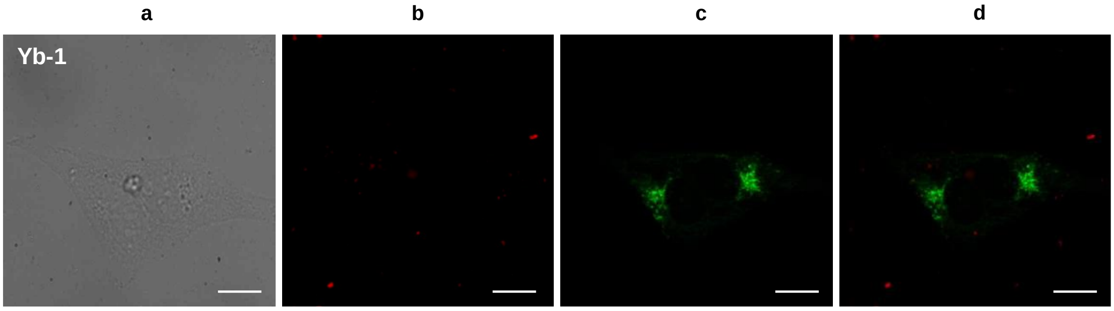

Fig. S80 Images obtained from confocal fluorescence experiments performed on HeLa cells incubated with 10 μM **Yb-1** for 12 h followed by 30 min incubation with 75 nM solution of LysoTracker Green. a) Bright field; b) NIR signal arising from Yb(III) (λ_{ex}, 408 nm; λ_{em}, 935/170 nm bandpass); c) visible signal arising from LysoTracker Green (λ_{ex}, 470 nm; λ_{em}, 530/43 nm bandpass); d) merged b and c. Scale bar: 10 μm.

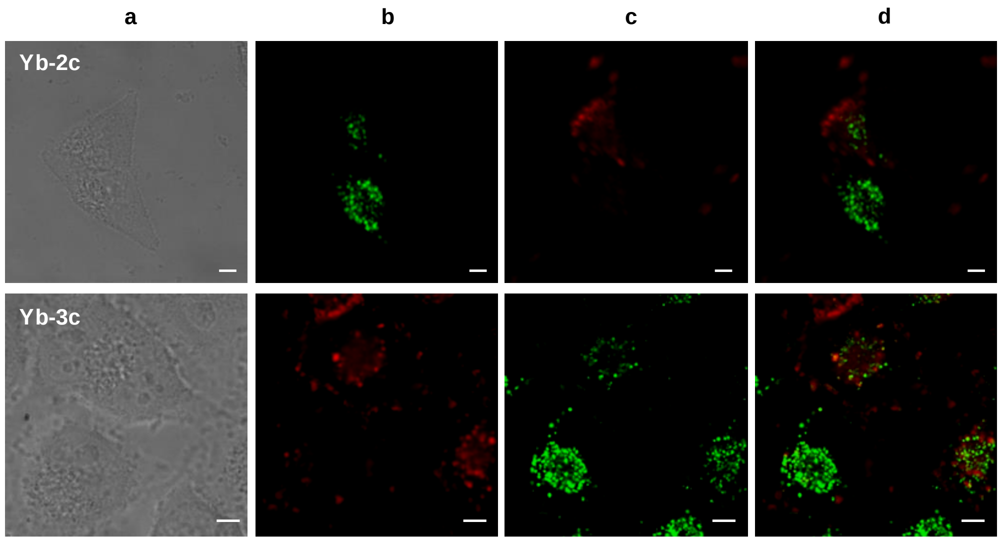

Fig. S81 Images obtained from confocal fluorescence experiments performed on HeLa cells incubated with 10 μM **Yb-2c** and **Yb-3c** for 12 h followed by 30 min incubation with 75 nM solution of Lysotracker Green. a) Bright field; b) NIR signal arising from Yb(III) (λ_{ex}, 408 nm; λ_{em}, 935/170 nm bandpass); c) visible signal arising from Lysotracker Green (λ_{ex}, 470 nm; λ_{em}, 530/43 nm band pass); d) merged b and c. Scale bar: 10 μm.

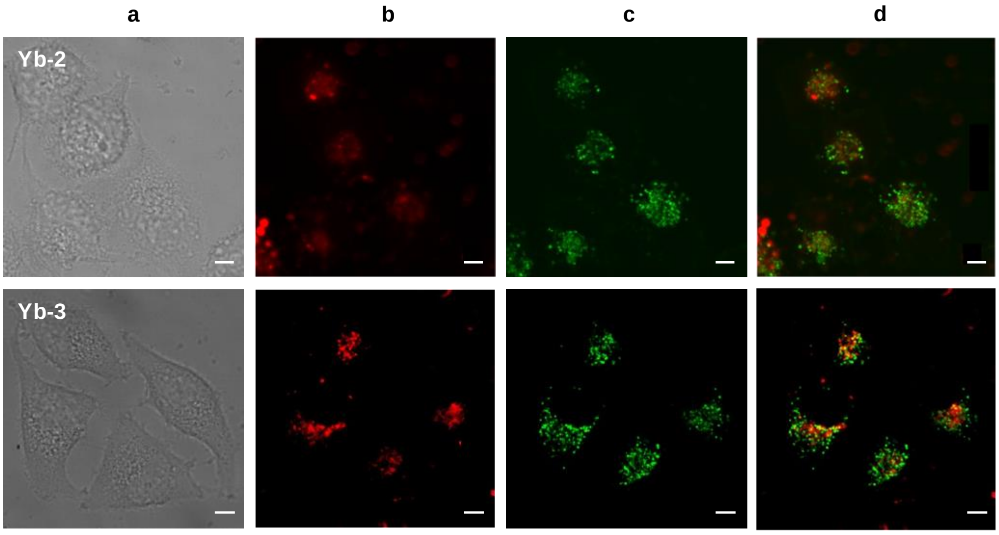

Fig. S82 Images obtained from confocal fluorescence experiments performed on HeLa cells incubated with 10 μM **Yb-2** and **Yb-3** for 12 h followed by 30 min incubation with 75 nM solution of LysoTracker Green. a) Bright field; b) NIR signal arising from Yb(III) (λ_{ex}, 600 nm; λ_{em}, 935/170 nm bandpass); c) visible signal arising from LysoTracker Green (λ_{ex}, 470 nm; λ_{em}, 530/43 nm bandpass); d) merged b and c. Scale bar: 10 μm.

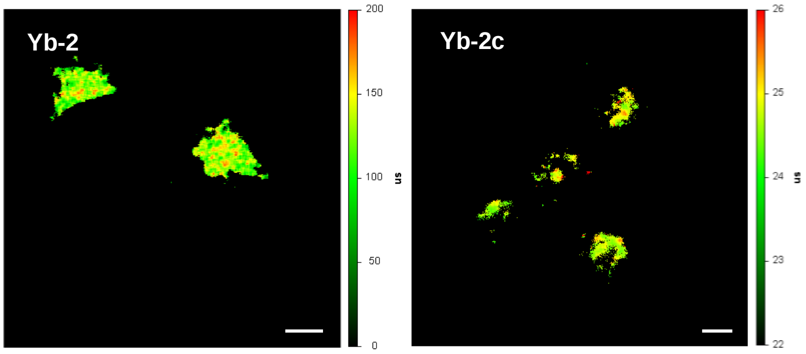

Fig. S83 NIR time-resolved image of live HeLa cells incubated with 10 μM **Yb-2** and **Yb-2c**. a) Bright field; b) NIR signal arising from Yb(III) (λ_{ex}, 408 nm; λ_{em}, 935/170 nm bandpass; dwell time, 4 ms). Scale bar: 10 μm.

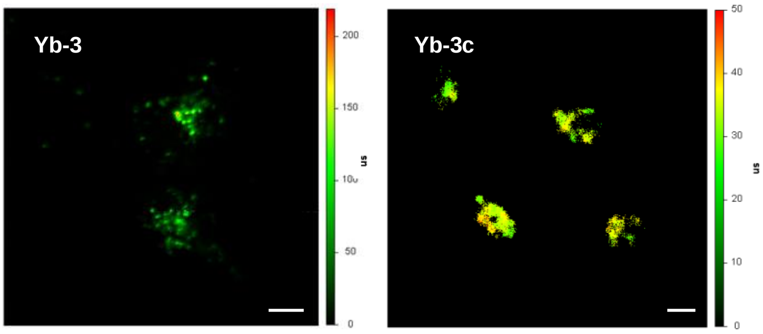

Fig. S84 NIR time-resolved image of live HeLa cells incubated with 10 µM **Yb-3** and **Yb-3c**. a) Bright field; b) NIR signal arising from Yb(III) (λ_{ex}, 408 nm; λ_{em}, 935/170 nm bandpass; dwell time, 4 ms). Scale bar: 10 µm.

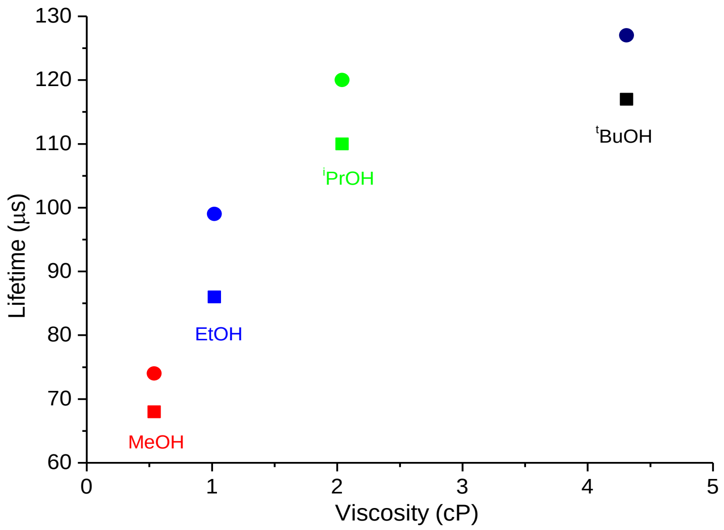

Fig. S85 The decay lifetimes of **Yb-4** monitored at 980 nm relative to the solvent viscosity in MeOH, EtOH, ^{i}PrOH and ^{t}BuOH. Squares represent data in air-equilibrated solutions and circles in degassed solutions at 298 K (λ_{ex} = 410 nm).

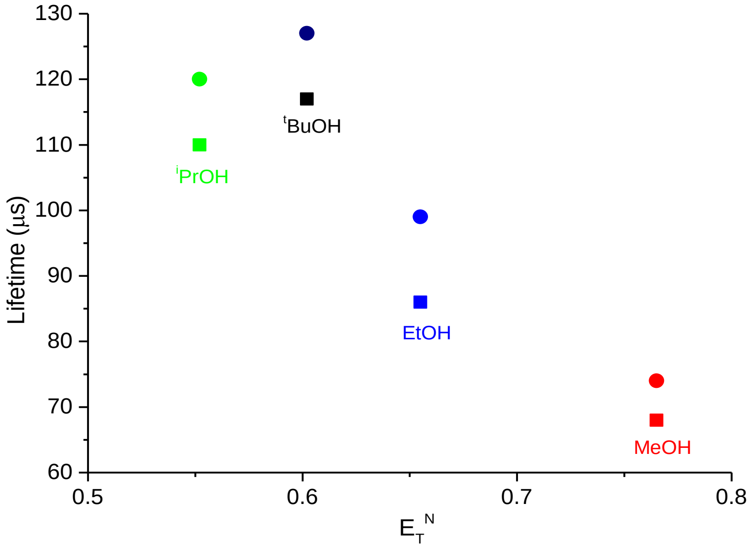

Fig. S86 The decay lifetimes of **Yb-4** monitored at 980 nm relative to Reichardt's normalised solvent polarity parameter (E_T^N) in MeOH, EtOH, ^{i}PrOH and ^{t}BuOH. Squares represent data in air-equilibrated solutions and circles in degassed solutions at 298 K (λ_{ex} = 410 nm).

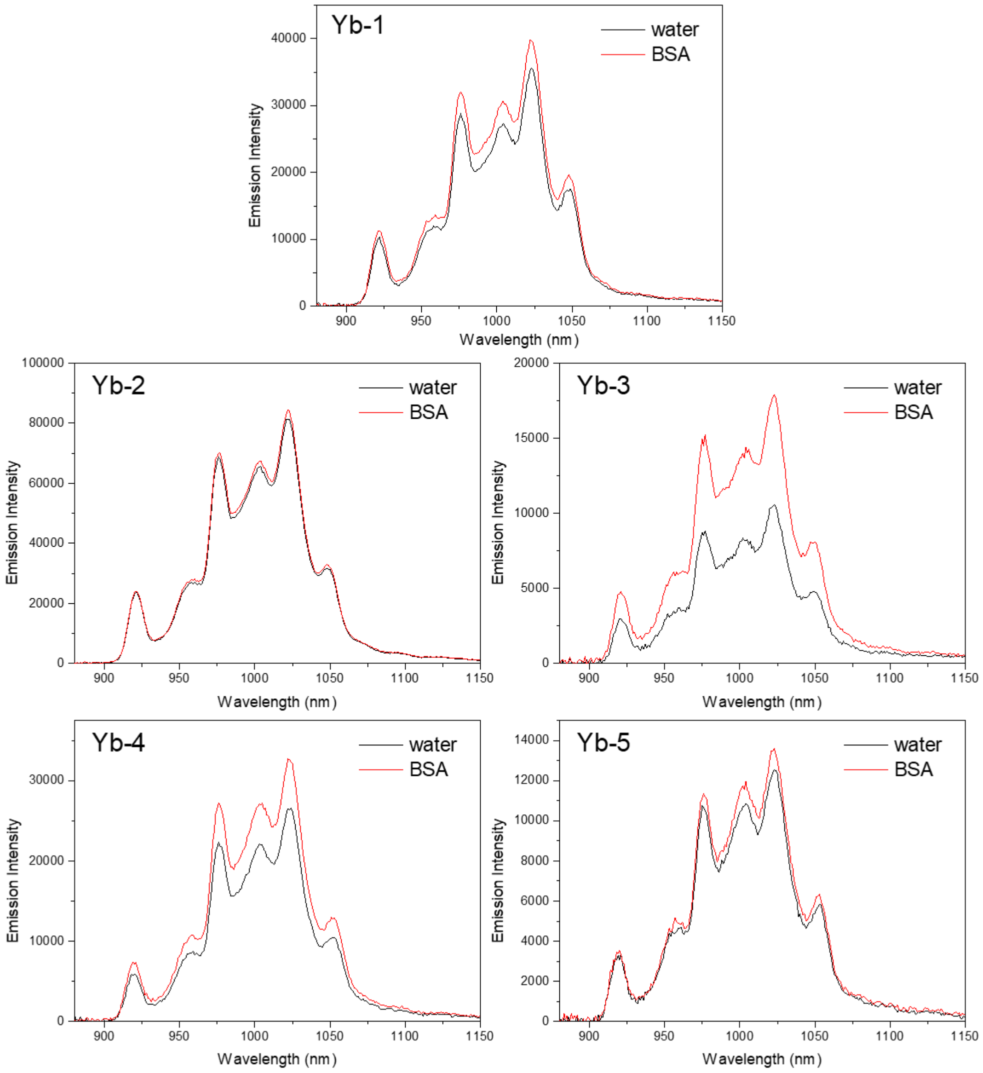

Fig. S87 Emission Intensity of **Yb-1-5** in water and 0.2 M bovine albumin (BSA) (λ_{ex} = 410 nm, $A_{410\ nm}$ = 0.1).
